# Supplementary material for: Community psychosocial music intervention (CHIME) to reduce antenatal common mental disorder symptoms in The Gambia: a feasibility trial
Source: BMJ Open. 2020 Nov 23;10(11):e040287. doi: 10.1136/bmjopen-2020-040287 (PMC7684808; doi:10.1136/bmjopen-2020-040287)
Supplement: Supplementary data [file bmjopen-2020-040287supp001.pdf]

Supplementary Material 1

Intervention Development Workshop Discussion points

These six sessions are intended to support women emotionally and psychologically during their pregnancy.

Session Goals

During the session, we hope that women will participate by singing songs, clapping hands, and moving to the music. Also, we would like you to share some brief information about how to cope with common physical and psychological symptoms of pregnancy. We hope that the women will finish the session

- in a *positive mood*
- feeling part of a socially-supportive group
- with some new strategies of how to cope with some of the common physical and psychological challenges of pregnancy
- equipped with skills to seek wider social support even outside the sessions

Key Messages

Our suggestion is that sessions concentrate on various different themes as follows [discuss with the Kanyeleng group whether these topics seem suitable, allow them to share examples of songs they could use and additional ideas they may have]:

1) Common physical and psychological symptoms of pregnancy and ways to cope such as:
- Importance of the singing group in supporting each other
- Importance of other positive relationships in their lives
- Resilience to challenges and empowerment
- Importance of being open, removing stigma to discuss challenges

2) Learning lullabies for after birth
3) How to care for the baby after birth

Session Structure

A typical session might involve:

- A welcome song at the start
- A middle section that would include one song that would focus on the topic of the week (see above), and others to promote a positive mood and encourage the women to move together.
- A closing song to draw the session to a close

How to run a successful session

Throughout the sessions we would like you to act as 'facilitators' rather than 'teachers'. We would like you to:

- Draw on your existing skills and ways of working to help us fulfill the goals described above.

- Encourage the women to be involved in whatever way they would like (please be aware the some women may not be comfortable to move very vigorously, and should feel encouraged to move in whatever way is comfortable). If someone is not joining in, check if they are okay, and try to engage them in a way that doesn't make them self-conscious.

- Notice how the women are responding to the activities that you initiate – is the pace too slow, too fast, too loud etc.? Try to adapt what you are doing according to the responses you see.

- Although it is fine to mention that pregnancy can involve challenges, it is important not to encourage the women to dwell on these, but instead to think about coping strategies and supporting each other, to ensure that the mood is a positive one.
