# Supplementary material for: Community psychosocial music intervention (CHIME) to reduce antenatal common mental disorder symptoms in The Gambia: a feasibility trial
Source: BMJ Open. 2020 Nov 23;10(11):e040287. doi: 10.1136/bmjopen-2020-040287 (PMC7684808; doi:10.1136/bmjopen-2020-040287)
Supplement: Supplementary data [file bmjopen-2020-040287supp002.pdf]

Supplementary Material 2

SRQ-20
Mandinka

The following questions are related to certain pains and problems that may have bothered you in the last 30 days. If you think the question applies to you and you have had the described problem in the last 30 days, answer YES.
On the other hand, if the question does not apply to you and you did not have the problem in the last 30 days, answer NO.
If you are unsure about how to answer a question, please give the best answer you can.

Nying nyininkari dantangolu i be dending dimoo doolu la aning mantooro doolu la meng y'a long a si ke a ye batandi nying tili tansabo meng tambita.
N'ii y'a mira ko nyininkaroo nying ye maa le, aning i ye nying mantooro sifa soto nying tili tansabo kono, i s'a fo haa.
N'ii y'a tara nyininkaroo nying m'ee maa, aning i mang nying mantooro sifa soto nying tili tansabo kono, i s'a fo haani.

1. Do you often have headaches?<br><br>1. Fo i ka kundimoo soto le waati ning waati?	☐1. Yes	☐0. No
2. Is you appetite poor?<br><br>2. Fo domoro buka diyaa i daa kono?	☐1. Yes	☐0. No
3. Do you sleep badly?<br><br>3. Fo ila siinoo buka beteyaa le bang?	☐1. Yes	☐0. No

4. Are you easily frightened?<br>4. Fo i ka tariyaa ka silla le bang?	☐1. Yes	☐0. No
5. Do your hands shake?<br>5. Fo i buloolu ka jarajara?	☐1. Yes	☐0. No
6. Do you feel nervous, tense or worried?<br>6. I ka fitii le bang wara sondomoo tenkung baliyaa k'e batandi le bang?	☐1. Yes	☐0. No
7. Is your digestion poor?<br>7. Fo n'ii ye domoro ke a buka tariyaa ka jii le bang?	☐1. Yes	☐0. No
8. Do you have trouble thinking clearly?<br>8. Fo miralotenkung baliyaa k'e batandi le bang?	☐1. Yes	☐0. No
9. Do you feel unhappy?<br>9. Fo i ka niitoroo soto le bang?	☐1. Yes	☐0. No

10. Do you cry more than usual?<br><br>10. Fo i ka kumboo danna tambiringo ke le bang?	☐1. Yes	☐0. No
11. Do you find it difficult to enjoy your daily activities?<br><br>11. Fo i buka kontan ka ila lung o lung haajoolu ke le bang?	☐1. Yes	☐0. No
12. Do you find it difficult to make decisions?<br><br>12. Fo I ka bataa kuwo soto le ka hajoolu t'ee i fango ye?	☐1. Yes	☐0. No
13. Is your daily work suffering?<br><br>13. I ka naasoo soto ila lung o lung dookuwo to bang?	☐1. Yes	☐0. No
14. Are you unable to play a useful part in life?<br><br>14. Fo i ka koleya kuwo soto ka nafaa kuwolu tamandi dunia baluwo kono?	☐1. Yes	☐0. No
15. Have you lost interest in things?<br><br>15. Fo ila lafoo talaata fengolu to i ka lafi meng na nung?	☐1. Yes	☐0. No

16. Do you feel that you are a worthless person?<br><br>16. Fo i k'e fango je ko i nafaa soto bang?	☐1. Yes	☐0. No
17. Has the thought of ending your life been on your mind?<br><br>17. Fo waati do ka soto i k'e mira pur ka i fango faa?	☐1. Yes	☐0. No
18. Do you feel tired all the time?<br><br>18. Fo i ka tara bataring waati o waati le bang?	☐1. Yes	☐0. No
19. Do you have uncomfortable feelings in your stomach?<br><br>19. Fo i kono k'e batandi le bang?	☐1. Yes	☐0. No
20. Are you easily tired?<br><br>20. Fo i ka tariyaa ka bataa?	☐1. Yes	☐0. No

SRQ-20
Wolof

The following questions are related to certain pains and problems that may have bothered you in the last 30 days. If you think the question applies to you and you have had the described problem in the last 30 days, answer YES.
On the other hand, if the question does not apply to you and you did not have the problem in the last 30 days, answer NO.
If you are unsure about how to answer a question, please give the best answer you can.

Lache yii daffa khetog meetit walla jeffeh-jeffeh yii nga khamneh manala sohnal si birr fenwerry-fen yu passeh. So fongehneh lache bi andana ak sa halat teh am nga meetit yii ak jeffeh-jeffeh yii si fenwerry-fen yu passeh, tontuma WAAW
Si benen borrbi, su lache bi andutak lee nga halat si yaw teh amulo jeffeh-jeffeh yii si birr fenwerry-fen yu passeh, tontma DADET
Su feekeh danga ham nyarry khel si negga warra tontor lache bi, ngela wyyy jokhma tontu bi nga fogneh mo ngane.

1. Do you often have headaches?<br><br>1. Ndakh dang de ham bopa bu meeti lego lake?	☐1. Yes	☐0. No
2. Is you appetite poor?<br><br>2. Ndakh sa leeka bi daffa wanyekou?	☐1. Yes	☐0. No
3. Do you sleep badly?<br><br>3. Ndakh dang de ham nelew bu bon?	☐1. Yes	☐0. No

4. Are you easily frightened?<br><br>4. Ndakh danga de gawa tit?	☐1. Yes	☐0. No
5. Do your hands shake?<br><br>5. Ndakh sa lohoyee daff de lokh?	☐1. Yes	☐0. No
6. Do you feel nervous, tense or worried?<br><br>6. Ndakh dang de yeek titangeh wala d jahleh?	☐1. Yes	☐0. No
7. Is your digestion poor?<br><br>7. Ndakh dang de ham leeka budut gawa ress?	☐1. Yes	☐0. No
8. Do you have trouble thinking clearly?<br><br>8. Ndakh dang de ham jeffeh-jeffeh si halat bu bakh?	☐1. Yes	☐0. No
9. Do you feel unhappy?<br><br>9. Ndakh dang de nyaka kontan?	☐1. Yes	☐0. No

10. Do you cry more than usual?<br><br>10. Ndakh dang de bug d joi lu barre?	☐1. Yes	☐0. No
11. Do you find it difficult to enjoy your daily activities?<br><br>11. Ndakh danga de ham jeffeh-jeffeh purr ham kontateh si leenga warra deff si bess bi?	☐1. Yes	☐0. No
12. Do you find it difficult to make decisions?<br><br>12. Ndakh dang de ham jeffeh-jeffeh purr deff saye halati boppa?	☐1. Yes	☐0. No
13. Is your daily work suffering?<br><br>13. Ndakh dor de ham khat-khat si sa leugue bess bi?	☐1. Yes	☐0. No
14. Are you unable to play a useful part in life?<br><br>14. Ndakh dang de ham jeffeh-jeffeh si lo hmneh dinala njering sisa ngerre dundu?	☐1. Yes	☐0. No
15. Have you lost interest in things?<br><br>15. Ndakh dor de nyaka eteh si lega warra deff	☐1. Yes	☐0. No

16. Do you feel that you are a worthless person?<br><br>16. Ndakh dang de ham yeek-yeek ne yaw dor darra?	☐1. Yes	☐0. No
17. Has the thought of ending your life been on your mind?<br><br>17. Ndakh danga de tog di halat neh dehh mo la ngenal?	☐1. Yes	☐0. No
18. Do you feel tired all the time?<br><br>18. Ndakh dang de soona wakhtu bu neeka?	☐1. Yes	☐0. No
19. Do you have uncomfortable feelings in your stomach?<br><br>19. Ndakh dang de ham yeek-yeek ngi jeffeh-jeffeh birr?	☐1. Yes	☐0. No
20. Are you easily tired?<br><br>20. Ndakh dang de gawa soona?	☐1. Yes	☐0. No

EPDS
Mandinka

Please tick the answer which comes closest to how you have felt *in the past week, not just how you feel today*. There are no right or wrong answers and please feel free to answer honestly as these questionnaires are confidential.

Bao saaying kono bee la, waranta i wuluuta a mang mee, m be lafi la ka a long i be nyaadi le. M be nyininkaroo ke la ka juubee i be nyaadii le tili worowulo nying kono, a manke i ye i fango je nyaameng bii damma.

1. Have you been able to laugh and see the funny side of things?<br><br>1. Fo i ka jele aning fo i ka jelekuwolu fanang kalamuta?	☐0.<br><br>As much as I always could<br><br>Haa, baake fango n lafita ala nyaameng	☐1.<br><br>Not quite so much now<br><br>A buka siyaa baake ko a be nung nyaameng	☐2.<br><br>Definitely not so much now<br><br>A buka siyaa fereng	☐3.<br><br>Not at all<br><br>M buka jele fereng
2. Have you looked forward with enjoyment to things?<br><br>2. Fo i ka niidiyaa kuwolu jamaa le nyaatosii?	☐0.<br><br>As much as I ever did<br><br>Haa, baake fango n lafita ala nyaameng	☐1.<br><br>Rather less than I used to<br><br>A buka siyaa baake ko a be nung nyaameng	☐2.<br><br>Definitely less than I used to<br><br>A buka siyaa fereng	☐3.<br><br>Hardly at all<br><br>Haani, m buka feng nyaatosii

3. Have you blamed yourself unnecessarily when things went wrong?<br><br>3. Fo i k'e fango jalai kensengke le ning kuwolu mang taa a nyaama?	☐3.<br><br>Yes, most of the time<br><br>Haa, waati jamaa	☐2.<br><br>Yes, some of the time<br><br>Haa, waati doolu	☐1.<br><br>Not very often<br><br>Haani, a mang siyaa	☐0.<br><br>No, never<br><br>Haani, mbuka wo ke fereng
4. Have you been anxious or worried for no good reason?<br><br>4. Fo i ka fiti le waranta i ka tara detering ne i fango feyi a ye a tara a mang jara wo la?	☐0.<br><br>No, not at all<br><br>Haani, m buka wo ke fereng	☐1.<br><br>Hardly ever<br><br>Haani, a mang siyaa	☐2.<br><br>Yes, sometimes<br><br>Haa, waati doolu	☐3.<br><br>Yes, very often<br><br>Haa, waati jamaa
5. Have you felt scared or panicky for no good reason?<br><br>5. Fo i ka silla wara i kijo k'e fara a y'a tara a mang jara wo la?	☐3.<br><br>Yes, quite a lot<br><br>Haa, waati jamaa	☐2.<br><br>Yes, sometimes<br><br>Haa, waati doolu	☐1.<br><br>No, not much<br><br>Haani, a buka siyaa	☐0.<br><br>No, not at all<br><br>Haani, mbuka wo ke

6. Have things been getting on top of you?<br><br>6. Hajoolu k'e detendi le fo a ka koleyaa i bulu ka ila kuwolu tamandi?	☐3.<br><br>Yes, most of the time I haven't been able to cope at all<br><br>Haa, jamaa jamaa a ka koleyaa m bulu	☐2.<br><br>Yes, sometimes I haven't been coping as well as usual<br><br>Haa, waati doolu a ka koleyaa	☐1.<br><br>No, most of the time I have coped quite well<br><br>Haani, jamaa jamaa a buka koleya m bulu	☐0.<br><br>No, I have been coping as well as ever<br><br>Haani, n ka nna kuwolu tamandi noo n ka ke nung nyaameng
7. Have you been so unhappy that you have had difficulty sleeping?<br><br>7. Fo kontaani baliyaa le ka tinna i buka siinoo noo a nyaama?	☐3.<br><br>Yes, most of the time<br><br>Haa, waati o waati	☐2.<br><br>Yes, sometimes<br><br>Haa, waati doolu	☐1.<br><br>Not very often<br><br>Haani, a buka siyaa	☐0.<br><br>No, not at all<br><br>Haani, wo mang soto
8. Have you felt sad or miserable?<br><br>8. Fo niikuyaa waranta niitooroo k'e batandi le bang?	☐3.<br><br>Yes, most of the time<br><br>Haa, waati o waati	☐2.<br><br>Yes, quite often<br><br>Haa, waati doolu	☐1.<br><br>Not very often<br><br>Haani, a buka siyaa	☐0.<br><br>No, not at all<br><br>Haani, wo buka m batandi

9. Have you been so unhappy that you have been crying?<br><br>9. Fo i ka tara kontaani baliyaa le kono fo i ka kumboo?	☐3.<br><br>Yes, most of the time<br><br>Haa, waati jamaa	☐2.<br><br>Yes, quite often<br><br>Haa, waati ning waati	☐1.<br><br>Only occasionally<br><br>Haa, bari a mang siyaa	☐0.<br><br>No, never<br><br>Haani, a mang soto
10. Has the thought of harming yourself occurred to you?<br><br>10. Fo waati doo ka soto i k'e mira pur ka kuu jawo ke i fang na?	☐3.<br><br>Yes, quite often<br><br>Haa, sinyaa jamaa	☐2.<br><br>Sometimes<br><br>Haa, waati doolu	☐1.<br><br>Hardly ever<br><br>Haani, a mang siyaa fereng	☐0.<br><br>Never<br><br>Haani, a mang soto

EPDS
Wolof

Please tick the answer which comes closest to how you have felt *in the past week,* not just how you feel today. There are no right or wrong answers and please feel free to answer honestly as these questionnaires are confidential.

Kom nu nga neeke geugeni birr ne wala musa ham doom, dange bugga ham sa yeek-yeek. Ndimbaleh nyu nga hol tontu bu ngena jageh si sa yeek-yeek si birr bess bu ai bu passeh. Kom neekut le ngai yeek tai.

1. Have you been able to laugh and see the funny side of things?<br><br>1. Ndakh yaw dang nga deh muna rreh sor gisseh lula rretanlo?	☐0.<br><br>As much as I always could<br><br>Waaw, lu si baari kom nu ma ko muneh	☐1.<br><br>Not quite so much now<br><br>Waaw, neekut lu bareh kom nonu leegue	☐2.<br><br>Definitely not so much now<br><br>Degga-degga neekut lu bareh sah leegue	☐3.<br><br>Not at all<br><br>Amut sah
2. Have you looked forward with enjoyment to things?<br><br>2. Ndakh yaw dang deh muna saintu yo hamneh munnala kontanlo?	☐0.<br><br>As much as I ever did<br><br>Waaw, kom numa ko musa deffeh	☐1.<br><br>Rather less than I used to<br><br>Waaw, lu meelut kom nu ma ko dan deffeh	☐2.<br><br>Definitely less than I used to<br><br>Degga-degga neekut kom numa kodan deffeh	☐3.<br><br>Hardly at all<br><br>Waaw, su ameh sah barewut

3. Have you blamed yourself unnecessarily when things went wrong?<br><br>3. Ndakh yaw dang deh am sikka nge si sa boppa su mbirr dameh nom waruta demeh?	☐3.<br><br>Yes, most of the time<br><br>Waaw, si wakhtu yu bareh	☐2.<br><br>Yes, some of the time<br><br>Waaw, si yeena sayee	☐1.<br><br>Not very often<br><br>Neekut lu bareh	☐0.<br><br>No, never<br><br>Dadet, musuta ham
4. Have you been anxious or worried for no good reason?<br><br>4. Ndakh dang de ham jahleh walla hel bu dalut si li ko jarut?	☐0.<br><br>No, not at all<br><br>Dadet musuta ham	☐1.<br><br>Hardly ever<br><br>Su ameh yet bareh wut	☐2.<br><br>Yes, sometimes<br><br>Waaw, yeena sayee	☐3.<br><br>Yes, very often<br><br>Waaw, si wahktu yu bareh
5. Have you felt scared or panicky for no good reason?<br><br>5. Ndakh danga de ham ragal walla titangeh si lu ko jarut?	☐3.<br><br>Yes, quite a lot<br><br>Waaw, si wakhtu yu bareh	☐2.<br><br>Yes, sometimes<br><br>Waaw, yeena sayee	☐1.<br><br>No, not much<br><br>Dadet, barehwut	☐0.<br><br>No, not at all<br><br>Dadet, musuta ham sah

6. Have things been getting on top of you?<br><br>6. Ndakh Jeffeh-jeffeh daffa bareh si sa boopa bi be-nga hamneh anta nulo ko?	☐3.<br><br>Yes, most of the time I haven't been able to cope at all<br><br>Waaw, yeena sayee duma henu jeffeh-jeffeh yoyu	☐2.<br><br>Yes, sometimes I haven't been coping as well as usual<br><br>Waaw, yeena sayee duma deh muna henu jeffeh-jeffeh yoyu kom num wara demeh	☐1.<br><br>No, most of the time I have coped quite well<br><br>Dadet, yeena sayee dama deh muna henu jeffeh- jeffeh yoyu	☐0.<br><br>No, I have been coping as well as ever<br><br>Dadet, dama deh henu jeffeh-jeffeh yoyu kom nom musa mel
7. Have you been so unhappy that you have had difficulty sleeping?<br><br>7. Ndakh danga de ham nyaka kontante (walla nakhar) be takh dor deh muna nelew?	☐3.<br><br>Yes, most of the time<br><br>Waaw, wakhtu bu neeka	☐2.<br><br>Yes, sometimes<br><br>Waaw, yeena sayee	☐1.<br><br>Not very often<br><br>Neekut lu bareh	☐0.<br><br>No, not at all<br><br>Dadet, musuta ham sah
8. Have you felt sad or miserable?<br><br>8. Ndakh danga de yeek nakhar walla chono bo hamneh daffa doi war?	☐3.<br><br>Yes, most of the time<br><br>Waaw, wakhtu yu bareh	☐2.<br><br>Yes, quite often<br><br>Waaw, yeena sayee	☐1.<br><br>Not very often<br><br>Neekut lu bareh	☐0.<br><br>No, not at all<br><br>Dadet, musuta ham sah

9. Have you been so unhappy that you have been crying?<br><br>9. Ndakh dang de ham nekhar bula deh joi lo?	☐3.<br><br>Yes, most of the time<br><br>Waaw, wakhtu yu bareh	☐2.<br><br>Yes, quite often<br><br>Waaw, yeena sayee	☐1.<br><br>Only occasionally<br><br>Lu tuti si ai hew-hew	☐0.<br><br>No, never<br><br>Dadet, musuta ham
10. Has the thought of harming yourself occurred to you?<br><br>10. Ndakh am halat purr lurr sa boopa daff deh nyaw sisa hell bi?	☐3.<br><br>Yes, quite often<br><br>Waaw, si wahktu yu bareh	☐2.<br><br>Sometimes<br><br>Yeena sayee	☐1.<br><br>Hardly ever<br><br>Suma amet het barewut	☐0.<br><br>Never<br><br>Musuta ham
