# Supplementary material for: Community psychosocial music intervention (CHIME) to reduce antenatal common mental disorder symptoms in The Gambia: a feasibility trial
Source: BMJ Open. 2020 Nov 23;10(11):e040287. doi: 10.1136/bmjopen-2020-040287 (PMC7684808; doi:10.1136/bmjopen-2020-040287)
Supplement: Supplementary data [file bmjopen-2020-040287supp003.pdf]

Supplementary Material 3

CHIME session Check-List

1. Did any of the songs address any of the following topics? Y/N (Circle which ones below)
    a. Importance of the singing group in supporting each other
    b. Importance of other positive relationships in their lives
    c. Resilience to challenges and empowerment
    d. Importance of being open, removing stigma to discuss challenges

2. Did the sessions dwell on the challenges of pregnancy or more the coping mechanisms and resilience? Y/N

3. Did the kanyeleng group incorporate a lullaby? If so, which? Y/N
    a. Lullaby name:
4. Did the kanyeleng group explain the benefits of singing lullabies? Y/N

5. Did the kanyeleng group use a welcoming song? If so, which? Y/N
    a. Song name:

6. Did the kanyeleng group use a closing song at the end? If so, which song? Y/N
    a. Song name:

7. Overall, did the participants actively participate in the session by joining in with the singing, dancing or clapping? Y/N

8. Were there any examples where the kanyeleng group acted to encourage women to participate? Y/N
    a. Give example and time point in the video:
