# Supplementary material for: Community psychosocial music intervention (CHIME) to reduce antenatal common mental disorder symptoms in The Gambia: a feasibility trial
Source: BMJ Open. 2020 Nov 23;10(11):e040287. doi: 10.1136/bmjopen-2020-040287 (PMC7684808; doi:10.1136/bmjopen-2020-040287)
Supplement: Supplementary data [file bmjopen-2020-040287supp004.pdf]

Supplementary Material 4

Retention and Attrition Rates Overall and by Group

	**Retention**			**Attrition**		
	**Pre** ***n*** **(%)**	**Post** ***n*** **(%)**	**Follow-up** ***n*** **(%)**	**Pre-Post (%)**	**Post-Follow-up (%)**	**Pre-Follow-up (%)**
**All**	124 (100%)	99 (80%)	83 (67%)	20%	16%	33%
**Intervention**	50 (100%)	39 (78%)	33 (66%)	22%	15%	34%
**Control**	74 (100%)	60 (81%)	50 (68%)	19%	17%	32%
