# Supplementary material for: Community psychosocial music intervention (CHIME) to reduce antenatal common mental disorder symptoms in The Gambia: a feasibility trial
Source: BMJ Open. 2020 Nov 23;10(11):e040287. doi: 10.1136/bmjopen-2020-040287 (PMC7684808; doi:10.1136/bmjopen-2020-040287)
Supplement: Supplementary data [file bmjopen-2020-040287supp005.pdf]

Supplementary Material 5

CHIME Session Duration

Site	Session 1	Session 2	Session 3	Session 4	Session 5	Session 6	Average
	**Session Duration**						
Gunjur	31.00	45.00	48.00	60.00	57.00	50.00	48.50
Kuntair	42.00	75.00	75.00	70.00	66.00	71.00	66.50
Pirang	75.00	50.00	65.00	55.00	50.00	65.00	60.00
Sukuta	55.00	78.00	64.00	61.00	60.00	60.00	63.00
**Total**	**50.75**	**62.00**	**63.00**	**61.50**	**58.25**	**61.50**	**59.50**

Note. Time is in minutes.

Number of participants Present at Each Session

Site	Total Possible	Session 1	Session 2	Session 3	Session 4	Session 5	Session 6
	**Session Attendance**						
Gunjur	11	9	6	7	8	7	8
Kuntair	11	11	10	8	11	11	11
Pirang	16	13	12	11	10	12	11
Sukuta	13	10	5	7	4	4	6

Note. Total Possible is the total amount of women recruited to be in the intervention.
