# Supplementary material for: Community psychosocial music intervention (CHIME) to reduce antenatal common mental disorder symptoms in The Gambia: a feasibility trial
Source: BMJ Open. 2020 Nov 23;10(11):e040287. doi: 10.1136/bmjopen-2020-040287 (PMC7684808; doi:10.1136/bmjopen-2020-040287)
Supplement: Supplementary data [file bmjopen-2020-040287supp006.pdf]

Supplementary Material 6

EPDS and SRQ-20 Scores at Three Time-Points

**EPDS**		**Baseline**		**Post**		**Follow-up**
	n	***M(SD)***	***n***	***M(SD)***	***n***	***M(SD)***
All	124	4.25 (4.10)	99	2.56 (2.74)	83	1.80 (2.23)
Intervention	50	2.90 (3.07)	39	1.18 (1.50)	33	0.94 (1.73)
Control	74	5.16 (4.46)	60	3.45 (2.99)	50	2.36 (2.36)

**SRQ-20**		**Baseline**		**Post**		**Follow-up**
	n	***M(SD)***	***n***	***M(SD)***	***n***	***M(SD)***
All	124	7.27 (4.00)	99	5.31 (4.02)	83	4.25 (3.32)
Intervention	50	6.22 (3.83)	39	3.62 (3.32)	33	2.70 (3.03)
Control	74	7.97 (3.99)	60	6.42 (4.08)	50	5.28 (3.12)

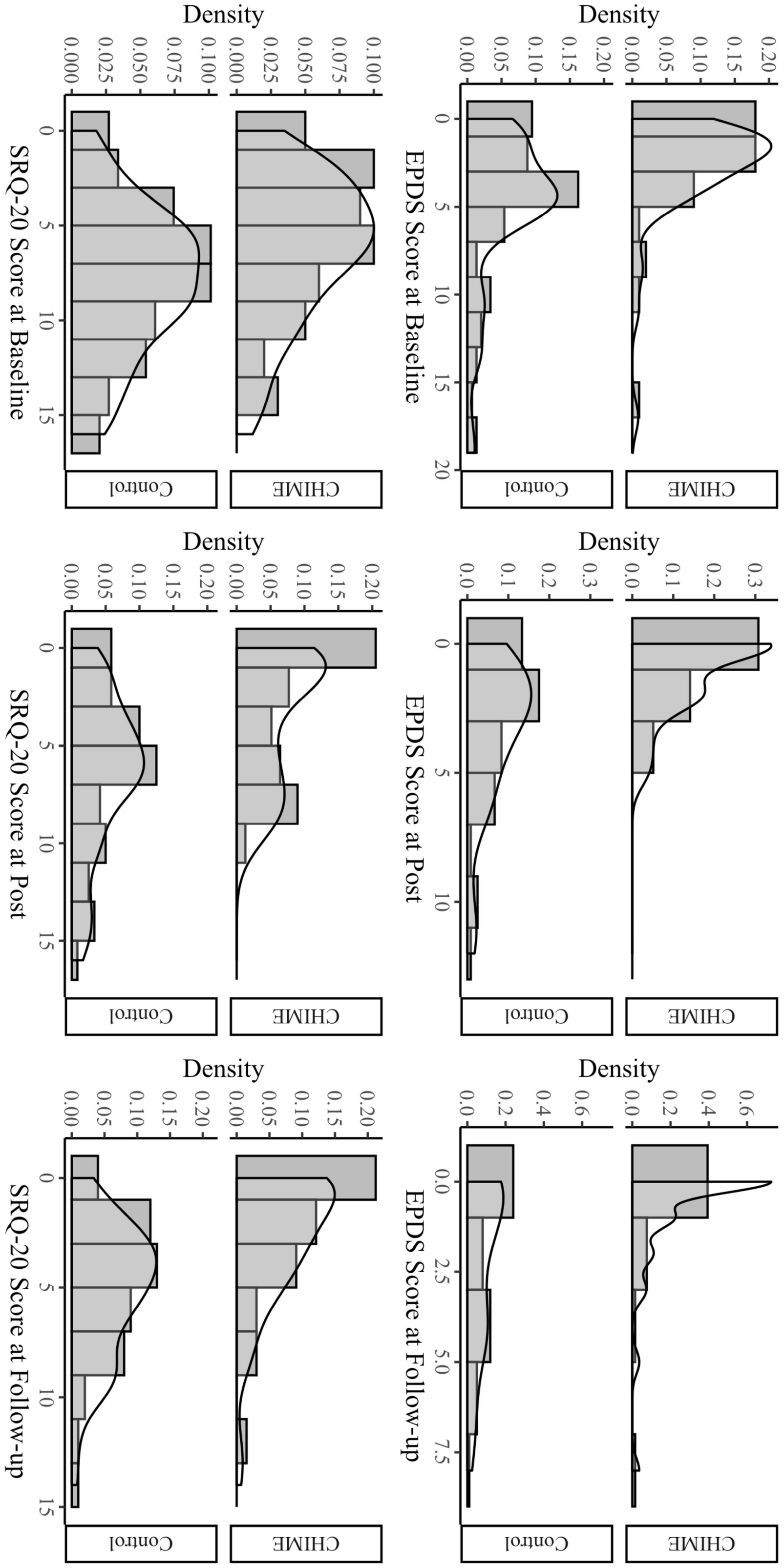
Density
SRQ-20 Score at Baseline
EPDS Score at Baseline
SRQ-20 Score at Post
EPDS Score at Post
SRQ-20 Score at Follow-up
EPDS Score at Follow-up
Control
CHIME
