# Supplementary material for: Community psychosocial music intervention (CHIME) to reduce antenatal common mental disorder symptoms in The Gambia: a feasibility trial
Source: BMJ Open. 2020 Nov 23;10(11):e040287. doi: 10.1136/bmjopen-2020-040287 (PMC7684808; doi:10.1136/bmjopen-2020-040287)
Supplement: Supplementary data [file bmjopen-2020-040287supp007.pdf]

Supplementary Material 7

Mixed Model Results

	Post intervention		Post intervention + 4 weeks follow up	
	**adjusted mean difference (95%CI)**	**p-value**	**adjusted mean difference (95%CI)**	**p-value**
**SRQ-20**	2.13 (0.89, 3.38)	<0.01	2.09 (0.76, 3.42)	<0.01
**EPDS**	1.98 (1.06, 2.90)	<0.01	0.98 (-0.01, 1.97)	0.05

Note. Mixed models adjusted for baseline score and clinic; MD>0 favour intervention arm
