# Supplementary material for: Community psychosocial music intervention (CHIME) to reduce antenatal common mental disorder symptoms in The Gambia: a feasibility trial
Source: BMJ Open. 2020 Nov 23;10(11):e040287. doi: 10.1136/bmjopen-2020-040287 (PMC7684808; doi:10.1136/bmjopen-2020-040287)
Supplement: Author's manuscript [file bmjopen-2020-040287.draft_revisions.pdf]

BMJ Open

BMJ Open is committed to open peer review. As part of this commitment we make the peer review history of every article we publish publicly available.

When an article is published we post the peer reviewers' comments and the authors' responses online. We also post the versions of the paper that were used during peer review. These are the versions that the peer review comments apply to.

The versions of the paper that follow are the versions that were submitted during the peer review process. They are not the versions of record or the final published versions. They should not be cited or distributed as the published version of this manuscript.

BMJ Open is an open access journal and the full, final, typeset and author-corrected version of record of the manuscript is available on our site with no access controls, subscription charges or pay-per-view fees (http://bmjopen.bmj.com).

If you have any questions on BMJ Open's open peer review process please email info.bmjopen@bmj.com

BMJ Open

A community psycho-social music intervention to reduce antenatal common mental disorder symptoms in The Gambia: A feasibility study

Journal:	*BMJ Open*
Manuscript ID	bmjopen-2020-040287
Article Type:	Original research
Date Submitted by the Author:	09-May-2020
Complete List of Authors:	Sanfilippo, Katie Rose; Goldsmiths University of London, Psychology<br>McConnell, Bonnie ; Australian National University<br>Cornelius, Victoria; Imperial College London, Clinical Trials Unit<br>Darboe, Buba; Ministry of Health and Social Welfare The Gambia<br>Huma, Hajara; Ministry of Health and Social Welfare The Gambia; National Centre for Arts and Culture The Gambia<br>Gaye, Malick ; Ministry of Health and Social Welfare The Gambia; National Centre for Arts and Culture The Gambia<br>Ceesay, Hassoum ; National Centre for Arts and Culture<br>Ramchandani, Paul; University of Cambridge<br>Glover, Vivette; Imperial College London<br>Cross, Ian; Cambridge University<br>Stewart, Lauren ; Goldsmiths University of London
Keywords:	Maternal medicine < OBSTETRICS, Anxiety disorders < PSYCHIATRY, Depression & mood disorders < PSYCHIATRY, PUBLIC HEALTH

SCHOLARONE™ Manuscripts

I, the Submitting Author has the right to grant and does grant on behalf of all authors of the Work (as defined in the below author licence), an exclusive licence and/or a non-exclusive licence for contributions from authors who are: i) UK Crown employees; ii) where BMJ has agreed a CC-BY licence shall apply, and/or iii) in accordance with the terms applicable for US Federal Government officers or employees acting as part of their official duties; on a worldwide, perpetual, irrevocable, royalty-free basis to BMJ Publishing Group Ltd ("BMJ") its licensees and where the relevant Journal is co-owned by BMJ to the co-owners of the Journal, to publish the Work in this journal and any other BMJ products and to exploit all rights, as set out in our licence.

The Submitting Author accepts and understands that any supply made under these terms is made by BMJ to the Submitting Author unless you are acting as an employee on behalf of your employer or a postgraduate student of an affiliated institution which is paying any applicable article publishing charge ("APC") for Open Access articles. Where the Submitting Author wishes to make the Work available on an Open Access basis (and intends to pay the relevant APC), the terms of reuse of such Open Access shall be governed by a Creative Commons licence – details of these licences and which Creative Commons licence will apply to this Work are set out in our licence referred to above.

Other than as permitted in any relevant BMJ Author's Self Archiving Policies, I confirm this Work has not been accepted for publication elsewhere, is not being considered for publication elsewhere and does not duplicate material already published. I confirm all authors consent to publication of this Work and authorise the granting of this licence.

For peer review only

A community psycho-social music intervention to reduce antenatal common mental disorder symptoms in The Gambia: A feasibility study

Katie Rose M. Sanfilippo[*1], Bonnie B. McConnell[#2], Victoria Cornelius[†3], Buba Darboe[⊥4], Hajara B. Huma[§⊥5], Malick Gaye [§⊥6], Paul Ramchandani[‡7], Hassoum Ceesay[§8], Ian Cross[‡9], Vivette Glover[†10], Lauren Stewart[*11]

[*] *Psychology Department, Goldsmiths, University of London, UK*

[#] *The Australian National University, Australia*

[†] *Imperial College London, UK*

[‡] *University of Cambridge, UK*

[§] *The National Centre for Arts and Culture, The Gambia*

[⊥] *The Ministry of Health and Social Welfare, The Gambia*

[1]ksanf001@gold.ac.uk, [2]Bree.McConnell@anu.edu.au, [3]v.cornelius@imperial.ac.uk, [4]darboejula@hotmail.com, [5]hajarahuma24@gmail.com, [6]malickgaye60@yahoo.com, [7]hceesay@gmail.com, [8]pr441@cam.ac.uk, [9]v.glover@imperial.ac.uk, [10]ic108@cam.ac.uk, [11]l.stewart@gold.ac.uk

Correspondence concerning this paper should be addressed to Katie Rose M. Sanfilippo

Address: Whitehead Building, Psychology Department, Goldsmiths, University of London, SE14 6NW, UK.

E-mail: ksanf001@gold.ac.uk

Word count: 4,565

For peer review only - http://bmjopen.bmj.com/site/about/guidelines.xhtml

ABSTRACT

Objectives: Examine the feasibility of a Community Health Intervention through Musical Engagement (CHIME) in The Gambia to reduce common mental disorder (CMD) symptoms in pregnant women.

Design: Feasibility trial testing a randomised stepped wedge cluster design.

Setting: Four local antenatal clinics.

Participants Women who were 14 – 24 weeks pregnant and spoke Mandinka or Wolof were recruited into the CHIME (n = 50) or control group (n = 74).

Intervention: The intervention included six weekly hour-long sessions, was co-developed with and led by local all-female fertility societies, and was delivered to groups of women with no preselection, including women with a range of CMD symptoms. Sessions were designed to lift mood, build social connection and provide health messaging through participatory music making. The control group received standard antenatal care.

Outcomes: Demographic, feasibility, acceptability outcomes, and the appropriateness of the study design were assessed. Translated measurement tools (Self-Reporting Questionnaire (SRQ-20); Edinburgh Postnatal Depression Scale (EPDS)) were used to assess CMD symptoms at baseline, post-intervention and 4-weeks follow-up.

Results: All clinics and 82% of women approached consented to take part. A 33% attrition rate across all time-points was observed. 72% in the CHIME group attended at least three sessions. Audio and video analysis confirmed fidelity of the intervention and a thematic analysis of participant interviews demonstrated acceptability and positive evaluation. Results showed a potential beneficial effect with a reduction of 2.13 points

For peer review only - http://bmjopen.bmj.com/site/about/guidelines.xhtml

(95%CI [0.89, 3.38], $p < 0.01$, $n = 99$) on the SRQ-20 and 1.98 points (95% CI [1.06, 2.90], $p < 0.01$, $n = 99$) on the EPDS for the intervention group compared to standard care.

Conclusion: Results demonstrate that CHIME is acceptable and feasible in The Gambia. This project is the first to highlight how music engagement can help improve women's perinatal mental health in a LMIC.

Trial Registration: Pan African Clinical Trials Registry (PACTR) (24/01/2019): PACTR201901917619299

For peer review only

ARTICLE SUMMARY

Strengths and limitations of the study

- This feasibility study is the first to examine how a psycho-social intervention delivered through participatory music making may help improve women's perinatal mental health in a low-income country.
- The intervention is context specific and co-developed with all female fertility societies (Kanyeleng groups) who already perform ceremonies and provide health messaging for women around reproductive health.
- It is a population level intervention requiring no screening for use.
- Participants were unable to be blinded to which group they were assigned, increasing the potential for response bias.

For peer review only - http://bmjopen.bmj.com/site/about/guidelines.xhtml

- The low-cost and embedded nature of the intervention as well as the involvement of project partners at the Ministry of Health & Social Welfare provide high potential for sustainability.

BACKGROUND

Perinatal common mental health disorders (CMDs) affect up to one in five women worldwide and in low- and middle-income countries (LMICs) they can be twice as frequent as in high income countries (HICs) [1]. The World Health Organization (WHO) International Classification of Disease (ICD-10) defines CMDs as “mood disorders’ and ‘neurotic, stress-related and somato-form disorders” which include depressive and anxiety disorders [2]. Perinatal mental distress is of global concern as mental health problems such as stress, anxiety and depression have been shown to have a negative impact on the mother, her developing infant, and their relationship [3–5]. Antenatal anxiety and depression strongly predict postnatal depression [6,7] and can negatively impact fetal and infant development [8,9]. Hence, antenatal mental health interventions are important in order to improve outcomes for both the mother and child. In LMICs, there is often a scarcity of psychologists and psychiatrists able to treat affected women. Therefore, it is necessary to develop effective, low-cost, non-stigmatising and culturally appropriate interventions to support women's mental health during the perinatal period [10–12].

The majority of previous research in community mental health and perinatal mental health interventions in LMICs focus on educational and psychosocial interventions

For peer review only - http://bmjopen.bmj.com/site/about/guidelines.xhtml

primarily delivered via conversations and social gatherings. The possibility of using participatory music making as the vehicle to deliver a perinatal mental health intervention has not been studied within a LMIC context. Research in HICs has shown that singing in groups can be a powerful modulator of mood and emotion, reducing symptoms of anxiety and depression [13], including during pregnancy [14–16], and increasing well-being and social affiliation, group bonding and lift participants' mood [17–19]. In LMICs, songs have shown to be a helpful and common medium to convey specific health messages, especially in areas where literacy rates are low [20–22]. These principles formed the basis for the developed intervention.

The current project took place within The Gambia, a small country on the West African coast. With a population of about two million, The Gambia is the most densely populated country in West Africa [23]. There are five major ethnic groups in The Gambia, each with their own language and culture. Mandinka is the most common (42%) followed by Fula (18%), Wolof (16%), Jola (10%), Serahuli (9%) and smaller ethnic groups and foreigners comprising the remainder [24]. A large majority of households (91%) have access to an improved source of drinking water, and 45% of households have access to electricity, however, there is a large disparity between urban and rural areas (66% and 13%, respectively) [25]. Only 37% of households use improved toilet facilities that hygienically separate human excreta from human contact and are not shared with other households [25]. The World Bank estimates a $31 per capita health expenditure per annum, one of the lowest in the world [26]. Mental health services and specifically services focused on perinatal mental health are minimal or non-existent [25]. There are

For peer review only - http://bmjopen.bmj.com/site/about/guidelines.xhtml

also high levels of stigma associated with CMDs that impede women's ability to recognise symptoms and seek help and support.

The Gambia is a medically pluralistic society where people utilize a variety of therapeutic options, including biomedicine, indigenous herbal medicines, and spiritual treatments [27]. A music-centred approach for perinatal mental health may be particularly fruitful in The Gambia as there are already many different existing music practices that involve health communication, pregnant women and new mothers. Kanyeleng groups, or fertility societies, sing together and perform ceremonies that promote fertility and support women during pregnancy and throughout motherhood [28]. They also work with the Ministry of Health and Social Welfare to promote health initiatives through performance [20]. The intervention examined here was co-developed with local Kanyeleng groups with the aim of having women leave the sessions in a positive mood, feeling part of a socially-supportive group, with some new strategies of how to cope with some of the common physical and psychological challenges of pregnancy and equipped with skills to seek wider social support outside the sessions. To our knowledge this is the first study to investigate the impact of participatory music making for perinatal mental health in a LIMC context.

Objectives

The main objective of this study was to test the feasibility of a Community Health Intervention through Musical Engagement (CHIME) to help reduce CMD symptoms in pregnant women compared with standard care. The study had five objectives:

For peer review only - http://bmjopen.bmj.com/site/about/guidelines.xhtml

1. To obtain demographic information on the eligible population
2. To determine if our measurement tools, the Edinburgh Postnatal Depression Scale (EPDS) and the Self-Reporting Questionnaire (SRQ-20), are useable.
3. To determine if the intervention is deliverable.
4. To determine if the stepped wedge trial design is deliverable and obtain information that will inform the definitive study.
5. To determine if this type of intervention is culturally appropriate and well received by the community and health workers.

METHODS

The trial protocol has been published and contains more detailed information about the intervention development and methods [29].

Trial design

The randomised stepped wedge cluster trial design had two steps with 4 clusters (antenatal clinics). All clinics undertook the control group period prior to the intervention (CHIME) group period. The two clinics randomised to sequence 1 undertook these immediately, the two clinics randomised to sequence 2 undertook their control groups 12 weeks later. Randomisation was undertaken after clinic consent was given to take part in the trial. The study statistical advisor generated a computer randomisation list with a block size of 2, which was applied to the pre-supplied clinic list by the trial team. Separate cohorts of participants were recruited for each period to the control group and the intervention (CHIME) group, ensuring all participants began the study 4-6 months

For peer review only - http://bmjopen.bmj.com/site/about/guidelines.xhtml

through their pregnancy. The 12-week phase for both the control and intervention group included data collection at week 1 ('baseline') and week 7 ('post-intervention') after either participating in the music sessions (CHIME) or standard care (Control). Data was also collected at week 11 ('follow-up'), four weeks after the intervention finished. This study is reported in accordance with the Consolidated Standards of Reporting Trials 2010 statement: extension to randomised pilot and feasibility trials [30].

Clinic inclusion

Sites were in The Gambia, spanned a range of locations and ethnic groups, including rural and urban as well as Wolof- and Mandinka-speaking areas. Each clinic also had an active local Kanyeleng group that could deliver the intervention.

Participants

Participants were not preselected based on their mental health symptoms. All participants who were attending the consented sites during the active study period and who were 18 or older, spoke either Mandinka or Wolof fluently, and were 14 – 24 weeks pregnant were invited to take part in the study. Women with a history of a previous late-term miscarriage or those who had current or a history of psychosis were excluded. Other ethical considerations are given within the protocol paper [29].

Recruitment

Participants were recruited during antenatal clinic days. All data was collected at the antenatal clinics. The researchers and participants were not blinded to whether they were

For peer review only

in the intervention or control group. All participants were offered a total of 600 Dalasi for their time, 200 at each data collection time point. They were reminded of the data collection and the intervention sessions by a phone call. They were called three days before, one day before and on the day of data collection.

Sample Size

The size of the study was based on its ability to inform the design of a future definitive study. We aimed to collect data from 120 pregnant women, 60 controls and 60 in the intervention. This number was sufficient to provide an estimate of binary feasibility outcome within at least ±9 percentage points for the 95% confidence interval and provide reasonable estimates of the standard deviation [31]. As the intervention delivery was thought to be possible within groups of approximate 15, the aim was to include 4 clinics with one control and one intervention group per clinic.

For peer review only

Intervention

The intervention was psycho-social and musical in nature, with sessions centring on group singing and led by the Kanyeleng group local to each antenatal clinic. Each Kanyeleng group is comprised of approximately 10 women. The nature of the intervention was necessarily contextualized across the four settings, especially as Wolof- and Mandinka-speaking groups have distinct cultural beliefs, practices and language. A training workshop held with the Kanyeleng groups before the intervention began ensured that the overarching goals, content and approach to session delivery could be broadly standardized. Key messages incorporated within the session songs included: a) common

For peer review only - http://bmjopen.bmj.com/site/about/guidelines.xhtml

physical and psychological symptoms of pregnancy, b) techniques to cope with and manage these with an emphasis on the importance of the singing group in supporting each other, other positive relationships in their lives, resilience to challenges and empowerment, and the importance of being opening and removing stigma to discuss challenges, and c) select messages on childcare. The document used to aid the discussions during the intervention development workshops can be found in Supplementary Material 1.

Participants in the CHIME intervention attended six 60-minute music sessions held at their local antenatal clinic in addition to receiving standard care. During each session, the Kanyeleng groups introduced specific songs from the traditional repertoire and those created during the workshops focused on the key messages. The participants were encouraged to join in by singing, moving to the music and clapping. Sessions involved call-and-response singing, with participants improvising along the themes described above introduced by the Kanyeleng group. Each session began with a welcome song and ended with a closing song. One lullaby was also introduced at each session to give the women repertoire to draw on postpartum. A community health nurse (CHN) at each clinic was present to observe, take attendance data, and report any issues of concern to the research team including any potential adverse effects.

Each control group received standard antenatal clinic care without any additional intervention. Standard care consists of 4 or more regular visits to the antenatal clinic with little to no mental health care [25].

For peer review only - http://bmjopen.bmj.com/site/about/guidelines.xhtml

207

208 **Outcomes**

209 Two questionnaires were used to measure CMD symptoms. Both were translated into
210 Mandinka and Wolof. The translation method was based on suggestions from the WHO
211 [32], Hanlon et al. [33] and Cox, Holden and Henshaw [34]. The translated versions can
212 be found in Supplementary Materials 2.

213

214 The Self-report Questionnaire (SRQ-20) [35] is a 20-item scale developed by the WHO
215 to screen for psychiatric disturbance, especially in LMICs. Its items ask about anxiety,
216 depression and somatoform symptoms. It has been used in various sub-Saharan African
217 contexts to measure perinatal mental health [36] but has never been used in The Gambia.

218

219 The second measurement tool was the Edinburgh Postnatal Depression Scale (EPDS), a
220 ten-item scale that was developed to screen for postnatal depression [34] and has
221 subsequently been validated to be used during pregnancy [37]. It has been used and
222 validated in other African contexts [36,38], and used in The Gambia before, though a
223 validated version could not be obtained [39–41].

224

225 **Procedure**

226 Four antenatal clinics in western Gambia were approached to participate in the feasibility
227 trial. The head midwife gave consent via a paper consent form. Participants who met the
228 inclusion criteria were read the information sheet and consent was given orally and

For peer review only

verified via thumbprint or signature. All data were collected orally and recorded on paper Case Report Forms (CRFs).

At all three assessments (baseline, post-intervention, follow-up) EPDS and SRQ-20 scores were collected. Demographic data were collected at baseline and a semi-structured interview was held with each participant within the CHIME group at the post-intervention assessment. Focus group discussions (FGDs) were conducted with each of the four Kanyeleng groups and the CHNs. The questions in the interviews and FGDs addressed the participants' overall thoughts about the intervention, whether or not they thought it had been helpful, their comments on the structure, and any suggestions for the future. All interviews and FGDs were audio-recorded, transcribed and translated.

Patient and Public Involvement

We engaged patients and the public to inform the design of this trial, intervention development, acceptability and dissemination strategy. We engaged in extensive involvement with relevant stakeholders to ensure optimal study design and acceptability of the intervention including a half day meeting in the Gambia, with delegates from the Ministry of Health and Social Welfare, the National Centre for Arts and Culture, a local obstetrician, patient advocate groups and students in Public Health and Psychiatric Nursing. The dissemination of these findings will include open field days in the local community (mini festivals with speeches and musical performances) allowing us to share findings and obtain feedback from the community.

For peer review only - http://bmjopen.bmj.com/site/about/guidelines.xhtml

Statistical Methods

Analysis was conducted using the intention-to-treat principle [42]. All eligible participants were analysed in the group to which they were randomized regardless of adherence to the intervention.

Descriptive statistics were used to summarise demographic variables of the recruited population. Plots were used, and skewness calculated, to assess the distribution of the outcome measurements across the three assessment time-points and by each group. To determine if the intervention was deliverable, the number of sessions the Kanyeleng delivered, the duration of each session, and intervention fidelity at the four sites was recorded. To measure the fidelity of the intervention, both RAs watched audio-visual recordings of the first and fourth intervention sessions from each clinic. They then completed a checklist (Supplementary Materials 3) to determine if all the necessary elements, as outlined in the training workshops, had been included in the intervention. Proportions of specific agreement were calculated to assess the reliability of the fidelity measure. The proportion of approached clinics that gave consent was calculated, and any scheduling problems in keeping with the stepped-wedge timeline were recorded. Recruitment and adherence rates were calculated for both groups.

The intervention effect was estimated using mixed effect linear regression models. Two regression models were created to estimate the between-group mean difference in SRQ-20 and EPDS score 1 week after completion of the intervention (post-intervention) and 4 weeks later (follow-up). Each model was adjusted for baseline scores, clinic and included

For peer review only

For peer review only - http://bmjopen.bmj.com/site/about/guidelines.xhtml

a time by group interaction with participant as a random effect and unstructured covariance. The mean differences were presented with 95% confidence intervals.

For missing values on the SRQ-20 or EPDS individual items, multiple imputation was performed using a predictive mean matching method using the *MICE* package in R [43]. The model included all quantitative demographic information collected, including age, gestational age, parity and gravida. If a participant's data was not able to be collected at one of the post-intervention assessments (post-intervention or follow-up) due to attrition it was treated as missing.

All statistical analysis was run using R [44]. A thematic analysis [45] of the post-intervention interviews and FGDs with the participants, Kanyeleng groups and CHNs was performed using Dedoose [46].

RESULTS

Demographic information

Table 1 shows the demographic information of the entire sample and by group. Participants were between the ages of 18 and 40 (M = 26.95, SD = 5.72) and between 14 and 24 weeks pregnant (M = 20.81, SD = 3.32). It can be seen in Table 1 that the distribution of the participant characteristics are similar between the two groups.

Table 1. Demographic data in total and by group

For peer review only - http://bmjopen.bmj.com/site/about/guidelines.xhtml

	All Women (*n* = 124) *M*(SD)	CHIME Group (*n*= 50) *M*(SD)	Control Group (*n*= 74) *M*(SD)
**Age**	26.95 (5.72)	26.82 (5.59)	27.04 (5.85)
**Gestational Age**	20.81 (3.32)	21.14 (3.26)	20.58 (3.36)
**Gravida**	3.68 (2.19)	3.74 (2.32)	3.65 (2.11)
	***n*(% of 124)**	***n*(% of 50)**	***n*(% of 74)**
**Parity**			
Primiparous	27 (22%)	11 (22%)	16 (22%)
Multiparous	97 (78%)	39 (78%)	58 (78%)
**Marital Status**			
Single/Divorced/Separated/Widowed	5 (4%)	1 (2%)	4 (5%)
Married (Monogamous)	83 (67%)	37 (74%)	46 (62%)
Married (Polygamous)	36 (29%)	12 (24%)	24 (33%)
**Education Level**			
None	5 (4%)	3 (6%)	2 (3%)
Informal (Arabic)	62 (50%)	28 (56%)	34 (46%)
Primary	19 (15%)	7 (14%)	12 (16%)
Secondary/Tertiary	38 (31%)	12 (24%)	26 (35%)
**Interview Language**			
Mandinka	97 (78%)	40 (80%)	57 (77%)
Wolof	27 (22%)	10 (20%)	17 (23%)
**Participant's Occupation**			
Housewife	72 (58%)	32 (64%)	40 (54%)
Other	52 (42%)	18 (36%)	34 (46%)
**Husband's Occupation**			
Skilled Work	47 (38%)	22 (44%)	25 (34%)
Manual/Trade Work	77 (62%)	28 (56%)	49 (66%)

Note. Of those in a polygamous marriage 12 were the first wife, 21 were the second wife and 4 were the third wife. Demographic categories are based on those used by the Ministry of Health and Social Welfare.

Retention and Timeline adherence

For peer review only - http://bmjopen.bmj.com/site/about/guidelines.xhtml

Four antenatal clinics were approached to take part: Pirang Health Centre, Gunjur Health Centre, Sukuta Health Centre and Kuntair Health Centre. All clinics that were approached agreed to participate. Of the 152 women approached, 124 (81.6%) consented and were included in the study and the analysis. 20% declined from the Control group, and 12% declined from the CHIME group. Figure 1 displays the participant flow diagram, indicating the number of participants included at each stage of the study.

Figure 1. CONSORT participant flow diagram. The light blue indicates the Control group and the dark blue indicates the intervention (CHIME) group. The red boxes outline the two 12-week sequences.

The retention and attrition rates across all three data collection time-points and by group are displayed in Supplementary Material 4. Attrition was 20% between baseline and post-intervention assessment, and 33% across all three assessment time-points. There was no difference in attrition between the groups.

The timeline of the stepped wedge schedule could not be strictly adhered to. Figure 2 shows the planned versus the actual timeline of the trial. About 96% [25] of people in the Gambia are Muslim and during Ramadan engaging in musical activities is prohibited. The timeline would have meant that some of the intervention sessions and the final data collection would take place during Ramadan. Therefore, the timeline shifted forward to ensure all data collection and intervention sessions could be completed before Ramadan. This shift meant that the intervention sessions started before the follow-up assessment

For peer review only

For peer review only - http://bmjopen.bmj.com/site/about/guidelines.xhtml

was completed for the control group. However, risk of contamination was small as the intervention sessions happened on non-clinics days when women in the control group were not present and all follow-up data collection for the control group was held on a different day to that of the intervention sessions.

Figure 2. Trial Timeline. The actual trial timeline compared to the planned timeline.

CHIME deliverability

All four Kanyeleng groups were able to deliver all six sessions across the six weeks. Sessions lasted 60 minutes on average. A detailed display of session duration as well as the number of participants in attendance per session per clinic is in Supplementary Material 5. 62% of participants attended at least 4 or more sessions across all the sites and 72% attended at least half (3 sessions). Only 6 participants (12%) did not attend any session. Kuntair had the highest attendance with 100% of participants attending at least 4 sessions. Pirang had the second highest attendance with 76% attending at least 4 sessions followed by Gunjur with 70%. Sukuta, the only clinic within an urban setting, had that lowest attendance overall with 31% of participants attending at least four sessions.

Based on the checklist, both RAs rated each video and found that all recorded sessions included all of the agreed elements. Agreement between the two RAs was 100%.

Acceptability of CHIME

For peer review only

For peer review only - http://bmjopen.bmj.com/site/about/guidelines.xhtml

36 participants within the CHIME group were interviewed across the four clinics. Five FGDs were held, one with the CHNs and one with each of the four Kanyeleng groups. Five higher-level themes were developed: Learning, Peaceful Mind, Social Relationships, Suggestions for the Future, and Evaluations. Within these themes, different categories were created. See Table 2 for the coding structure used and an example except representing each category.

Table 2. Coding structure with examples

High Level Theme	Category	Codes	Example
**Learning**	Care for baby		"The pregnant women can understand how to take care of their children when they give birth" – Kanyeleng
	Coping		"What I learnt was as a pregnant woman don't isolate yourself to be free from lack of peace of mind. Mingle with the people and do everything together." – Pregnant Participant
	Health Information		"It is a taboo to discuss these type of things. So if this kind of programme is implemented a lot of doubts will be cleared regards to pregnancy, delivery and so on. […] If programmes like this are coming we can talk to the antenatal mothers and I think is going to help a lot." – CHN
	Music		"A learned about a lot of songs." – Pregnant Participant
**Peaceful Mind**			"It makes me have peace of mind and makes me happy. When I leave here and go home I don't worry too much. That makes me very happy." – Pregnant Participant
	Continue outside session		"The songs that are sung here when I go home I remember them and that makes me happy. That alone is making me happy. I don't have distress anymore." – Pregnant Participant
**Social Relationships**			
	Outside Relationships		"If you look at the register itself, people were selected from different communities which I think is key. This information is very powerful. You cannot

For peer review only

For peer review only - http://bmjopen.bmj.com/site/about/guidelines.xhtml

			limit it to area because it is disseminated to everybody within that corner" – CHN
		Husband	"Within the midst in my area there is a woman even the husband invited me to explain her wife's problem to him"- Pregnant Participant
		Research Team	"Because you did not forget about us and you are now calling us for an interview. That alone shows that I am a member of the group" – Pregnant Participant
		Teaching others	"Right now I am helping someone and I am a teacher on my own" – Pregnant Participant
	Part of the singing group		"They make friends, they become used to the Kanyelengs, they were creating jokes they were telling them when you deliver we will come and attend your naming ceremony. […] They were used to each other even the day they were going, the last day I felt it" – CHN
**Suggestions for future**			
	Attendance and Participation		"The challenges are there, some of the challenges are before they gather is a problem and other scheduled programmes is also a problem. " – CHN
		Payment	"To encourage pregnant women to join the programme, give them more training and provide them some allowances to motivate them as some of them were complaining about it." – Pregnant Participant
		Transportation	"For somebody who also finds difficult to put food on the table, if you ask that individual even to come once in a week, find it very difficult as some of them have to go in for credit to pay fares to come. If at all something is going to be created for them, everyday you ask how much do they pay for the fare to come, then you give them that so that they […] will make my coming to the session easier." – Kanyeleng
	Breakfast		"We work with pregnant women and they should eat well. They feel lazy when they don't eat. When food is provided, we can even have more hours for the programme. You can consider that for us. We can bear our hunger but for them they cannot" – Kanyeleng
	Continuation		"Can the programme be able to follow the antenatal from six months up to the delivery? When you start and stop within two months maybe before the delivery the woman can have stress. It is important to follow the woman until the delivery like from six

For peer review only - http://bmjopen.bmj.com/site/about/guidelines.xhtml

			months to the delivery so that you can see the impact." – CHN
	Timing		"If you look at these women most of them are business women. They do go to the market to sell, and some of them are alone in their houses so if you want to go beyond the one hour or forty five minutes is going to create a lot of problem" – CHN
**Evaluation**	Negative/ Neutral		"Sometimes transportation is problem" – Pregnant Participant
	Positive		"I am pleased about the programme, because I isolated myself before, and brought a lot thinking on my side. But when I took part in this programme I now go out and mingle with the people to have a chat and my mind has been steady now. " – Pregnant Participant
		Music and performance	"After the programme, my mind has changed because of the songs that were sang there. […] I was lonely when the programme ended. The performance of the Kanyeleng alone bring happiness." – Pregnant Participant
		Session Structure	"The way the songs are organized is good. When you meet with the people you have to greet each other, and when you meet you explain why they are called. When you are dispersing you disperse in peace. We all come together and entertain each other that is good." – Pregnant Participant

For peer review only

The group music intervention was evaluated positively, both by those who ran the sessions and those who participated. Participants thought that the information and strategies they learned in the sessions impacted the larger community. All thought that it was appropriate and the goals were achieved. The suggestions for the future that could be incorporated in a larger trial included extending the length of the intervention, reimbursing transportation costs and providing food. No harms, serious adverse effects or adverse effects were recorded or discussed.

Missing Data

A pronunciation problem by one RA on EPDS item 3 in Mandinka was detected during the baseline data collection for the first sequence. This affected the baseline EPDS scores and resulted in 46 missing values on item 3 of the EPDS. Multiple imputation was used to fill in the missing EPDS item 3 data at baseline, and was used throughout-subsequent analyses.

SRQ-20 and EPDS Baseline Scores

Table 3 displays the descriptive statistics of the SRQ-20 and EPDS scores at baseline. Supplementary Material 6 displays the mean, median and distribution of the SRQ-20 and EPDS scores at all three time-points. The SRQ-20 scores (*M* = 7.27, *SD* = 4.00) are approximately symmetric (skewness = 0.45) at baseline. The distribution of the EPDS (*M* = 4.25, *SD* = 4.10) scores at baseline is highly positively skewed (skewness = 1.48) with the majority of participants presenting few CMD symptoms. There was a significantly lower SRQ-20 baseline score (t (108.02) = 2.46, p < 0.05 95% CIs[0.15, 3.56]) for the CHIME group (*M* = 6.22, *SD* = 3.83) compared to the control group (*M* = 7.97, *SD* = 3.99), and a significantly lower EPDS baseline score (t(121.96) = 3.35, p < 0.01 95%CIs[0.38, 2.91]) for the CHIME group (*M* = 2.90, *SD* = 3.07) compared to the control group (*M* = 5.16, *SD* = 4.46).

For peer review only

Table 3. SRQ-20 and EPDS scores at Baseline

**SRQ-20**	**Baseline**		
	**n**	**Mean (SD)**	**Median (IQR)**
All	124	7.27 (4.00)	7 (4.75–10)
CHIME	50	6.22 (3.83)	6 (3−8.75)

For peer review only - http://bmjopen.bmj.com/site/about/guidelines.xhtml

Control	74	7.97 (3.99)	8 (5−10)

**EPDS**	**Baseline**		
	**n**	**Mean (SD)**	**Median (IQR)**
All	124	4.25 (4.10)	3 (1−5)
CHIME	50	2.90 (3.07)	2 (1−4)
Control	74	5.16 (4.46)	4 (2−6)

Indication of potential efficacy

At the post-intervention assessment there was a mean reduction of 2.13 points (95%CI [0.89, 3.38], $p < 0.01$, $n = 99$) on the SRQ-20 in the CHIME group compared to the control group and a mean reduction of 1.98 points (95% CI [1.06, 2.90], $p < 0.01$, $n = 99$) on the EPDS in the CHIME group compared to the control group.

At the 4-week follow-up assessment a mean reduction of 2.09 points (95%CI [0.76, 3.42], $p < 0.01$, $n = 83$) on the SRQ-20 was found in the CHIME group compared to the control group, and a mean reduction of 0.98 points (95%CI [-0.01, 1.97], $p = 0.05$, $n = 83$) on the EPDS was found in the CHIME group compared to the control group.

DISCUSSION

Overall, the trial was able to address our main objectives and show feasibility, acceptability, and a potential benefit of CHIME compared to standard care in The Gambia. High recruitment levels (81% of those approached consented) and reasonably low attrition rates (20% between baseline and post-intervention, 33% across all three time-points) were found. Feedback from participants and RAs revealed that some attrition could have been due to the women being difficult to contact, through a low phone battery

For peer review only

For peer review only - http://bmjopen.bmj.com/site/about/guidelines.xhtml

or call credit. A future study might help overcome this by providing either phones or phone credit to the participants.

Another way to encourage participant retention would be to implement some of the suggestions discussed in the post-intervention interviews and FGDs. Lack of funds for transportation was a barrier many participants discussed, together with concern that the pregnant women lacked the energy needed to participate in the music making because they were hungry. Traditional Kanyeleng ceremonies and gatherings usually include sharing food [47,48]. Including breakfast and payment for transportation might thus be a helpful addition to the programme. Finally, it was also suggested that CHIME continue longer into pregnancy, following the participants until delivery. Consideration of cost and sustainability would be needed to help address these suggestions [49]. This could be done through the involvement of policymakers through our partners at the Ministry of Health and Social Welfare in The Gambia and through a cost-effectiveness analysis.

As discussed, the timeline of the intervention needed to be shifted forward due to Ramadan. In addition, the rainy season (July–October) can impact travel [50,51]. A key design feature of the stepped wedge is being able to implement the intervention at precise time points in the calendar. Due to seasonal factors (rainy season) and the religious calendar (Ramadan), this design is likely to be difficult to achieve on a larger scale. As a result, alternative approaches for a definitive trial that might be considered are a randomised cluster or cluster crossover trial. These will be less dependant on specific intervals between starting dates [52].

For peer review only

In terms of deliverability, we observed relatively high attendance, with 72% of participants attending at least half of the six sessions. More women in urban areas have a job in addition to their house work and many do not live with extended family members who can help with childcare [25]. Therefore, women in the urban areas, like Sukuta, might have more time constraints making it difficult for them to attend contributing to the lower attendance observed at this clinic. Suggested modifications, such as payment to cover travel, the addition of food, or more potential sessions to attend might help with attendance within an urban context.

The audio and video analysis confirmed the fidelity of the intervention. In general, the intervention was found to be universally acceptable and enjoyable. Interviews and FGDs revealed the reach of the intervention went beyond that of the individuals involved. Participants explained how they had used what they had learned in the intervention sessions to educate other women in their communities. This extension of the intervention has been discussed as one of the benefits of community-led mental health interventions, giving those involved the agency in supporting and teaching others [53].

There was a notable difference in the distribution pattern found with the two measurement tools used, the EPDS and the SRQ-20. This may well relate to the items included in the two scales. The SRQ-20 includes somatic items of mental distress (e.g. headaches, shaking hands) while the EPDS purposefully excludes such items. Previous studies have shown that in an African context, it is the somatic symptoms that manifest

For peer review only - http://bmjopen.bmj.com/site/about/guidelines.xhtml

most clearly in those women with particularly raised levels of psychiatric distress, making the SRQ-20 an appropriate tool [33,54]. The SRQ-20's binomial response format, compared to the EPDS likert scale response format, also means that it is more easily administered and understood by respondents with low literacy. However, both scales showed a clear difference between the CHIME and Control group suggesting that CHIME had helped reduce both psychological and somatic symptoms.

The differences between the two groups at the post-intervention assessment showed a medium effect size. To out knowledge this makes this study the first to provide information on the size of effect that could be expected from a population level intervention when using the SRQ-20. This finding will not only be helpful for a future scaled up trial using CHIME but also for other population level community mental health interventions trials within LMICs.

Limitations

While the overall recruitment rate was high (81%), fewer women were recruited into the CHIME group (*n* = 50) compared to the Control group (*n* = 74). Feedback from the RAs indicated that this was potentially due to the frequency of available clinic days to complete recruitment. Additionally, selection bias was possible. It may have been that those with more work or family pressures might be less likely to be able to afford the time to take off for the intervention. Any future trial would need to address this potential bias. Participants were not blinded to which group they were assigned, increasing the potential for response bias, a problem faced by all research studies that are unblinded and

For peer review only - http://bmjopen.bmj.com/site/about/guidelines.xhtml

use self-reporting [55]. A future study might help reduce the effect of these biases through a different trial design with a more extended recruitment period and a consenting process whereby participants are not aware of their group assignment until after they have agreed to take part.

Participants' baseline antenatal EPDS and SRQ-20 scores were significantly lower in the CHIME group compared to the Control group. This difference might have also been due to those with less time constraints, and potentially less stress, being more likely to take part in the intervention. Although these differences could partly be accounted for within the analysis, a blind consenting process could also help lessen their effect within a future trial.

CONCLUSION

We have demonstrated that a community psycho-social music intervention (CHIME) is acceptable and feasible to deliver in The Gambia as a way to reduce symptoms of antenatal CMDs. We have identified a potential beneficial effect that will need to be confirmed through a future definitive trial. Post-intervention interviews gave valuable information and feedback on the intervention and operational aspects of the design, payment, transportation and session structure. No previous study has been identified that investigates the potential of a community music intervention for perinatal mental health in a LMIC and the feasibility outcomes reported here highlight the potential of such an approach.

For peer review only

For peer review only - http://bmjopen.bmj.com/site/about/guidelines.xhtml

Ethics approval and consent to participate

Ethical approval was obtained from the Goldsmiths University Ethics Committee, the Gambia Government/MRC Gambia joint ethics committee and the Australian National University ethics committee. Members of the research team carried out the consenting and conduct of this study orally. It was emphasised that any participant was able to withdraw from the study at any point without any consequences.

Competing interests

The authors declare that they have no competing interests.

Funding and sponsorship

This study was funded by the MRC-AHRC Global Public Health: Partnership Awards scheme (MR/R024618/1) awarded to Prof Lauren Stewart. The funders (MRC and AHRC) and sponsor (Goldsmiths) have no roles or responsibilities in the design, conduct, data analysis and interpretation, manuscript writing, and dissemination of results.

Data availability

Deidentified participant data that underline the results reported in this article are available upon reasonable request from the corresponding author (ksanf001@gold.ac.uk).

Author contributions

The manuscript was prepared by KRS and all authors approved the final draft. KRS and BM helped with data management and data collection materials. BD provided local

For peer review only

knowledge and organization in the field liaising with Kanyeleng groups and antenatal clinics. HC provided management of the funds and transportation in The Gambia. HBH and MG completed all data collection and input. KRS completed data cleaning and analysis. VC provided methodological and statistical expertise. VG and PR provided expertise in perinatal mental health. IC and BM provided expertise on music and its role in health and community. All authors, except VG and IC, spent time in The Gambia supervising the project on the ground. LS is the grant holder and principal investigator and oversaw all aspects of the study.

Acknowledgements

We would like to thank the Kanyeleng groups and the participants for taking part in the study. Thank you also to Pa Bakary Sonko, Charlotte Hanlon and Catherine Carr for their essential advice and support and Jane Offerman for helping with the administration of this project.

REFERENCES

1 Fisher J, de Mello MC, Patel V, *et al.* Prevalence and determinants of common perinatal mental disorders in women in low- and lower-middle-income countries: A systematic review. *Bull World Health Organ* 2012;**90**:139–49. doi:10.2471/BLT.11.091850

2 World Health Organization. The international classification of diseases, 10th edition (ICD- 10). Geneva, Swizerland: 2010. doi:10.1016/j.nurpra.2008.09.020

3 Glover V, O’Donnell KJ, O’Connor TG, *et al.* Prenatal maternal stress, fetal programming, and mechanisms underlying later psychopathology - A global perspective. *Dev Psychopathol* 2018;**30**:843–54. doi:10.1017/S095457941800038X

4 Martins C, Gaffan EA. Effects of early maternal depression on patterns of infant–mother attachment: A meta-analytic investigation. *J Child Psychol Psychiatry Allied Discip* 2000;**41**:737–46.

5 Stein A, Pearson RM, Goodman SH, *et al.* Effects of perinatal mental disorders on

For peer review only - http://bmjopen.bmj.com/site/about/guidelines.xhtml

the fetus and child. *Lancet* 2014;**384**:1800–19. doi:10.1016/S0140-6736(14)61277-0
6 Milgrom J, Gemmill AW, Bilszta JL, *et al.* Antenatal risk factors for postnatal depression: A large prospective study. *J Affect Disord* 2008;**108**:147–57. doi:10.1016/j.jad.2007.10.014
7 Robertson E, Grace S, Wallington T, *et al.* Antenatal risk factors for postpartum depression: A synthesis of recent literature. *Gen Hosp Psychiatry* 2004;**26**:289–95. doi:10.1016/j.genhosppsych.2004.02.006
8 Glover V. Prenatal stress and its effects on the fetus and the child: Possible Underlying biological mechanisms. In: Antonelli MC, ed. *Perinatal Programming of Neurodevelopment*. Advances in Neurobiology 2015. 269–83. doi:10.1007/978-1-4939-1372-5_13
9 Ramchandani PG, Richter LM, Norris SA, *et al.* Maternal prenatal stress and later child behavioral problems in an urban South African setting. *J Am Acad Child Adolesc Psychiatry* 2010;**49**:239–47. doi:10.1097/00004583-201003000-00007
10 Rahman A, Fisher J, Bower P, *et al.* Interventions for common perinatal mental disorders in women in low- and middle-income countries: A systematic review and meta-analysis. *Bull World Health Organ* 2013;**91**:593-601I. doi:10.2471/BLT.12.109819
11 Semrau M, Alem A, Abdulmalik J, *et al.* Developing capacity-building activities for mental health system strengthening in low- and middle-income countries for service users and caregivers, service planners, and researchers. *Epidemiol Psychiatr Sci* 2017;:1–11. doi:10.1017/S2045796017000452
12 Semrau M, Lempp H, Keynejad R, *et al.* Service user and caregiver involvement in mental health system strengthening in low- and middle-income countries: Systematic review. *BMC Health Serv Res* 2016;**16**:79. doi:10.1186/s12913-016-1323-8
13 Williams E, Dingle GA, Clift S. A systematic review of mental health and wellbeing outcomes of group singing for adults with a mental health condition. *Eur J Public Health* 2018;**28**:1035–42. doi:10.1093/eurpub/cky115
14 Terry MM, Terry DR. Singing the Blues: A literature review of the effects of music on postnatal depression. *Int J Innov Interdiscip* 2012;**1**:55–67.
15 Wulff V, Hepp P, Fehm T, *et al.* Music in obstetrics: An intervention option to reduce tension, pain and stress. *Geburtshilfe Frauenheilkd* 2017;**77**:967–75. doi:10.1055/s-0043-118414
16 Fancourt D, Perkins R. Effect of singing interventions on symptoms of postnatal depression: Three-arm randomised controlled trial. *Br J Psychiatry* 2018;**212**:119–21. doi:10.1192/bjp.2017.29
17 Clift S, Hancox G, Morrison I, *et al.* Group singing as a public health resource. In: Clift S, Camic PM, eds. *Oxford Textbook of Creative Arts, Health, and Wellbeing: International Perspectives on Practice, Policy and Research*. Oxford, UK: : Oxford University Press 2015. 251–8.
18 Dingle GA, Clift S, Finn S, *et al.* An agenda for best practice research on group singing , health , and well-being. *Music Sci* 2019;**2**:1–15. doi:10.1177/2059204319861719
19 Pearce E, Launay J, Dunbar RIM. The ice-breaker effect: Singing mediates fast

For peer review only

For peer review only - http://bmjopen.bmj.com/site/about/guidelines.xhtml

social bonding. *R Soc Open Sci* 2015;**2**:150221. doi:10.1098/rsos.150221

20 McConnell BB. Music and health communication in The Gambia: A social capital approach. *Soc Sci Med* 2016;**169**:132–40. doi:10.1016/j.socscimed.2016.09.028

21 Bastien S. Reflecting and shaping the discourse: The role of music in AIDS communication in Tanzania. *Soc Sci Med* 2009;**68**:1357–60. doi:10.1016/j.socscimed.2009.01.030

22 Silver D. Songs and storytelling: Bringing health messages to life in Uganda. *Educ Heal* 2001;**14**:51–60. doi:10.1080/13576280010015362

23 World Bank. The Gambia overview. 2019.https://www.worldbank.org/en/country/gambia/overview (accessed 4 Oct 2019).

24 McConnell BB. Gambia: History, culture, and geography of music. In: Sturman J, ed. *The SAGE International Encyclopedia of Music and Culture*. Thousand Oaks: : SAGE Publications, Inc. 2019. 974–5. doi:10.4135/9781483317731.n305

25 The Gambia Bureau of Statistics (GBOS) and ICF International. The Gambia Demographic and Health Survey 2013. Banjul, The Gambia and Rockville, Maryland, USA: 2014.

26 World Bank. World development indicators 2015. Washington DC: 2015. doi:10.1596/978–1-4648–0440–3.

27 McConnell BB. *Singing the unsayable: Female performers and global health in The Gambia*. 2015.

28 McConnell BB. Performing ‘participation’: Kanyeleng musicians and global health in The Gambia. *Ethnomusicology* 2017;**61**:312–32.

29 Sanfilippo KRM, McConnell B, Cornelius V, *et al.* A study protocol for testing the feasibility of a randomised stepped wedge cluster design to investigate a Community Health Intervention through Musical Engagement (CHIME) for perinatal mental health in The Gambia. *Pilot Feasibility Stud* 2019;**5**:124. doi:10.1186/s40814-019-0515-5

30 Eldridge SM, Chan CL, Campbell MJ, *et al.* CONSORT 2010 statement: Extension to randomised pilot and feasibility trials. *BMJ* 2016;**355**. doi:10.1136/bmj.i5239

31 Sim J, Lewis M. The size of a pilot study for a clinical trial should be calculated in relation to considerations of precision and efficiency. *J Clin Epidemiol* 2012;**65**:301–8. doi:10.1016/j.jclinepi.2011.07.011

32 World Health Organization. Process of translation and adaptation of instruments. 2014.

33 Hanlon C, Medhin G, Alem A, *et al.* Detecting perinatal common mental disorders in Ethiopia: Validation of the self-reporting questionnaire and Edinburgh Postnatal Depression Scale. *J Affect Disord* 2008;**108**:251–62. doi:10.1016/j.jad.2007.10.023

34 Cox JL, Holden J, Henshaw C. *Perinatal mental health: The Edinburgh Postnatal Depression Scale (EPDS) manual*. 2nd ed. London, UK: : The Royal College of Psyciatrists Publications 2014.

35 Beusenberg M, Orley J. A user’s guide to the Self Reporting Questionnaire (SRQ). 1994.

36 Sawyer A, Ayers S, Smith H. Pre- and postnatal psychological wellbeing in

For peer review only

Africa: A systematic review. *J Affect Disord* 2010;**123**:17–29. doi:10.1016/j.jad.2009.06.027

37 Kozinszky Z, Dudas RB. Validation studies of the Edinburgh Postnatal Depression Scale for the antenatal period. *J Affect Disord* 2015;**176**:95–105. doi:10.1016/j.jad.2015.01.044

38 Tsai AC, Scott JA, Hung KJ, *et al.* Reliability and validity of instruments for assessing perinatal depression in African settings: Systematic review and meta-analysis. *PLoS One* 2013;**8**:e82521. doi:10.1371/journal.pone.0082521

39 Coleman R, Morison L, Paine K, *et al.* Women's reproductive health and depression: A community survey in The Gambia, West Africa. *Soc Psychiatry Psychiatr Epidemiol* 2006;**41**:720–7. doi:10.1007/s00127-006-0085-8

40 Nabwera HM, Moore SE, Mwangome MK, *et al.* The influence of maternal psychosocial circumstances and physical environment on the risk of severe wasting in rural Gambian infants: A mixed methods approach. *BMC Public Health* 2018;**18**:109. doi:10.1186/s12889-017-4984-2

41 Bartram C. *Early infant social behaviour and maternal perinatal depression in Kiang West , The Gambia*. 2018.

42 Chan AW, Tetzlaff JM, Gøtzsche PC, *et al.* SPIRIT 2013 explanation and elaboration: Guidance for protocols of clinical trials. *BMJ* 2013;**346**:e7586. doi:10.1136/bmj.e7586

43 van Buuren S, Groothuis-Oudshoorn K. mice: Multivariate imputation by chained equations in R. *J Stat Softw* 2011;**45**:1–67.

44 R Core Team. R: A language and environment for statistical computing. 2013.http://www.r-project.org/

45 Braun V, Clarke V. Using thematic analysis in psychology. *Qual Res Psychol* 2006;**3**:77–101. doi:10.1191/1478088706qp063oa

46 Dedoose version 7.0.23. Dedoose. dedoose.com. 2016.

47 McConnell BB. *Music, health, and power: Singing the unsayable in The Gambia*. New York, NY: : Routledge 2020. doi:10.4324/9780367312732

48 Saho B. Ritualizing and domesticating space: 'Kañeleng' women coping with childlessness in the Gambia. *Mand Stud* 2012;**14**:99–125. doi:10.2307/44080973

49 Petersen I, Evans-Lacko S, Semrau M, *et al.* Promotion, prevention and protection: Interventions at the population- and community-levels for mental, neurological and substance use disorders in low- and middle-income countries. *Int J Ment Health Syst* 2016;**10**:30. doi:10.1186/s13033-016-0060-z

50 Fardi SA. *Neonatal hair cortisol in relation to maternal environment, sociodemographic factors, and infant birth and growth outcomes in rural Gambia*. Anthropol. Grad. Thesis Diss. 2017.

51 Moore SE, Cole TJ, Poskitt EME, *et al.* Season of birth predicts mortality in rural Gambia. *Nature* 1997;**388**:434. doi:10.1038/41245

52 Arnup SJ, McKenzie JE, Hemming K, *et al.* Understanding the cluster randomised crossover design: A graphical illustration of the components of variation and a sample size tutorial. *Trials* 2017;**18**:381. doi:10.1186/s13063-017-2113-2

53 Kohrt BA, Asher L, Bhardwaj A, *et al.* The role of communities in mental health care in low-and middle-income countries: A meta-review of components and competencies. *Int J Environ Res Public Health* 2018;**15**:1279.

For peer review only

doi:10.3390/ijerph15061279

54 Senturk V, Hanlon C, Medhin G, *et al.* Impact of perinatal somatic and common mental disorder symptoms on functioning in Ethiopian women: The P-MaMiE population-based cohort study. *J Affect Disord* 2012;**136**:340–9. doi:10.1016/j.jad.2011.11.028

55 Chan D. So why ask me? Are self-report data really that bad? In: Lance CE, Vandenberg RJ, eds. *Statistical and Methodological Myths and Urban Legends: Doctrine, Verity and Fable in the Organizational and Social Sciences*. Routledge/Taylor & Francis Group 2008. 309–36. doi:10.4324/9780203867266

For peer review only

For peer review only - http://bmjopen.bmj.com/site/about/guidelines.xhtml

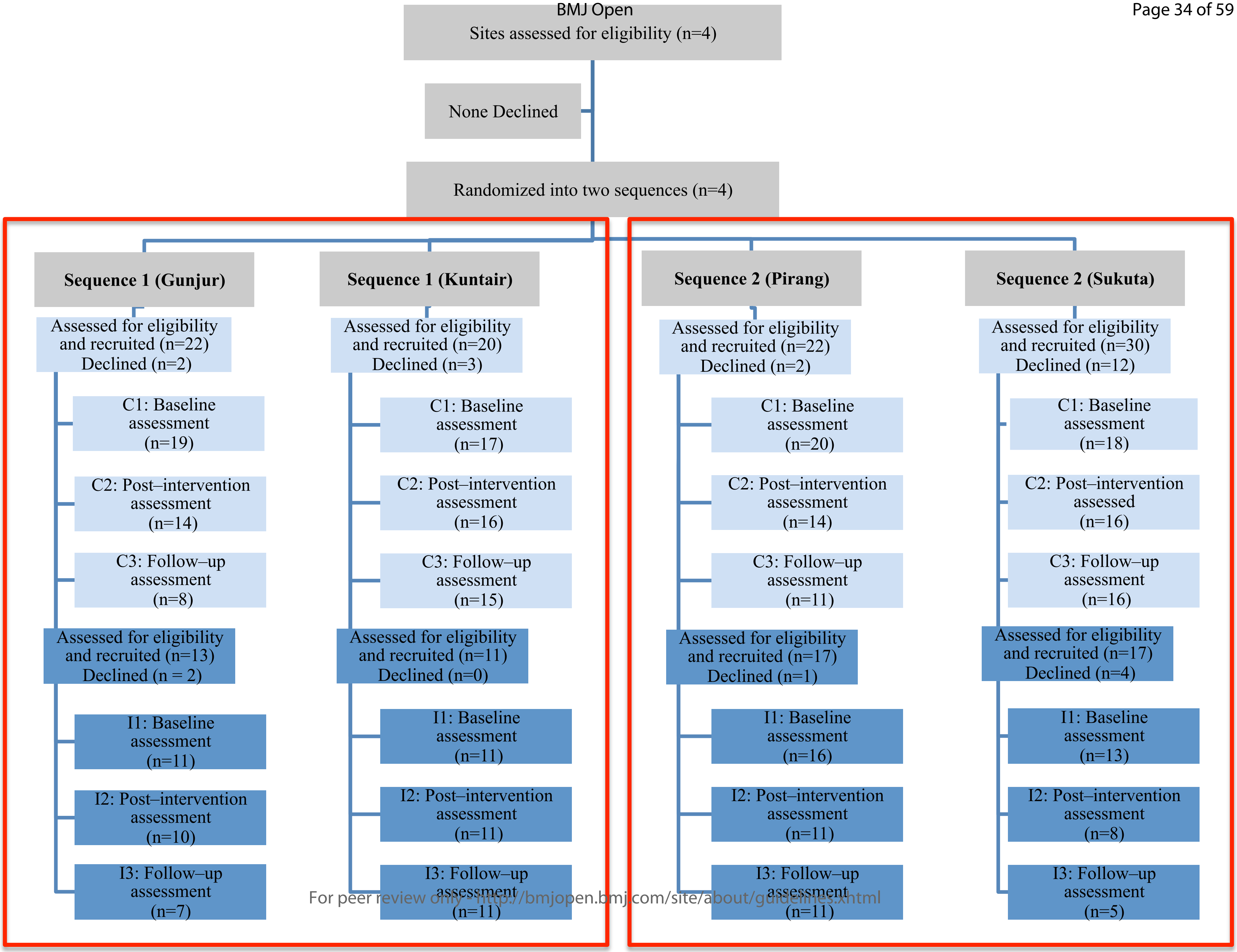
Sites assessed for eligibility (n=4)
None Declined
Randomized into two sequences (n=4)
Sequence 1 (Gunjur)
Assessed for eligibility and recruited (n=22) Declined (n=2)
C1: Baseline assessment (n=19)
C2: Post–intervention assessment (n=14)
C3: Follow–up assessment (n=8)
Assessed for eligibility and recruited (n=13) Declined (n = 2)
I1: Baseline assessment (n=11)
I2: Post–intervention assessment (n=10)
I3: Follow–up assessment (n=7)
Sequence 1 (Kuntair)
Assessed for eligibility and recruited (n=20) Declined (n=3)
C1: Baseline assessment (n=17)
C2: Post–intervention assessment (n=16)
C3: Follow–up assessment (n=15)
Assessed for eligibility and recruited (n=11) Declined (n=0)
I1: Baseline assessment (n=11)
I2: Post–intervention assessment (n=11)
I3: Follow–up assessment (n=11)
Sequence 2 (Pirang)
Assessed for eligibility and recruited (n=22) Declined (n=2)
C1: Baseline assessment (n=20)
C2: Post–intervention assessment (n=14)
C3: Follow–up assessment (n=11)
Assessed for eligibility and recruited (n=17) Declined (n=1)
I1: Baseline assessment (n=16)
I2: Post–intervention assessment (n=11)
I3: Follow–up assessment (n=11)
Sequence 2 (Sukuta)
Assessed for eligibility and recruited (n=30) Declined (n=12)
C1: Baseline assessment (n=18)
C2: Post–intervention assessed (n=16)
C3: Follow–up assessment (n=16)
Assessed for eligibility and recruited (n=17) Declined (n=4)
I1: Baseline assessment (n=13)
I2: Post–intervention assessment (n=8)
I3: Follow–up assessment (n=5)

Planned Timeline

Time point:		1	2	3	4	5	6	7	8	9	10	11	12	13	14	15	16	17	18	19	20	21	22	23	24	25	26	27	28	29	30	31	32	33	34	35	36
Sequence 1	Cluster 1	C1	X	X	X	X	X	X	C2				C3																								
														I1	O	O	O	O	O	O	I2				I3												
	Cluster 2	C1	X	X	X	X	X	X	C2				C3																								
														I1	O	O	O	O	O	O	I2				I3												
Sequence 2	Cluster 3													C1	X	X	X	X	X	X	C2				C3												
																										I1	O	O	O	O	O	O	I2				I3
	Cluster 4													C1	X	X	X	X	X	X	C2				C3												
																										I1	O	O	O	O	O	O	I2				I3

For peer review only

Actual Timeline

Time point:		1	2	3	4	5	6	7	8	9	10	11	12	13	14	15	16	17	18	19	20	21	22	23	24	25	26
Sequence 1	Cluster 1	C1	X	X	X	X	X	X	C2				C3														
											I1	O	O	O	O	O	O	I2				I3					
	Cluster 2	C1	X	X	X	X	X	X	C2				C3														
											I1	O	O	O	O	O	O	I2				I3					
Sequence 2	Cluster 3						C1	X	X	X	X	X	X	C2				C3									
																I1	O	O	O	O	O	O	I2				I3
	Cluster 4						C1	X	X	X	X	X	X	C2				C3									
																I1	O	O	O	O	O	O	I2				I3

Time point = (1 week)

C = Control

I = Intervention

C1 = Control group baseline assessment

C2 = Control group post–intervention assessment

C3 = Control group follow–up assessment

I1 = Intervention group baseline assessment

I2 = Intervention group post–intervention assessment

I3 = Intervention group follow–up assessment

Supplementary Material 1

Intervention Development Workshop Discussion points

These six sessions are intended to support women emotionally and psychologically during their pregnancy.

Session Goals
During the session, we hope that women will participate by singing songs, clapping hands, and moving to the music. Also, we would like you to share some brief information about how to cope with common physical and psychological symptoms of pregnancy. We hope that the women will finish the session

- in a *positive mood*
- feeling part of a socially-supportive group
- with some new strategies of how to cope with some of the common physical and psychological challenges of pregnancy
- equipped with skills to seek wider social support even outside the sessions

Key Messages
Our suggestion is that sessions concentrate on various different themes as follows [discuss with the Kanyeleng group whether these topics seem suitable, allow them to share examples of songs they could use and additional ideas they may have]:

1) Common physical and psychological symptoms of pregnancy and ways to cope such as:
- Importance of the singing group in supporting each other
- Importance of other positive relationships in their lives
- Resilience to challenges and empowerment
- Importance of being open, removing stigma to discuss challenges

2) Learning lullabies for after birth
3) How to care for the baby after birth

Session Structure
A typical session might involve:

- A welcome song at the start
- A middle section that would include one song that would focus on the topic of the week (see above), and others to promote a positive mood and encourage the women to move together.
- A closing song to draw the session to a close

For peer review only

How to run a successful session

Throughout the sessions we would like you to act as 'facilitators' rather than 'teachers'. We would like you to:

- Draw on your existing skills and ways of working to help us fulfill the goals described above.

- Encourage the women to be involved in whatever way they would like (please be aware the some women may not be comfortable to move very vigorously, and should feel encouraged to move in whatever way is comfortable). If someone is not joining in, check if they are okay, and try to engage them in a way that doesn't make them self-conscious.

- Notice how the women are responding to the activities that you initiate – is the pace too slow, too fast, too loud etc.? Try to adapt what you are doing according to the responses you see.

- Although it is fine to mention that pregnancy can involve challenges, it is important not to encourage the women to dwell on these, but instead to think about coping strategies and supporting each other, to ensure that the mood is a positive one.

For peer review only

Supplementary Material 2

SRQ-20
Mandinka

The following questions are related to certain pains and problems that may have bothered you in the last 30 days. If you think the question applies to you and you have had the described problem in the last 30 days, answer YES.
On the other hand, if the question does not apply to you and you did not have the problem in the last 30 days, answer NO.
If you are unsure about how to answer a question, please give the best answer you can.

Nying nyininkari dantangolu i be dending dimoo doolu la aning mantooro doolu la meng y'a long a si ke a ye batandi nying tili tansabo meng tambita.
N'ii y'a mira ko nyininkaroo nying ye maa le, aning i ye nying mantooro sifa soto nying tili tansabo kono, i s'a fo haa.
N'ii y'a tara nyininkaroo nying m'ee maa, aning i mang nying mantooro sifa soto nying tili tansabo kono, i s'a fo haani.

1. Do you often have headaches?<br><br>1. Fo i ka kundimoo soto le waati ning waati?	☐$_1$. Yes	☐$_0$. No
2. Is you appetite poor?<br><br>2. Fo domoro buka diyaa i daa kono?	☐$_1$. Yes	☐$_0$. No
3. Do you sleep badly?<br><br>3. Fo ila siinoo buka beteyaa le bang?	☐$_1$. Yes	☐$_0$. No

4. Are you easily frightened?<br><br>4. Fo i ka tariyaa ka silla le bang?	□1. Yes	□0. No
5. Do your hands shake?<br><br>5. Fo i buloolu ka jarajara?	□1. Yes	□0. No
6. Do you feel nervous, tense or worried?<br><br>6. I ka fitii le bang wara sondomoo tenkung baliyaa k'e batandi le bang?	□1. Yes	□0. No
7. Is your digestion poor?<br><br>7. Fo n'ii ye domoro ke a buka tariyaa ka jii le bang?	□1. Yes	□0. No
8. Do you have trouble thinking clearly?<br><br>8. Fo miralotenkung baliyaa k'e batandi le bang?	□1. Yes	□0. No
9. Do you feel unhappy?<br><br>9. Fo i ka niitoroo soto le bang?	□1. Yes	□0. No

For peer review only

10. Do you cry more than usual?<br><br>10. Fo i ka kumboo danna tambiringo ke le bang?	□1. Yes	□0. No
11. Do you find it difficult to enjoy your daily activities?<br><br>11. Fo i buka kontan ka ila lung o lung haajoolu ke le bang?	□1. Yes	□0. No
12. Do you find it difficult to make decisions?<br><br>12. Fo I ka bataa kuwo soto le ka hajoolu t'ee i fango ye?	□1. Yes	□0. No
13. Is your daily work suffering?<br><br>13. I ka naasoo soto ila lung o lung dookuwo to bang?	□1. Yes	□0. No
14. Are you unable to play a useful part in life?<br><br>14. Fo i ka koleya kuwo soto ka nafaa kuwolu tamandi dunia baluwo kono?	□1. Yes	□0. No
15. Have you lost interest in things?<br><br>15. Fo ila lafoo talaata fengolu to i ka lafi meng na nung?	□1. Yes	□0. No

For peer review only

16. Do you feel that you are a worthless person?<br><br>16. Fo i k'e fango je ko i nafaa soto bang?	□1. Yes	□0. No
17. Has the thought of ending your life been on your mind?<br><br>17. Fo waati do ka soto i k'e mira pur ka i fango faa?	□1. Yes	□0. No
18. Do you feel tired all the time?<br><br>18. Fo i ka tara bataring waati o waati le bang?	□1. Yes	□0. No
19. Do you have uncomfortable feelings in your stomach?<br><br>19. Fo i kono k'e batandi le bang?	□1. Yes	□0. No
20. Are you easily tired?<br><br>20. Fo i ka tariyaa ka bataa?	□1. Yes	□0. No

For peer review only

SRQ-20
Wolof

The following questions are related to certain pains and problems that may have bothered you in the last 30 days. If you think the question applies to you and you have had the described problem in the last 30 days, answer YES.
On the other hand, if the question does not apply to you and you did not have the problem in the last 30 days, answer NO.
If you are unsure about how to answer a question, please give the best answer you can.

Lache yii daffa khetog meetit walla jeffeh-jeffeh yii nga khamneh manala sohnal si birr fenwerry-fen yu passeh. So fongehneh lache bi andana ak sa halat teh am nga meetit yii ak jeffeh-jeffeh yii si fenwerry-fen yu passeh, tontuma WAAW
Si benen borrbi, su lache bi andutak lee nga halat si yaw teh amulo jeffeh-jeffeh yii si birr fenwerry-fen yu passeh, tontma DADET
Su feekeh danga ham nyarry khel si negga warra tontor lache bi, ngela wyyy jokhma tontu bi nga fogneh mo ngane.

1. Do you often have headaches?<br><br>1. Ndakh dang de ham bopa bu meeti lego lake?	□$_1$. Yes	□$_0$. No
2. Is you appetite poor?<br><br>2. Ndakh sa leeka bi daffa wanyekou?	□$_1$. Yes	□$_0$. No
3. Do you sleep badly?<br><br>3. Ndakh dang de ham nelew bu bon?	□$_1$. Yes	□$_0$. No

For peer review only

4. Are you easily frightened?<br><br>4. Ndakh danga de gawa tit?	□1. Yes	□0. No
5. Do your hands shake?<br><br>5. Ndakh sa lohoyee daff de lokh?	□1. Yes	□0. No
6. Do you feel nervous, tense or worried?<br><br>6. Ndakh dang de yeek titangeh wala d jahleh?	□1. Yes	□0. No
7. Is your digestion poor?<br><br>7. Ndakh dang de ham leeka budut gawa ress?	□1. Yes	□0. No
8. Do you have trouble thinking clearly?<br><br>8. Ndakh dang de ham jeffeh-jeffeh si halat bu bakh?	□1. Yes	□0. No
9. Do you feel unhappy?<br><br>9. Ndakh dang de nyaka kontan?	□1. Yes	□0. No

For peer review only

10. Do you cry more than usual?<br><br>10. Ndakh dang de bug d joi lu barre?	□1. Yes	□0. No
11. Do you find it difficult to enjoy your daily activities?<br><br>11. Ndakh danga de ham jeffeh-jeffeh purr ham kontateh si leenga warra deff si bess bi?	□1. Yes	□0. No
12. Do you find it difficult to make decisions?<br><br>12. Ndakh dang de ham jeffeh-jeffeh purr deff saye halati boppa?	□1. Yes	□0. No
13. Is your daily work suffering?<br><br>13. Ndakh dor de ham khat-khat si sa leugue bess bi?	□1. Yes	□0. No
14. Are you unable to play a useful part in life?<br><br>14. Ndakh dang de ham jeffeh-jeffeh si lo hmneh dinala njering sisa ngerre dundu?	□1. Yes	□0. No
15. Have you lost interest in things?<br><br>15. Ndakh dor de nyaka eteh si lega warra deff	□1. Yes	□0. No

For peer review only

16. Do you feel that you are a worthless person?<br><br>16. Ndakh dang de ham yeek-yeek ne yaw dor darra?	□$_1$. Yes	□$_0$. No
17. Has the thought of ending your life been on your mind?<br><br>17. Ndakh danga de tog di halat neh dehh mo la ngenal?	□$_1$. Yes	□$_0$. No
18. Do you feel tired all the time?<br><br>18. Ndakh dang de soona wakhtu bu neeka?	□$_1$. Yes	□$_0$. No
19. Do you have uncomfortable feelings in your stomach?<br><br>19. Ndakh dang de ham yeek-yeek ngi jeffeh-jeffeh birr?	□$_1$. Yes	□$_0$. No
20. Are you easily tired?<br><br>20. Ndakh dang de gawa soona?	□$_1$. Yes	□$_0$. No

For peer review only

EPDS
Mandinka

Please tick the answer which comes closest to how you have felt *in the past week, not just how you feel today*. There are no right or wrong answers and please feel free to answer honestly as these questionnaires are confidential.

Bao saaying kono bee la, waranta i wuluuta a mang mee, m be lafi la ka a long i be nyaadi le. M be nyininkaroo ke la ka juubee i be nyaadii le tili worowulo nying kono, a manke i ye i fango je nyaameng bii damma.

1. Have you been able to laugh and see the funny side of things?<br><br>1. Fo i ka jele aning fo i ka jelekuwolu fanang kalamuta?	☐0.<br><br>As much as I always could<br><br>Haa, baake fango n lafita ala nyaameng	☐1.<br><br>Not quite so much now<br><br>A buka siyaa baake ko a be nung nyaameng	☐2.<br><br>Definitely not so much now<br><br>A buka siyaa fereng	☐3.<br><br>Not at all<br><br>M buka jele fereng
2. Have you looked forward with enjoyment to things?<br><br>2. Fo i ka niidiyaa kuwolu jamaa le nyaatosii?	☐0.<br><br>As much as I ever did<br><br>Haa, baake fango n lafita ala nyaameng	☐1.<br><br>Rather less than I used to<br><br>A buka siyaa baake ko a be nung nyaameng	☐2.<br><br>Definitely less than I used to<br><br>A buka siyaa fereng	☐3.<br><br>Hardly at all<br><br>Haani, m buka feng nyaatosii

For peer review only

3. Have you blamed yourself unnecessarily when things went wrong?<br><br>3. Fo i k'e fango jalai kensengke le ning kuwolu mang taa a nyaama?	☐3.<br><br>Yes, most of the time<br><br>Haa, waati jamaa	☐2.<br><br>Yes, some of the time<br><br>Haa, waati doolu	☐1.<br><br>Not very often<br><br>Haani, a mang siyaa	☐0.<br><br>No, never<br><br>Haani, mbuka wo ke fereng
4. Have you been anxious or worried for no good reason?<br><br>4. Fo i ka fiti le waranta i ka tara detering ne i fango feyi a ye a tara a mang jara wo la?	☐0.<br><br>No, not at all<br><br>Haani, m buka wo ke fereng	☐1.<br><br>Hardly ever<br><br>Haani, a mang siyaa	☐2.<br><br>Yes, sometimes<br><br>Haa, waati doolu	☐3.<br><br>Yes, very often<br><br>Haa, waati jamaa
5. Have you felt scared or panicky for no good reason?<br><br>5. Fo i ka silla wara i kijo k'e fara a y'a tara a mang jara wo la?	☐3.<br><br>Yes, quite a lot<br><br>Haa, waati jamaa	☐2.<br><br>Yes, sometimes<br><br>Haa, waati doolu	☐1.<br><br>No, not much<br><br>Haani, a buka siyaa	☐0.<br><br>No, not at all<br><br>Haani, mbuka wo ke

For peer review only

6. Have things been getting on top of you?<br><br>6. Hajoolu k'e detendi le fo a ka koleyaa i bulu ka ila kuwolu tamandi?	☐3.<br><br>Yes, most of the time I haven't been able to cope at all<br><br>Haa, jamaa jamaa a ka koleyaa m bulu	☐2.<br><br>Yes, sometimes I haven't been coping as well as usual<br><br>Haa, waati doolu a ka koleyaa	☐1.<br><br>No, most of the time I have coped quite well<br><br>Haani, jamaa jamaa a buka koleya m bulu	☐0.<br><br>No, I have been coping as well as ever<br><br>Haani, n ka nna kuwolu tamandi noo n ka ke nung nyaameng
7. Have you been so unhappy that you have had difficulty sleeping?<br><br>7. Fo kontaani baliyaa le ka tinna i buka siinoo noo a nyaama?	☐3.<br><br>Yes, most of the time<br><br>Haa, waati o waati	☐2.<br><br>Yes, sometimes<br><br>Haa, waati doolu	☐1.<br><br>Not very often<br><br>Haani, a buka siyaa	☐0.<br><br>No, not at all<br><br>Haani, wo mang soto
8. Have you felt sad or miserable?<br><br>8. Fo niikuyaa waranta niitooroo k'e batandi le bang?	☐3.<br><br>Yes, most of the time<br><br>Haa, waati o waati	☐2.<br><br>Yes, quite often<br><br>Haa, waati doolu	☐1.<br><br>Not very often<br><br>Haani, a buka siyaa	☐0.<br><br>No, not at all<br><br>Haani, wo buka m batandi

For peer review only

9. Have you been so unhappy that you have been crying?<br><br>9. Fo i ka tara kontaani baliyaa le kono fo i ka kumboo?	☐3.<br><br>Yes, most of the time<br><br>Haa, waati jamaa	☐2.<br><br>Yes, quite often<br><br>Haa, waati ning waati	☐1.<br><br>Only occasionally<br><br>Haa, bari a mang siyaa	☐0.<br><br>No, never<br><br>Haani, a mang soto
10. Has the thought of harming yourself occurred to you?<br><br>10. Fo waati doo ka soto i k'e mira pur ka kuu jawo ke i fang na?	☐3.<br><br>Yes, quite often<br><br>Haa, sinyaa jamaa	☐2.<br><br>Sometimes<br><br>Haa, waati doolu	☐1.<br><br>Hardly ever<br><br>Haani, a mang siyaa fereng	☐0.<br><br>Never<br><br>Haani, a mang soto

For peer review only

EPDS
Wolof

Please tick the answer which comes closest to how you have felt *in the past week,* not just how you feel today. There are no right or wrong answers and please feel free to answer honestly as these questionnaires are confidential.

Kom nu nga neeke geugeni birr ne wala musa ham doom, dange bugga ham sa yeek-yeek. Ndimbaleh nyu nga hol tontu bu ngena jageh si sa yeek-yeek si birr bess bu ai bu passeh. Kom neekut le ngai yeek tai.

1. Have you been able to laugh and see the funny side of things?<br><br>1. Ndakh yaw dang nga deh muna rreh sor gisseh lula rretanlo?	☐0.<br><br>As much as I always could<br><br>Waaw, lu si baari kom nu ma ko muneh	☐1.<br><br>Not quite so much now<br><br>Waaw, neekut lu bareh kom nonu leegue	☐2.<br><br>Definitely not so much now<br><br>Degga-degga neekut lu bareh sah leegue	☐3.<br><br>Not at all<br><br>Amut sah
2. Have you looked forward with enjoyment to things?<br><br>2. Ndakh yaw dang deh muna saintu yo hamneh munnala kontanlo?	☐0.<br><br>As much as I ever did<br><br>Waaw, kom numa ko musa deffeh	☐1.<br><br>Rather less than I used to<br><br>Waaw, lu meelut kom nu ma ko dan deffeh	☐2.<br><br>Definitely less than I used to<br><br>Degga-degga neekut kom numa kodan deffeh	☐3.<br><br>Hardly at all<br><br>Waaw, su ameh sah barewut

For peer review only

3. Have you blamed yourself unnecessarily when things went wrong?<br><br>3. Ndakh yaw dang deh am sikka nge si sa boppa su mbirr dameh nom waruta demeh?	☐3.<br><br>Yes, most of the time<br><br>Waaw, si wakhtu yu bareh	☐2.<br><br>Yes, some of the time<br><br>Waaw, si yeena sayee	☐1.<br><br>Not very often<br><br>Neekut lu bareh	☐0.<br><br>No, never<br><br>Dadet, musuta ham
4. Have you been anxious or worried for no good reason?<br><br>4. Ndakh dang de ham jahleh walla hel bu dalut si li ko jarut?	☐0.<br><br>No, not at all<br><br>Dadet musuta ham	☐1.<br><br>Hardly ever<br><br>Su ameh yet bareh wut	☐2.<br><br>Yes, sometimes<br><br>Waaw, yeena sayee	☐3.<br><br>Yes, very often<br><br>Waaw, si wahktu yu bareh
5. Have you felt scared or panicky for no good reason?<br><br>5. Ndakh danga de ham ragal walla titangeh si lu ko jarut?	☐3.<br><br>Yes, quite a lot<br><br>Waaw, si wakhtu yu bareh	☐2.<br><br>Yes, sometimes<br><br>Waaw, yeena sayee	☐1.<br><br>No, not much<br><br>Dadet, barehwut	☐0.<br><br>No, not at all<br><br>Dadet, musuta ham sah

6. Have things been getting on top of you?<br><br>6. Ndakh Jeffeh-jeffeh daffa bareh si sa boopa bi be-nga hamneh anta nulo ko?	☐3.<br><br>Yes, most of the time I haven't been able to cope at all<br><br>Waaw, yeena sayee duma henu jeffeh-jeffeh yoyu	☐2.<br><br>Yes, sometimes I haven't been coping as well as usual<br><br>Waaw, yeena sayee duma deh muna henu jeffeh-jeffeh yoyu kom num wara demeh	☐1.<br><br>No, most of the time I have coped quite well<br><br>Dadet, yeena sayee dama deh muna henu jeffeh- jeffeh yoyu	☐0.<br><br>No, I have been coping as well as ever<br><br>Dadet, dama deh henu jeffeh-jeffeh yoyu kom nom musa mel
7. Have you been so unhappy that you have had difficulty sleeping?<br><br>7. Ndakh danga de ham nyaka kontante (walla nakhar) be takh dor deh muna nelew?	☐3.<br><br>Yes, most of the time<br><br>Waaw, wakhtu bu neeka	☐2.<br><br>Yes, sometimes<br><br>Waaw, yeena sayee	☐1.<br><br>Not very often<br><br>Neekut lu bareh	☐0.<br><br>No, not at all<br><br>Dadet, musuta ham sah
8. Have you felt sad or miserable?<br><br>8. Ndakh danga de yeek nakhar walla chono bo hamneh daffa doi war?	☐3.<br><br>Yes, most of the time<br><br>Waaw, wakhtu yu bareh	☐2.<br><br>Yes, quite often<br><br>Waaw, yeena sayee	☐1.<br><br>Not very often<br><br>Neekut lu bareh	☐0.<br><br>No, not at all<br><br>Dadet, musuta ham sah

For peer review only

9. Have you been so unhappy that you have been crying?<br><br>9. Ndakh dang de ham nekhar bula deh joi lo?	☐3.<br><br>Yes, most of the time<br><br>Waaw, wakhtu yu bareh	☐2.<br><br>Yes, quite often<br><br>Waaw, yeena sayee	☐1.<br><br>Only occasionally<br><br>Lu tuti si ai hew-hew	☐0.<br><br>No, never<br><br>Dadet, musuta ham
10. Has the thought of harming yourself occurred to you?<br><br>10. Ndakh am halat purr lurr sa boopa daff deh nyaw sisa hell bi?	☐3.<br><br>Yes, quite often<br><br>Waaw, si wahktu yu bareh	☐2.<br><br>Sometimes<br><br>Yeena sayee	☐1.<br><br>Hardly ever<br><br>Suma amet het barewut	☐0.<br><br>Never<br><br>Musuta ham

For peer review only

Supplementary Material 3

CHIME session Check-List

1. Did any of the songs address any of the following topics? Y/N (Circle which ones below)
    a. Importance of the singing group in supporting each other
    b. Importance of other positive relationships in their lives
    c. Resilience to challenges and empowerment
    d. Importance of being open, removing stigma to discuss challenges

2. Did the sessions dwell on the challenges of pregnancy or more the coping mechanisms and resilience? Y/N

3. Did the kanyeleng group incorporate a lullaby? If so, which? Y/N
    a. Lullaby name:
4. Did the kanyeleng group explain the benefits of singing lullabies? Y/N

5. Did the kanyeleng group use a welcoming song? If so, which? Y/N
    a. Song name:

6. Did the kanyeleng group use a closing song at the end? If so, which song? Y/N
    a. Song name:

7. Overall, did the participants actively participate in the session by joining in with the singing, dancing or clapping? Y/N

8. Were there any examples where the kanyeleng group acted to encourage women to participate? Y/N
    a. Give example and time point in the video:

For peer review only

Supplementary Material 4

Retention and Attrition Rates Overall and by Group

	Retention			Attrition		
	**Pre** *n* (%)	**Post** *n* (%)	**Follow-up** *n* (%)	**Pre-Post** (%)	**Post-Follow-up** (%)	**Pre-Follow-up** (%)
**All**	124 (100%)	99 (80%)	83 (67%)	20%	16%	33%
**CHIME**	50 (100%)	39 (78%)	33 (66%)	22%	15%	34%
**Control**	74 (100%)	60 (81%)	50 (68%)	19%	17%	32%

For peer review only

Supplementary Material 5

CHIME Session Duration

	Session Duration						
**Site**	**Session 1**	**Session 2**	**Session 3**	**Session 4**	**Session 5**	**Session 6**	**Average**
Gunjur	31.00	45.00	48.00	60.00	57.00	50.00	48.50
Kuntair	42.00	75.00	75.00	70.00	66.00	71.00	66.50
Pirang	75.00	50.00	65.00	55.00	50.00	65.00	60.00
Sukuta	55.00	78.00	64.00	61.00	60.00	60.00	63.00
**Total**	**50.75**	**62.00**	**63.00**	**61.50**	**58.25**	**61.50**	**59.50**

Note. Time is in minutes.

Number of participants Present at Each Session

	Session Attendance						
**Site**	**Total Possible**	**Session 1**	**Session 2**	**Session 3**	**Session 4**	**Session 5**	**Session 6**
Gunjur	11	9	6	7	8	7	8
Kuntair	11	11	10	8	11	11	11
Pirang	16	13	12	11	10	12	11
Sukuta	13	10	5	7	4	4	6

Note. Total Possible is the total amount of women recruited to be in the intervention.

For peer review only

Supplementary Material 6

EPDS and SRQ-20 Scores at Three Time-Points

**EPDS**		**Baseline**		**Post**		**Follow-up**
	n	***M(SD)***	***n***	***M(SD)***	***n***	***M(SD)***
All	124	4.25 (4.10)	99	2.56 (2.74)	83	1.80 (2.23)
CHIME	50	2.90 (3.07)	39	1.18 (1.50)	33	0.94 (1.73)
Control	74	5.16 (4.46)	60	3.45 (2.99)	50	2.36 (2.36)
**SRQ-20**		**Baseline**		**Post**		**Follow-up**
	n	***M(SD)***	***n***	***M(SD)***	***n***	***M(SD)***
All	124	7.27 (4.00)	99	5.31 (4.02)	83	4.25 (3.32)
CHIME	50	6.22 (3.83)	39	3.62 (3.32)	33	2.70 (3.03)
Control	74	7.97 (3.99)	60	6.42 (4.08)	50	5.28 (3.12)

For peer review only

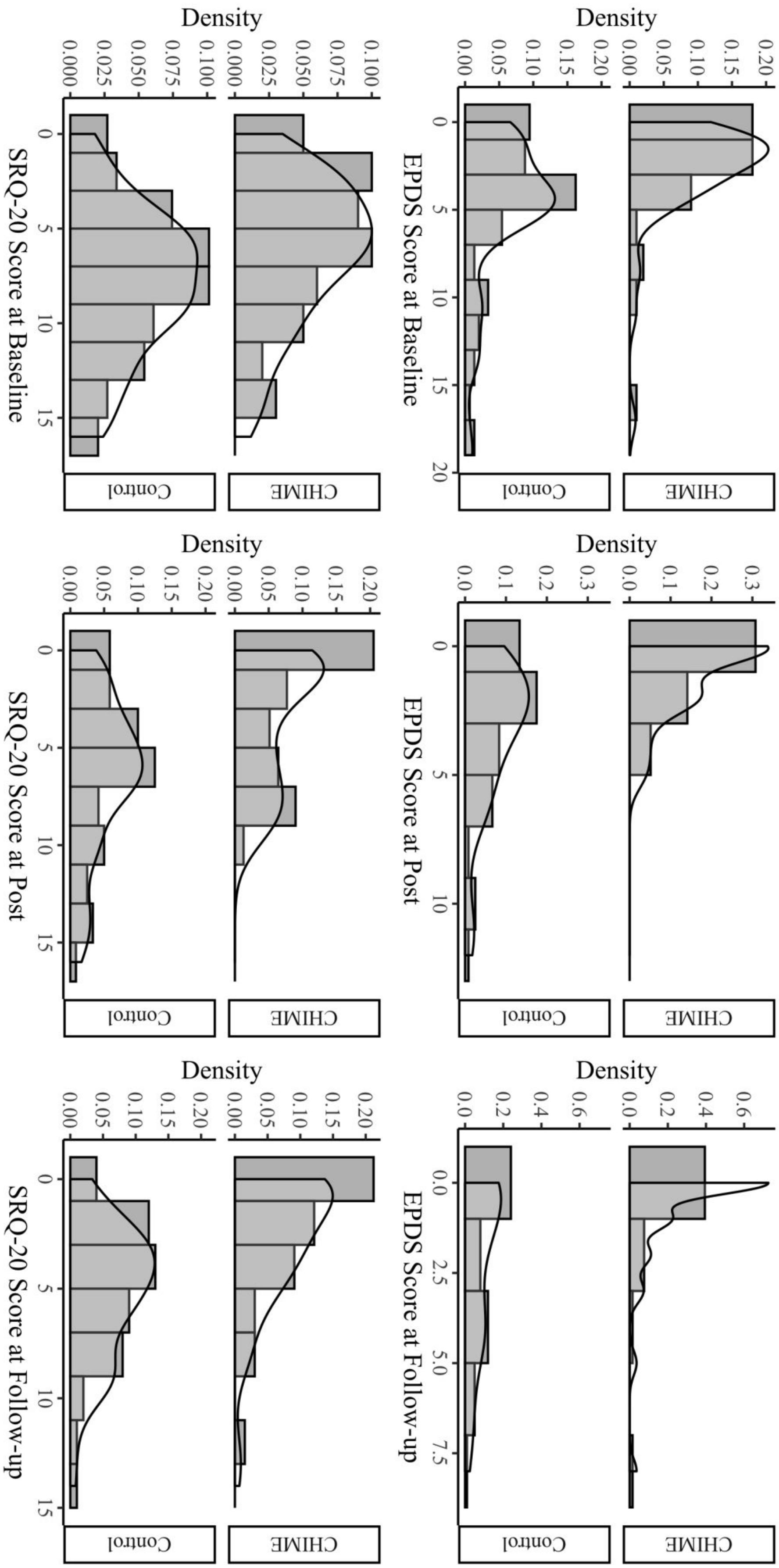
Density
SRQ-20 Score at Baseline
EPDS Score at Baseline
SRQ-20 Score at Post
EPDS Score at Post
SRQ-20 Score at Follow-up
EPDS Score at Follow-up
Control
CHIME

CONSORT 2010 checklist of information to include when reporting a pilot or feasibility trial*

Section/Topic	Item No	Checklist item	Reported on page No
**Title and abstract**			
	1a	Identification as a pilot or feasibility randomised trial in the title	Pg. 1
	1b	Structured summary of pilot trial design, methods, results, and conclusions (for specific guidance see CONSORT abstract extension for pilot trials)	Pg. 2
**Introduction**			
Background and objectives	2a	Scientific background and explanation of rationale for future definitive trial, and reasons for randomised pilot trial	Pgs. 4-6
	2b	Specific objectives or research questions for pilot trial	Pg. 7
**Methods**			
Trial design	3a	Description of pilot trial design (such as parallel, factorial) including allocation ratio	Pg. 7
	3b	Important changes to methods after pilot trial commencement (such as eligibility criteria), with reasons	NA
Participants	4a	Eligibility criteria for participants	Pg. 8
	4b	Settings and locations where the data were collected	Pg. 9
	4c	How participants were identified and consented	Pg. 8
Interventions	5	The interventions for each group with sufficient details to allow replication, including how and when they were actually administered	Pgs. 9-10
Outcomes	6a	Completely defined prespecified assessments or measurements to address each pilot trial objective specified in 2b, including how and when they were assessed	Pgs. 11-12
	6b	Any changes to pilot trial assessments or measurements after the pilot trial commenced, with reasons	NA
	6c	If applicable, prespecified criteria used to judge whether, or how, to proceed with future definitive trial	NA
Sample size	7a	Rationale for numbers in the pilot trial	Pg. 9.
	7b	When applicable, explanation of any interim analyses and stopping guidelines	NA
Randomisation:			Pgs. 7-8
Sequence generation	8a	Method used to generate the random allocation sequence	Pg. 7
	8b	Type of randomisation(s); details of any restriction (such as blocking and block size)	Pg. 7
Allocation concealment mechanism	9	Mechanism used to implement the random allocation sequence (such as sequentially numbered containers), describing any steps taken to conceal the sequence until interventions were assigned	Pg. 7

For peer review only

Implementation	10	Who generated the random allocation sequence, who enrolled participants, and who assigned participants to interventions	Pg. 7
Blinding	11a	If done, who was blinded after assignment to interventions (for example, participants, care providers, those assessing outcomes) and how	NA. Not blinded study.
	11b	If relevant, description of the similarity of interventions	NA
Statistical methods	12	Methods used to address each pilot trial objective whether qualitative or quantitative	Pgs. 13-14
**Results**			
Participant flow (a diagram is strongly recommended)	13a	For each group, the numbers of participants who were approached and/or assessed for eligibility, randomly assigned, received intended treatment, and were assessed for each objective	Pg. 16
	13b	For each group, losses and exclusions after randomisation, together with reasons	Pg. 16
Recruitment	14a	Dates defining the periods of recruitment and follow-up	
	14b	Why the pilot trial ended or was stopped	NA
Baseline data	15	A table showing baseline demographic and clinical characteristics for each group	Pg. 12
Numbers analysed	16	For each objective, number of participants (denominator) included in each analysis. If relevant, these numbers should be by randomised group	Pgs. 21-22
Outcomes and estimation	17	For each objective, results including expressions of uncertainty (such as 95% confidence interval) for any estimates. If relevant, these results should be by randomised group	Pgs. 21-22
Ancillary analyses	18	Results of any other analyses performed that could be used to inform the future definitive trial	Pgs. 14-22
Harms	19	All important harms or unintended effects in each group (for specific guidance see CONSORT for harms)	Pg. 21
	19a	If relevant, other important unintended consequences	NA
**Discussion**			
Limitations	20	Pilot trial limitations, addressing sources of potential bias and remaining uncertainty about feasibility	Pgs. 25-26
Generalisability	21	Generalisability (applicability) of pilot trial methods and findings to future definitive trial and other studies	Pgs. 22-25
Interpretation	22	Interpretation consistent with pilot trial objectives and findings, balancing potential benefits and harms, and considering other relevant evidence	Pgs. 22-25
	22a	Implications for progression from pilot to future definitive trial, including any proposed amendments	Pgs. 22-25
**Other information**			
Registration	23	Registration number for pilot trial and name of trial registry	Pg. 2
Protocol	24	Where the pilot trial protocol can be accessed, if available	Pg. 7
Funding	25	Sources of funding and other support (such as supply of drugs), role of funders	Pg. 28
	26	Ethical approval or approval by research review committee, confirmed with reference number	Pg. 28

For peer review only

BMJ Open

A community psychosocial music intervention to reduce antenatal common mental disorder symptoms in The Gambia: A feasibility study

Journal:	*BMJ Open*
Manuscript ID	bmjopen-2020-040287.R1
Article Type:	Original research
Date Submitted by the Author:	20-Aug-2020
Complete List of Authors:	Sanfilippo, Katie Rose; Goldsmiths University of London, Psychology<br>McConnell, Bonnie ; Australian National University<br>Cornelius, Victoria; Imperial College London, Clinical Trials Unit<br>Darboe, Buba; Ministry of Health and Social Welfare The Gambia<br>Huma, Hajara; Ministry of Health and Social Welfare The Gambia; National Centre for Arts and Culture The Gambia<br>Gaye, Malick ; Ministry of Health and Social Welfare The Gambia; National Centre for Arts and Culture The Gambia<br>Ceesay, Hassoum ; National Centre for Arts and Culture<br>Ramchandani, Paul; University of Cambridge<br>Cross, Ian; Cambridge University<br>Glover, Vivette; Imperial College London<br>Stewart, Lauren ; Goldsmiths University of London
**Primary Subject Heading**:	Mental health
Secondary Subject Heading:	Global health, Obstetrics and gynaecology
Keywords:	Maternal medicine < OBSTETRICS, Anxiety disorders < PSYCHIATRY, Depression & mood disorders < PSYCHIATRY, PUBLIC HEALTH

SCHOLARONE™
Manuscripts

I, the Submitting Author has the right to grant and does grant on behalf of all authors of the Work (as defined in the below author licence), an exclusive licence and/or a non-exclusive licence for contributions from authors who are: i) UK Crown employees; ii) where BMJ has agreed a CC-BY licence shall apply, and/or iii) in accordance with the terms applicable for US Federal Government officers or employees acting as part of their official duties; on a worldwide, perpetual, irrevocable, royalty-free basis to BMJ Publishing Group Ltd ("BMJ") its licensees and where the relevant Journal is co-owned by BMJ to the co-owners of the Journal, to publish the Work in this journal and any other BMJ products and to exploit all rights, as set out in our licence.

The Submitting Author accepts and understands that any supply made under these terms is made by BMJ to the Submitting Author unless you are acting as an employee on behalf of your employer or a postgraduate student of an affiliated institution which is paying any applicable article publishing charge ("APC") for Open Access articles. Where the Submitting Author wishes to make the Work available on an Open Access basis (and intends to pay the relevant APC), the terms of reuse of such Open Access shall be governed by a Creative Commons licence – details of these licences and which Creative Commons licence will apply to this Work are set out in our licence referred to above.

Other than as permitted in any relevant BMJ Author's Self Archiving Policies, I confirm this Work has not been accepted for publication elsewhere, is not being considered for publication elsewhere and does not duplicate material already published. I confirm all authors consent to publication of this Work and authorise the granting of this licence.

For peer review only

A community psychosocial music intervention to reduce antenatal common mental disorder symptoms in The Gambia: A feasibility study

Katie Rose M. Sanfilippo[*1], Bonnie B. McConnell[#2], Victoria Cornelius[†3], Buba Darboe[⊥4], Hajara B. Huma[§⊥5], Malick Gaye [§⊥6], Hassoum Ceesay[7], Paul Ramchandani[‡8], Ian Cross[‡9], Vivette Glover[†10], Lauren Stewart[*11]

[*] *Psychology Department, Goldsmiths, University of London, UK*

[#] *The Australian National University, Australia*

[†] *Imperial College London, UK*

[‡] *University of Cambridge, UK*

[§] *The National Centre for Arts and Culture, The Gambia*

[⊥] *The Ministry of Health and Social Welfare, The Gambia*

[1]ksanf001@gold.ac.uk, [2]Bree.McConnell@anu.edu.au, [3]v.cornelius@imperial.ac.uk, [4]darboejula@hotmail.com, [5]hajarahuma24@gmail.com, [6]malickgaye60@yahoo.com, [7]hceesay@gmail.com, [8]pr441@cam.ac.uk, [9]v.glover@imperial.ac.uk, [10]ic108@cam.ac.uk, [11]l.stewart@gold.ac.uk

Correspondence concerning this paper should be addressed to Katie Rose M. Sanfilippo

Address: Whitehead Building, Psychology Department, Goldsmiths, University of London, SE14 6NW, UK.

E-mail: ksanf001@gold.ac.uk

Word count: 4,565

For peer review only - http://bmjopen.bmj.com/site/about/guidelines.xhtml

ABSTRACT

Objectives: Examine the feasibility of a Community Health Intervention through Musical Engagement (CHIME) in The Gambia to reduce common mental disorder (CMD) symptoms in pregnant women.

Design: Feasibility trial testing a randomised stepped wedge cluster design.

Setting: Four local antenatal clinics.

Participants Women who were 14 – 24 weeks pregnant and spoke Mandinka or Wolof were recruited into the intervention (n = 50) or control group (n = 74).

Intervention: Music-based psychosocial support sessions designed and delivered by all-female fertility societies. Sessions lasted one hour and were weekly for six weeks. Delivered to groups of women with no preselection. Sessions were designed to lift mood, build social connection and provide health messaging through participatory music making. The control group received standard antenatal care.

Outcomes: Demographic, feasibility, acceptability outcomes, and the appropriateness of the study design were assessed. Translated measurement tools (Self-Reporting Questionnaire (SRQ-20); Edinburgh Postnatal Depression Scale (EPDS)) were used to assess CMD symptoms at baseline, post-intervention and 4-weeks follow-up.

Results: All clinics and 82% of women approached consented to take part. A 33% attrition rate across all time-points was observed. 72% in the intervention group attended at least three sessions. Audio and video analysis confirmed fidelity of the intervention and a thematic analysis of participant interviews demonstrated acceptability and positive evaluation. Results showed a potential beneficial effect with a reduction of 2.13 points (95%CI [0.89, 3.38], $p < 0.01$, $n = 99$) on the SRQ-20 and 1.98 points (95% CI [1.06, 2.90],

For peer review only - http://bmjopen.bmj.com/site/about/guidelines.xhtml

$p < 0.01$, $n = 99$) on the EPDS at the post-intervention timepoint for the intervention group compared to standard care.

Conclusion: Results demonstrate that CHIME is acceptable and feasible in The Gambia. To our knowledge, CHIME is the first example of a music-based psychosocial intervention to be applied to perinatal mental health in a LIMC context.

Trial Registration: Pan African Clinical Trials Registry (PACTR) (24/01/2019): PACTR201901917619299

ARTICLE SUMMARY

Strengths and limitations of the study

- We used a randomised stepped wedge cluster trial design to conduct a feasibility study of a psychosocial, music-based intervention for women's perinatal mental health.
- The intervention was co-developed with all female fertility societies (Kanyeleng groups) in The Gambia.
- There was broad community and government involvement (Ministry of Health and Social Welfare) throughout the development of the intervention and research.
- The intervention was delivered at clinic level and women were eligible to participate regardless of CMD symptom scores.
- Participants were unable to be blinded to which group they were assigned, increasing the potential for response bias.

For peer review only

BACKGROUND

Perinatal common mental health disorders (CMDs) affect up to one in five women worldwide and in low- and middle-income countries (LMICs) they can be twice as frequent as in high income countries (HICs) [1]. The World Health Organization (WHO) International Classification of Disease (ICD-10) defines CMDs as “mood disorders’ and ‘neurotic, stress-related and somato-form disorders” which include depressive and anxiety disorders [2]. Perinatal mental distress is of global concern as mental health problems such as stress, anxiety and depression have been shown to have a negative impact on the mother, her developing infant, and their relationship [3–5]. Antenatal anxiety and depression strongly predict postnatal depression [6,7] and can negatively impact fetal and infant development [8,9]. Hence, antenatal mental health interventions are important in order to improve outcomes for both the mother and child. In LMICs, there is often a scarcity of psychologists and psychiatrists able to treat affected women. Therefore, it is necessary to develop effective, low-cost, non-stigmatising and culturally appropriate interventions to support women's mental health during the perinatal period [10–12].

The majority of previous research in community mental health and perinatal mental health interventions in LMICs focus on educational and psychosocial interventions primarily delivered via conversations and social gatherings. The possibility of using participatory music making as the vehicle to deliver a perinatal mental health intervention has not been studied within a LMIC context. Research in HICs has shown that singing in groups can be a powerful modulator of mood and emotion, reducing symptoms of anxiety and depression

For peer review only - http://bmjopen.bmj.com/site/about/guidelines.xhtml

[13], including during pregnancy [14–16], and increasing well-being, social affiliation, group bonding and participants' mood [17–19]. In LMICs, songs have shown to be a helpful and common medium to convey specific health messages, especially in areas where literacy rates are low [20–22]. These principles formed the basis for the developed intervention.

The current project took place within The Gambia, a small country on the West African coast. With a population of about two million, The Gambia is the most densely populated country in West Africa [23]. There are five major ethnic groups in The Gambia, each with their own language and culture. Mandinka is the most common (42%) followed by Fula (18%), Wolof (16%), Jola (10%), Serahuli (9%) and smaller ethnic groups and foreigners comprising the remainder [24]. A large majority of households (91%) have access to an improved source of drinking water, and 45% of households have access to electricity, however, there is a large disparity between urban and rural areas (66% and 13%, respectively) [25]. Only 37% of households use improved toilet facilities that hygienically separate human excreta from human contact and are not shared with other households [25]. The World Bank estimates a $31 per capita health expenditure per annum, one of the lowest in the world [26]. Mental health services and specifically services focused on perinatal mental health are minimal or non-existent [25]. There are also high levels of stigma associated with CMDs that impede women's ability to recognise symptoms and seek help and support.

For peer review only - http://bmjopen.bmj.com/site/about/guidelines.xhtml

The Gambia is a medically pluralistic society where people utilize a variety of therapeutic options, including biomedicine, indigenous herbal medicines, and spiritual treatments [27]. A music-centred approach for perinatal mental health may be particularly fruitful in The Gambia as there are many different existing music practices that involve health communication, pregnant women and new mothers. Kanyeleng groups, or fertility societies, sing together and perform ceremonies that promote fertility and support women during pregnancy and throughout motherhood [28]. They also work with the Ministry of Health and Social Welfare to promote health initiatives through performance [20]. The intervention examined here was co-developed with local Kanyeleng groups with the aim of having women leave the sessions in a positive mood, feeling part of a socially-supportive group, with some new strategies of how to cope with some of the common physical and psychological challenges of pregnancy and equipped with skills to seek wider social support outside the sessions.

Objectives

The main aim of this study was to test the feasibility of a Community Health Intervention through Musical Engagement (CHIME) to help reduce CMD symptoms in pregnant women compared with standard care. The study had five objectives:

1. To obtain demographic information on the eligible population
2. To determine if our measurement tools, the Edinburgh Postnatal Depression Scale (EPDS) and the Self-Reporting Questionnaire (SRQ-20), are useable.
3. To determine if the intervention is deliverable.

For peer review only - http://bmjopen.bmj.com/site/about/guidelines.xhtml

4. To determine if the stepped wedge trial design is deliverable and obtain information that will inform the definitive study.
5. To determine if this type of intervention is culturally appropriate and well received by the community and health workers.

METHODS

The trial protocol has been published and contains more detailed information about the intervention development and methods [29].

Trial design

The randomised stepped wedge cluster trial design had two steps with 4 clusters (antenatal clinics). All clinics undertook the control group period prior to the intervention group period. The two clinics randomised to sequence 1 undertook these immediately, the two clinics randomised to sequence 2 undertook their control groups 12 weeks later. Randomisation was undertaken after clinic consent to take part in the trial was obtained. The study's statistical advisor generated a computer randomisation list with a block size of 2, which was applied to the pre-supplied clinic list by the trial team. Separate cohorts of participants were recruited for each period to the control group and the intervention group, ensuring all participants began the study 4 – 6 months through their pregnancy. The 12-week phase for both the control and intervention group included data collection at week 1 ('baseline') and week 7 ('post-intervention') after either participating in the intervention sessions (CHIME) or standard care (Control). Data was also collected at week 11 ('follow-up'), four weeks after the intervention finished. This study is reported

For peer review only

For peer review only - http://bmjopen.bmj.com/site/about/guidelines.xhtml

in accordance with the Consolidated Standards of Reporting Trials 2010 statement: extension to randomised pilot and feasibility trials [30].

Clinic inclusion

Sites were in The Gambia, spanned a range of locations and ethnic groups, including rural and urban as well as Wolof- and Mandinka-speaking areas. Each clinic also had an active local Kanyeleng group who could deliver the intervention.

Participants

Participants were not preselected based on their mental health symptoms. All participants who were attending the consented sites during the active study period and who were 18 or older, spoke either Mandinka or Wolof fluently, and were 14 – 24 weeks pregnant were invited to take part in the study. Women with a history of a previous late-term miscarriage or those who had current or a history of psychosis were excluded. Other ethical considerations are given within the protocol paper [29].

Recruitment

Participants were recruited during antenatal clinic days. All data was collected at the antenatal clinics. The researchers and participants were not blinded to whether they were in the intervention or control group. All participants were offered a total of 600 Dalasi (about 12 USD) for their time, 200 (about 4 USD) at each data collection time point. They were reminded of the data collection and the intervention sessions by a phone call. They were called three days before, one day before and on the day of data collection.

For peer review only - http://bmjopen.bmj.com/site/about/guidelines.xhtml

Sample Size

The size of the study was based on its ability to inform the design of a future definitive study. We aimed to collect data from 120 pregnant women, 60 controls and 60 in the intervention. This number was sufficient to provide an estimate of a binary feasibility outcome (e.g. recruitment rate) within at least ±9 percentage points for the 95% confidence interval and provide reasonable estimates of the standard deviation [31]. As the intervention delivery was thought to be possible within groups of approximately 15, the aim was to include 4 clinics with one control and one intervention group per clinic.

Intervention

The intervention was psychosocial and musical in nature, with sessions centring on group singing and led by the Kanyeleng group local to each antenatal clinic. Each Kanyeleng group is comprised of approximately 10 women. The nature of the intervention was necessarily contextualized across the four settings, especially as Wolof- and Mandinka-speaking groups have distinct cultural beliefs, practices and language. A training workshop held with the Kanyeleng groups before the intervention began ensured that the overarching goals, content and approach to session delivery could be broadly standardized. Key messages incorporated within the session songs included: a) common physical and psychological symptoms of pregnancy, b) techniques to cope with and manage these with an emphasis on the importance of the singing group supporting each other, other positive relationships in their lives, resilience to challenges and empowerment, and the importance of being opening and removing stigma to discuss challenges, and c) select messages on

For peer review only - http://bmjopen.bmj.com/site/about/guidelines.xhtml

childcare. The document used to aid the discussions during the intervention development workshops can be found in Supplementary Material 1.

Participants in the CHIME intervention attended six 60-minute music sessions held at their local antenatal clinic in addition to receiving standard antenatal care. During each session, the Kanyeleng groups introduced specific songs drawing on traditional repertoire but adapting them to include new lyrics focussed on the agreed messages. The participants were encouraged to join in by singing, moving to the music and clapping. Sessions involved call-and-response singing, with participants improvising along the themes described above. Each session began with a welcome song and ended with a closing song. One lullaby was also introduced at each session to give the women repertoire to draw on postpartum. A community health nurse (CHN) at each clinic was present to observe, take attendance data, and report any issues of concern to the research team including any potential adverse effects.

Each control group received standard antenatal clinic care without any additional intervention. Standard care consists of 4 or more regular visits to the antenatal clinic with little to no mental health care [25].

Outcomes

Two questionnaires were used to measure CMD symptoms. Both were translated into Mandinka and Wolof. The translation method was based on suggestions from the WHO

For peer review only - http://bmjopen.bmj.com/site/about/guidelines.xhtml

[32], Hanlon et al. [33] and Cox, Holden and Henshaw [34]. The translated versions can be found in Supplementary Material 2.

The Self-report Questionnaire (SRQ-20) [35] is a 20-item scale developed by the WHO to screen for psychiatric disturbance, especially in LMICs. Its items ask about anxiety, depression and somatoform symptoms. It has been used in various sub-Saharan African contexts to measure perinatal mental health [36] but has never been used in The Gambia.

The second measurement tool used was the Edinburgh Postnatal Depression Scale (EPDS), a ten-item scale that was developed to screen for postnatal depression [34] and has subsequently been validated to be used during pregnancy [37]. It has been validated for perinatal use in other African contexts [36,38], and used in The Gambia before, though a validated version could not be obtained [39–41].

Procedure

Four antenatal clinics in western Gambia were approached to participate in the feasibility trial. The head midwife gave consent via a paper consent form. There is a low female literacy rate within The Gambia, about 45% in 2015 [25]. Therefore, participants who met the inclusion criteria were read the information sheet and consent was given orally and verified via thumbprint or signature. All data were collected orally and recorded on paper Case Report Forms (CRFs).

For peer review only - http://bmjopen.bmj.com/site/about/guidelines.xhtml

At all three assessments (baseline, post-intervention, follow-up) EPDS and SRQ-20 scores were collected. Demographic data were collected at baseline and a semi-structured interview was conducted with each participant within the intervention group at the post-intervention assessment. Focus group discussions (FGDs) were conducted with each of the four Kanyeleng groups and the CHNs. The questions in the interviews and FGDs addressed the participants' overall thoughts about the intervention, whether or not they thought it had been helpful, their comments on the structure, and any suggestions for the future. All interviews and FGDs were audio-recorded, transcribed and translated.

Patient and Public Involvement

We engaged with patients and the public to inform the design of this trial, intervention development, acceptability and dissemination strategy. We also engaged extensively with relevant stakeholders to ensure optimal study design and acceptability of the intervention including a half day meeting in the Gambia, with delegates from the Ministry of Health and Social Welfare, the National Centre for Arts and Culture, a local obstetrician, patient advocate groups and students in Public Health and Psychiatric Nursing. The dissemination of these findings will include open field days in the local community (mini festivals with speeches and musical performances) allowing us to share the results from this trial and obtain feedback from the community.

Statistical Methods

For peer review only

For peer review only - http://bmjopen.bmj.com/site/about/guidelines.xhtml

Analysis was conducted using the intention-to-treat principle [42]. All eligible participants were analysed in the group to which they were randomized regardless of adherence to the intervention.

Descriptive statistics were used to summarise demographic variables of the recruited population. Plots were used, and skewness calculated, to assess the distribution of the outcome measurements across the three assessment time-points and by each group. To determine if the intervention was deliverable, the number of sessions the Kanyeleng delivered, the duration of each session, and the intervention fidelity at the four sites was recorded. To measure the fidelity of the intervention, both RAs watched audio-visual recordings of the first and fourth intervention sessions from each clinic. They then completed a checklist (Supplementary Material 3) to determine if all the necessary elements, as outlined in the training workshops, had been included in the intervention. Proportions of specific agreement were calculated to assess the reliability of the fidelity measure. The proportion of approached clinics that gave consent was calculated, and any scheduling problems in keeping with the stepped wedge timeline were recorded. Recruitment and adherence rates were calculated for both groups.

The intervention effect was estimated using mixed effect linear regression models. Two regression models were created to estimate the between-group mean difference in SRQ-20 and EPDS score 1 week after completion of the intervention (post-intervention) and 4 weeks later (follow-up). Each model was adjusted for baseline scores, clinic and included

For peer review only

a time by group interaction with participant as a random effect and unstructured covariance. The mean differences were presented with 95% confidence intervals.

For missing values on the SRQ-20 or EPDS individual items, multiple imputation was performed using a predictive mean matching method using the *MICE* package in R [43]. The model included all quantitative demographic information collected, including age, gestational age, parity and gravida. If a participant's data was not able to be collected at one of the post-intervention assessments (post-intervention or follow-up) due to attrition it was treated as missing.

All statistical analysis was run using R [44]. A thematic analysis [45] of the post-intervention interviews and FGDs with the participants, Kanyeleng groups and CHNs was performed using Dedoose [46].

RESULTS

Demographic information

Table 1 shows the demographic information of the entire sample and by group. Participants were between the ages of 18 and 40 (M = 26.95, SD = 5.72) and between 14 and 24 weeks pregnant (M = 20.81, SD = 3.32). It can be seen in Table 1 that the distribution of the participant characteristics are similar between the two groups.

Table 1. Demographic data in total and by group

For peer review only - http://bmjopen.bmj.com/site/about/guidelines.xhtml

	All Women (*n* = 124) *M*(SD)	Intervention Group (*n*= 50) *M*(SD)	Control Group (*n*= 74) *M*(SD)
**Age**	26.95 (5.72)	26.82 (5.59)	27.04 (5.85)
**Gestational Age**	20.81 (3.32)	21.14 (3.26)	20.58 (3.36)
**Gravida**	3.68 (2.19)	3.74 (2.32)	3.65 (2.11)
	***n*(% of 124)**	***n*(% of 50)**	***n*(% of 74)**
**Parity**			
Primiparous	27 (22%)	11 (22%)	16 (22%)
Multiparous	97 (78%)	39 (78%)	58 (78%)
**Marital Status**			
Single/Divorced/Separated/Widowed	5 (4%)	1 (2%)	4 (5%)
Married (Monogamous)	83 (67%)	37 (74%)	46 (62%)
Married (Polygamous)	36 (29%)	12 (24%)	24 (33%)
**Education Level**			
None	5 (4%)	3 (6%)	2 (3%)
Informal (Arabic)	62 (50%)	28 (56%)	34 (46%)
Primary	19 (15%)	7 (14%)	12 (16%)
Secondary/Tertiary	38 (31%)	12 (24%)	26 (35%)
**Interview Language**			
Mandinka	97 (78%)	40 (80%)	57 (77%)
Wolof	27 (22%)	10 (20%)	17 (23%)
**Participant's Occupation**			
Housewife	72 (58%)	32 (64%)	40 (54%)
Other	52 (42%)	18 (36%)	34 (46%)
**Husband's Occupation**			
Skilled Work	47 (38%)	22 (44%)	25 (34%)
Manual/Trade Work	77 (62%)	28 (56%)	49 (66%)

Note. Of those in a polygamous marriage 12 were the first wife, 21 were the second wife and 4 were the third wife. Demographic categories are based on those used by the Ministry of Health and Social Welfare.

Retention and Timeline adherence

For peer review only

Four antenatal clinics were approached to take part: Pirang Health Centre, Gunjur Health Centre, Sukuta Health Centre and Kuntair Health Centre. All clinics that were approached agreed to participate. Of the 152 women approached, 124 (81.6%) consented and were included in the study and the analysis. 20% declined from the Control group, and 12% declined from the intervention group. Figure 1 displays the participant flow diagram, indicating the number of participants included at each stage of the study.

Figure 1. CONSORT participant flow diagram. The light blue indicates the Control group and the dark blue indicates the intervention group. The red boxes outline the two 12-week sequences.

Attrition was 20% between baseline and post-intervention assessment, and 33% across all three assessment time-points. There was no difference in attrition between the groups. The retention and attrition rates across all three data collection time-points and by group are displayed in Supplementary Material 4.

The timeline of the stepped wedge schedule could not be strictly adhered to. Figure 2 shows the planned versus the actual timeline of the trial. About 96% of people in the Gambia are Muslim [25] and during Ramadan engaging in musical activities is prohibited. The timeline would have meant that some of the intervention sessions and the final data collection would take place during Ramadan. Therefore, the timeline shifted forward to ensure all data collection and intervention sessions could be completed before Ramadan. This shift meant that the intervention sessions started before the follow-up assessments were completed for

For peer review only

For peer review only - http://bmjopen.bmj.com/site/about/guidelines.xhtml

the control group. However, risk of contamination was small as the intervention sessions happened on non-clinics days when women in the control group were not present and all follow-up data collection for the control group was held on a different day to that of the intervention sessions.

Figure 2. Trial Timeline. The actual trial timeline compared to the planned timeline.

Intervention deliverability

All four Kanyeleng groups were able to deliver all six sessions across the six weeks. Sessions lasted 60 minutes on average. A detailed display of session duration as well as the number of participants in attendance per session per clinic can be found in Supplementary Material 5. 62% of participants attended at least 4 or more sessions across all the sites and 72% attended at least half (3 sessions). Only 6 participants (12%) did not attend any session. Kuntair had the highest attendance with 100% of participants attending at least 4 sessions. Pirang had the second highest attendance with 76% attending at least 4 sessions followed by Gunjur with 70%. Sukuta, the only clinic within an urban setting, had the lowest attendance overall with 31% of participants attending at least four sessions.

Based on the checklist, both RAs rated each video and found that all recorded sessions included all of the agreed elements. Agreement between the two RAs was 100%.

Acceptability of CHIME

For peer review only

For peer review only - http://bmjopen.bmj.com/site/about/guidelines.xhtml

36 participants within the intervention group were interviewed across the four clinics. Five FGDs were held, one with the CHNs and one with each of the four Kanyeleng groups. Five higher-level themes were developed: Learning, Peaceful Mind, Social Relationships, Suggestions for the Future, and Evaluations. Within these themes, different categories were created. See Table 2 for the coding structure used and an example except representing each category.

Table 2. Coding structure with examples

**High Level Theme**	**Category**	**Codes**	**Example**
**Learning**	Care for baby		"The pregnant women can understand how to take care of their children when they give birth" – Kanyeleng
	Coping		"What I learnt was as a pregnant woman don't isolate yourself to be free from lack of peace of mind. Mingle with the people and do everything together." – Pregnant Participant
	Health Information		"It is a taboo to discuss these type of things. So if this kind of programme is implemented a lot of doubts will be cleared in regards to pregnancy, delivery and so on. […] If programmes like this are coming we can talk to the antenatal mothers and I think is going to help a lot." – CHN
	Music		"A learned about a lot of songs." – Pregnant Participant
**Peaceful Mind**			"It makes me have peace of mind and makes me happy. When I leave here and go home I don't worry too much. That makes me very happy." – Pregnant Participant
	Continue outside session		"The songs that are sung here when I go home I remember them and that makes me happy. That alone is making me happy. I don't have distress anymore." – Pregnant Participant
**Social Relationships**			
	Outside Relationships		"If you look at the register itself, people were selected from different communities which I think is key. This information is very powerful. You cannot

For peer review only

			limit it to area because it is disseminated to everybody within that corner" – CHN
		Husband	"Within the midst in my area there is a woman even the husband invited me to explain her wife's problem to him"- Pregnant Participant
		Research Team	"Because you did not forget about us and you are now calling us for an interview. That alone shows that I am a member of the group" – Pregnant Participant
		Teaching others	"Right now I am helping someone and I am a teacher on my own" – Pregnant Participant
	Part of the singing group		"They make friends, they become used to the Kanyelengs, they were creating jokes they were telling them when you deliver we will come and attend your naming ceremony. […] They were used to each other even the day they were going, the last day I felt it" – CHN
**Suggestions for future**			
	Attendance and Participation		"The challenges are there, some of the challenges are before they gather is a problem and other scheduled programmes is also a problem. " – CHN
		Payment	"To encourage pregnant women to join the programme, give them more training and provide them some allowances to motivate them as some of them were complaining about it." – Pregnant Participant
		Transportation	"For somebody who also finds difficult to put food on the table, if you ask that individual even to come once in a week, find it very difficult as some of them have to go in for credit to pay fares to come. If at all something is going to be created for them, everyday you ask how much do they pay for the fare to come, then you give them that so that they […] will make coming to the session easier." – Kanyeleng
	Breakfast		"We work with pregnant women and they should eat well. They feel lazy when they don't eat. When food is provided, we can even have more hours for the programme. You can consider that for us. We can bear our hunger but for them they cannot" – Kanyeleng
	Continuation		"Can the programme be able to follow the antenatal from six months up to the delivery? When you start and stop within two months, maybe before the delivery, the woman can have stress. It is important to follow the woman until the delivery like from six

For peer review only - http://bmjopen.bmj.com/site/about/guidelines.xhtml

			months to the delivery so that you can see the impact." – CHN
	Timing		"If you look at these women most of them are business women. They do go to the market to sell, and some of them are alone in their houses so if you want to go beyond the one hour or forty five minutes is going to create a lot of problem" – CHN
**Evaluation**	Negative/ Neutral		"Sometimes transportation is problem" – Pregnant Participant
	Positive		"I am pleased about the programme, because I isolated myself before, and brought a lot thinking on my side. But when I took part in this programme I now go out and mingle with the people to have a chat and my mind has been steady now. " – Pregnant Participant
		Music and performance	"After the programme, my mind has changed because of the songs that were sang there. […] I was lonely when the programme ended. The performance of the Kanyeleng alone bring happiness." – Pregnant Participant
		Session Structure	"The way the songs are organized is good. When you meet with the people you have to greet each other, and when you meet you explain why they are called. When you are dispersing you disperse in peace. We all come together and entertain each other that is good." – Pregnant Participant

CHIME was evaluated positively, both by those who ran the sessions and those who participated. Participants thought that the information and strategies they learned in the sessions impacted the larger community. All thought that it was appropriate and the goals were achieved. The suggestions for the future that could be incorporated in a larger trial included extending the length of the intervention, reimbursing transportation costs and providing food. No harms, serious adverse effects or adverse effects were recorded or discussed.

For peer review only

For peer review only - http://bmjopen.bmj.com/site/about/guidelines.xhtml

Missing Data

A pronunciation problem by one RA on EPDS item 3 in Mandinka was detected during the baseline data collection for the first sequence. This affected the baseline EPDS scores and resulted in 46 missing values on item 3 of the EPDS. Multiple imputation was used to fill in the missing EPDS item 3 data at baseline and was used throughout subsequent analyses.

SRQ-20 and EPDS Baseline Scores

Table 3 displays the descriptive statistics of the SRQ-20 and EPDS scores at baseline. Supplementary Material 6 displays the mean, median and distribution of the SRQ-20 and EPDS scores at all three time-points. The SRQ-20 scores (M = 7.27, SD = 4.00) are approximately symmetric (skewness = 0.45) at baseline. The distribution of the EPDS (M = 4.25, SD = 4.10) scores at baseline is highly positively skewed (skewness = 1.48) with the majority of participants presenting few CMD symptoms. There was a significantly lower SRQ-20 baseline score (t (108.02) = 2.46, $p < 0.05$ 95% CIs[0.15, 3.56]) for the intervention group (M = 6.22, SD = 3.83) compared to the control group (M = 7.97, SD = 3.99), and a significantly lower EPDS baseline score (t(121.96) = 3.35, $p < 0.01$ 95%CIs[0.38, 2.91]) for the intervention group (M = 2.90, SD = 3.07) compared to the control group (M = 5.16, SD = 4.46).

For peer review only

Table 3. SRQ-20 and EPDS scores at Baseline

**SRQ-20**	**Baseline**		
	**n**	**Mean (SD)**	**Median (IQR)**
All	124	7.27 (4.00)	7 (4.75–10)
Intervention	50	6.22 (3.83)	6 (3–8.75)
Control	74	7.97 (3.99)	8 (5–10)

For peer review only - http://bmjopen.bmj.com/site/about/guidelines.xhtml

EPDS	Baseline		
	n	Mean (SD)	Median (IQR)
All	124	4.25 (4.10)	3 (1−5)
Intervention	50	2.90 (3.07)	2 (1−4)
Control	74	5.16 (4.46)	4 (2−6)

Indication of potential efficacy

At the post-intervention assessment there was a mean reduction of 2.13 points (95%CI [0.89, 3.38], $p < 0.01$, $n = 99$) on the SRQ-20 in the CHIME group compared to the control group and a mean reduction of 1.98 points (95% CI [1.06, 2.90], $p < 0.01$, $n = 99$) on the EPDS in the intervention group compared to the control group.

At the 4-week follow-up assessment a mean reduction of 2.09 points (95%CI [0.76, 3.42], $p < 0.01$, $n = 83$) on the SRQ-20 was found in the intervention group compared to the control group, and a mean reduction of 0.98 points (95%CI [-0.01, 1.97], $p = 0.05$, $n = 83$) on the EPDS was found in the intervention group compared to the control group. A table of these results can be found in Supplementary Material 7.

DISCUSSION

Overall, the trial was able to address our main objectives and show feasibility, acceptability, and a potential benefit of CHIME compared to standard care in The Gambia. High recruitment levels (81% of those approached consented) and reasonably low attrition rates (20% between baseline and post-intervention, 33% across all three time-points) were found. Feedback from participants and RAs revealed that some attrition could have been due to the women being difficult to contact, through a low phone battery or call credit. A

For peer review only - http://bmjopen.bmj.com/site/about/guidelines.xhtml

future study might help overcome this by providing either phones or phone credit to the participants.

Another way to encourage participant retention would be to implement some of the suggestions discussed in the post-intervention interviews and FGDs. Lack of funds for transportation was a barrier many participants discussed, together with concern that the pregnant women lacked the energy needed to participate in the music making because they were hungry. Traditional Kanyeleng ceremonies and gatherings usually include sharing food [47,48]. Including breakfast and payment for transportation might thus be a helpful addition to the programme. Finally, it was also suggested that CHIME continue longer into pregnancy, following the participants until delivery. Consideration of cost and sustainability would be needed to help address these suggestions [49]. This could be done through the involvement of policymakers through our partners at the Ministry of Health and Social Welfare in The Gambia and through a cost-effectiveness analysis.

As discussed, the timeline of the intervention needed to be shifted forward due to Ramadan. In addition, the rainy season (July–October) can impact travel [50,51]. A key design feature of the stepped wedge is being able to implement the intervention at precise time points in the calendar. Due to seasonal factors (rainy season) and the religious calendar (Ramadan), this design is likely to be difficult to achieve on a larger scale. As a result, alternative approaches for a definitive trial that might be considered are a randomised cluster or cluster crossover trial. These will be less dependent on specific intervals between starting dates [52].

For peer review only - http://bmjopen.bmj.com/site/about/guidelines.xhtml

In terms of deliverability, we observed relatively high attendance, with 72% of participants attending at least half of the six sessions. More women in urban areas have a job in addition to their house work and many do not live with extended family members who can help with childcare [25]. Therefore, women in the urban areas, like Sukuta, might have more time constraints making it difficult for them to attend, contributing to the lower attendance observed at this clinic. Suggested modifications, such as payment to cover travel, the addition of food, or more potential sessions to attend might help with attendance within an urban context.

The audio and video analysis confirmed the fidelity of the intervention. In general, the intervention was found to be universally acceptable and enjoyable. Interviews and FGDs revealed the reach of the intervention went beyond that of the individuals involved. Participants explained how they had used what they had learned in the intervention sessions to educate other women in their communities. This extension of the intervention has been discussed as one of the benefits of community-led mental health interventions, giving those involved the agency in supporting and teaching others [53].

There was a notable difference in the distribution pattern found with the two measurement tools used, the EPDS and the SRQ-20. This may relate to the items included in the two scales. The SRQ-20 includes somatic items of mental distress (e.g. headaches, shaking hands) while the EPDS purposefully excludes such items. Previous studies have shown that in an African context, it is the somatic symptoms that manifest most clearly in those women

For peer review only - http://bmjopen.bmj.com/site/about/guidelines.xhtml

with particularly raised levels of psychiatric distress, making the SRQ-20 an appropriate tool [33,54]. The SRQ-20's binomial response format, compared to the EPDS likert scale response format, also means that it is more easily administered and understood by respondents with low literacy. However, both scales showed a clear difference between the intervention and Control group suggesting that CHIME had helped reduce both psychological and somatic symptoms.

The differences between the two groups at the post-intervention assessment showed an effect of 2.13 (0.89, 33.38) on SRQ-20 and 1.98 (1.06, 2.90) on EPDS. To our knowledge this makes this study the first to provide information on the size of effect that could be expected from a population level intervention when using the SRQ-20. This finding will not only be helpful for a future scaled up trial using CHIME but also for other population level community mental health interventions trials within LMIC contexts.

Limitations

While the overall recruitment rate was high (81%), fewer women were recruited into the intervention group (*n* = 50) compared to the Control group (*n* = 74). Feedback from the RAs indicated that this was potentially due to the frequency of available clinic days to complete recruitment. Additionally, selection bias was possible. It may have been that those with more work or family pressures might be less likely to be able to afford the time to take off for the intervention. Any future trial would need to address this potential bias. Participants were not blinded to which group they were assigned, increasing the potential for response bias, a problem faced by all research studies that are unblinded and use self-

For peer review only - http://bmjopen.bmj.com/site/about/guidelines.xhtml

reporting [55]. A future study might help reduce the effect of these biases through a different trial design with a more extended recruitment period and a consenting process whereby participants are not aware of their group assignment until after they have agreed to take part.

Participants' baseline antenatal EPDS and SRQ-20 scores were significantly lower in the intervention group compared to the Control group. This difference might have also been due to those with less time constraints, and potentially less stress, being more likely to take part in the intervention. Although these differences could partly be accounted for within the analysis, a blind consenting process could also help lessen their effect within a future trial.

CONCLUSION

We have demonstrated that a community music-based psychosocial intervention (CHIME) is acceptable and feasible to deliver in The Gambia as a way to reduce symptoms of antenatal CMDs. We have identified a potential beneficial effect that will need to be confirmed through a future definitive trial. Post-intervention interviews gave valuable information and feedback on the intervention and operational aspects of the design, payment, transportation and session structure. No previous study has been identified that investigates the potential of a community music intervention for perinatal mental health in a LMIC and the feasibility outcomes reported here highlight the potential of such an approach.

For peer review only

For peer review only - http://bmjopen.bmj.com/site/about/guidelines.xhtml

Ethics approval and consent to participate

Ethical approval was obtained from the Goldsmiths University Ethics Committee, the Gambia Government/MRC Gambia joint ethics committee and the Australian National University ethics committee. Members of the research team carried out the consenting and conduct of this study orally. It was emphasised that any participant was able to withdraw from the study at any point without any consequences.

Competing interests

The authors declare that they have no competing interests.

Funding and sponsorship

This study was funded by the MRC-AHRC Global Public Health: Partnership Awards scheme (MR/R024618/1) awarded to Prof Lauren Stewart. The funders (MRC and AHRC) and sponsor (Goldsmiths) had no roles or responsibilities in the design, conduct, data analysis and interpretation, manuscript writing, and dissemination of results.

Data availability

Deidentified participant data that underline the results reported in this article are available upon reasonable request from the corresponding author (ksanf001@gold.ac.uk).

Author contributions

The manuscript was prepared by KRS and all authors approved the final draft. KRS and BM helped with data management and data collection materials. BD provided local

For peer review only

knowledge and organization in the field liaising with Kanyeleng groups and antenatal clinics. HC provided management of the funds and transportation in The Gambia. HBH and MG completed all data collection and input. KRS completed data cleaning and analysis. VC provided methodological and statistical expertise. VG and PR provided expertise in perinatal mental health. IC and BM provided expertise on music and its role in health and community. All authors, except VG and IC, spent time in The Gambia supervising the project on the ground. LS is the grant holder and principal investigator and oversaw all aspects of the study.

Acknowledgements

We would like to thank the Kanyeleng groups and the participants for taking part in the study. Thank you also to Pa Bakary Sonko, Charlotte Hanlon and Catherine Carr for their essential advice and support and Jane Offerman for helping with the administration of this project.

For peer review only

REFERENCES

1 Fisher J, de Mello MC, Patel V, *et al.* Prevalence and determinants of common perinatal mental disorders in women in low- and lower-middle-income countries: A systematic review. *Bull World Health Organ* 2012;**90**:139–49. doi:10.2471/BLT.11.091850

2 World Health Organization. The international classification of diseases, 10th edition (ICD- 10). Geneva, Swizerland: 2010. doi:10.1016/j.nurpra.2008.09.020

3 Glover V, O'Donnell KJ, O'Connor TG, *et al.* Prenatal maternal stress, fetal programming, and mechanisms underlying later psychopathology - A global perspective. *Dev Psychopathol* 2018;**30**:843–54. doi:10.1017/S095457941800038X

4 Martins C, Gaffan EA. Effects of early maternal depression on patterns of infant–mother attachment: A meta-analytic investigation. *J Child Psychol Psychiatry Allied Discip* 2000;**41**:737–46.

5 Stein A, Pearson RM, Goodman SH, *et al.* Effects of perinatal mental disorders on

For peer review only - http://bmjopen.bmj.com/site/about/guidelines.xhtml

the fetus and child. *Lancet* 2014;**384**:1800–19. doi:10.1016/S0140-6736(14)61277-0

6 Milgrom J, Gemmill AW, Bilszta JL, *et al.* Antenatal risk factors for postnatal depression: A large prospective study. *J Affect Disord* 2008;**108**:147–57. doi:10.1016/j.jad.2007.10.014

7 Robertson E, Grace S, Wallington T, *et al.* Antenatal risk factors for postpartum depression: A synthesis of recent literature. *Gen Hosp Psychiatry* 2004;**26**:289–95. doi:10.1016/j.genhosppsych.2004.02.006

8 Glover V. Prenatal stress and its effects on the fetus and the child: Possible Underlying biological mechanisms. In: Antonelli MC, ed. *Perinatal Programming of Neurodevelopment*. Advances in Neurobiology 2015. 269–83. doi:10.1007/978-1-4939-1372-5_13

9 Ramchandani PG, Richter LM, Norris SA, *et al.* Maternal prenatal stress and later child behavioral problems in an urban South African setting. *J Am Acad Child Adolesc Psychiatry* 2010;**49**:239–47. doi:10.1097/00004583-201003000-00007

10 Rahman A, Fisher J, Bower P, *et al.* Interventions for common perinatal mental disorders in women in low- and middle-income countries: A systematic review and meta-analysis. *Bull World Health Organ* 2013;**91**:593-601I. doi:10.2471/BLT.12.109819

11 Semrau M, Alem A, Abdulmalik J, *et al.* Developing capacity-building activities for mental health system strengthening in low- and middle-income countries for service users and caregivers, service planners, and researchers. *Epidemiol Psychiatr Sci* 2017;:1–11. doi:10.1017/S2045796017000452

12 Semrau M, Lempp H, Keynejad R, *et al.* Service user and caregiver involvement in mental health system strengthening in low- and middle-income countries: Systematic review. *BMC Health Serv Res* 2016;**16**:79. doi:10.1186/s12913-016-1323-8

13 Williams E, Dingle GA, Clift S. A systematic review of mental health and wellbeing outcomes of group singing for adults with a mental health condition. *Eur J Public Health* 2018;**28**:1035–42. doi:10.1093/eurpub/cky115

14 Terry MM, Terry DR. Singing the Blues: A literature review of the effects of music on postnatal depression. *Int J Innov Interdiscip* 2012;**1**:55–67.

15 Wulff V, Hepp P, Fehm T, *et al.* Music in obstetrics: An intervention option to reduce tension, pain and stress. *Geburtshilfe Frauenheilkd* 2017;**77**:967–75. doi:10.1055/s-0043-118414

16 Fancourt D, Perkins R. Effect of singing interventions on symptoms of postnatal depression: Three-arm randomised controlled trial. *Br J Psychiatry* 2018;**212**:119–21. doi:10.1192/bjp.2017.29

17 Clift S, Hancox G, Morrison I, *et al.* Group singing as a public health resource. In: Clift S, Camic PM, eds. *Oxford Textbook of Creative Arts, Health, and Wellbeing: International Perspectives on Practice, Policy and Research*. Oxford, UK: : Oxford University Press 2015. 251–8.

18 Dingle GA, Clift S, Finn S, *et al.* An agenda for best practice research on group singing , health , and well-being. *Music Sci* 2019;**2**:1–15. doi:10.1177/2059204319861719

19 Pearce E, Launay J, Dunbar RIM. The ice-breaker effect: Singing mediates fast

For peer review only

social bonding. *R Soc Open Sci* 2015;**2**:150221. doi:10.1098/rsos.150221
20 McConnell BB. Music and health communication in The Gambia: A social capital approach. *Soc Sci Med* 2016;**169**:132–40. doi:10.1016/j.socscimed.2016.09.028
21 Bastien S. Reflecting and shaping the discourse: The role of music in AIDS communication in Tanzania. *Soc Sci Med* 2009;**68**:1357–60. doi:10.1016/j.socscimed.2009.01.030
22 Silver D. Songs and storytelling: Bringing health messages to life in Uganda. *Educ Heal* 2001;**14**:51–60. doi:10.1080/13576280010015362
23 World Bank. The Gambia overview. 2019.https://www.worldbank.org/en/country/gambia/overview (accessed 4 Oct 2019).
24 McConnell BB. Gambia: History, culture, and geography of music. In: Sturman J, ed. *The SAGE International Encyclopedia of Music and Culture*. Thousand Oaks, CA: : SAGE Publications, Inc. 2019. 974–5. doi:10.4135/9781483317731.n305
25 The Gambia Bureau of Statistics (GBOS) and ICF International. The Gambia Demographic and Health Survey 2013. Banjul, The Gambia and Rockville, Maryland, USA: 2014.
26 World Bank. World development indicators 2015. Washington DC: 2015. doi:10.1596/978–1-4648–0440–3.
27 McConnell BB. *Singing the unsayable: Female performers and global health in The Gambia*. 2015.
28 McConnell BB. Performing ‘participation’: Kanyeleng musicians and global health in The Gambia. *Ethnomusicology* 2017;**61**:312–32.
29 Sanfilippo KRM, McConnell B, Cornelius V, *et al.* A study protocol for testing the feasibility of a randomised stepped wedge cluster design to investigate a Community Health Intervention through Musical Engagement (CHIME) for perinatal mental health in The Gambia. *Pilot Feasibility Stud* 2019;**5**:124. doi:10.1186/s40814-019-0515-5
30 Eldridge SM, Chan CL, Campbell MJ, *et al.* CONSORT 2010 statement: Extension to randomised pilot and feasibility trials. *BMJ* 2016;**355**. doi:10.1136/bmj.i5239
31 Sim J, Lewis M. The size of a pilot study for a clinical trial should be calculated in relation to considerations of precision and efficiency. *J Clin Epidemiol* 2012;**65**:301–8. doi:10.1016/j.jclinepi.2011.07.011
32 World Health Organization. Process of translation and adaptation of instruments. 2014.
33 Hanlon C, Medhin G, Alem A, *et al.* Detecting perinatal common mental disorders in Ethiopia: Validation of the self-reporting questionnaire and Edinburgh Postnatal Depression Scale. *J Affect Disord* 2008;**108**:251–62. doi:10.1016/j.jad.2007.10.023
34 Cox JL, Holden J, Henshaw C. *Perinatal mental health: The Edinburgh Postnatal Depression Scale (EPDS) manual*. 2nd ed. London, UK: : The Royal College of Psyciatrists Publications 2014.
35 Beusenberg M, Orley J. A user’s guide to the Self Reporting Questionnaire (SRQ). 1994.
36 Sawyer A, Ayers S, Smith H. Pre- and postnatal psychological wellbeing in Africa: A systematic review. *J Affect Disord* 2010;**123**:17–29.

For peer review only - http://bmjopen.bmj.com/site/about/guidelines.xhtml

doi:10.1016/j.jad.2009.06.027
37 Kozinszky Z, Dudas RB. Validation studies of the Edinburgh Postnatal Depression Scale for the antenatal period. *J Affect Disord* 2015;**176**:95–105. doi:10.1016/j.jad.2015.01.044
38 Tsai AC, Scott JA, Hung KJ, *et al.* Reliability and validity of instruments for assessing perinatal depression in African settings: Systematic review and meta-analysis. *PLoS One* 2013;**8**:e82521. doi:10.1371/journal.pone.0082521
39 Coleman R, Morison L, Paine K, *et al.* Women's reproductive health and depression: A community survey in The Gambia, West Africa. *Soc Psychiatry Psychiatr Epidemiol* 2006;**41**:720–7. doi:10.1007/s00127-006-0085-8
40 Nabwera HM, Moore SE, Mwangome MK, *et al.* The influence of maternal psychosocial circumstances and physical environment on the risk of severe wasting in rural Gambian infants: A mixed methods approach. *BMC Public Health* 2018;**18**:109. doi:10.1186/s12889-017-4984-2
41 Bartram C. *Early infant social behaviour and maternal perinatal depression in Kiang West , The Gambia*. 2018.
42 Chan AW, Tetzlaff JM, Gøtzsche PC, *et al.* SPIRIT 2013 explanation and elaboration: Guidance for protocols of clinical trials. *BMJ* 2013;**346**:e7586. doi:10.1136/bmj.e7586
43 van Buuren S, Groothuis-Oudshoorn K. mice: Multivariate imputation by chained equations in R. *J Stat Softw* 2011;**45**:1–67.
44 R Core Team. R: A language and environment for statistical computing. 2013.http://www.r-project.org/
45 Braun V, Clarke V. Using thematic analysis in psychology. *Qual Res Psychol* 2006;**3**:77–101. doi:10.1191/1478088706qp063oa
46 Dedoose version 7.0.23. Dedoose. dedoose.com. 2016.
47 McConnell BB. *Music, health, and power: Singing the unsayable in The Gambia*. New York, NY: : Routledge 2020. doi:10.4324/9780367312732
48 Saho B. Ritualizing and domesticating space: 'Kañeleng' women coping with childlessness in the Gambia. *Mand Stud* 2012;**14**:99–125. doi:10.2307/44080973
49 Petersen I, Evans-Lacko S, Semrau M, *et al.* Promotion, prevention and protection: Interventions at the population- and community-levels for mental, neurological and substance use disorders in low- and middle-income countries. *Int J Ment Health Syst* 2016;**10**:30. doi:10.1186/s13033-016-0060-z
50 Fardi SA. *Neonatal hair cortisol in relation to maternal environment, sociodemographic factors, and infant birth and growth outcomes in rural Gambia*. 2017.
51 Moore SE, Cole TJ, Poskitt EME, *et al.* Season of birth predicts mortality in rural Gambia. *Nature* 1997;**388**:434. doi:10.1038/41245
52 Arnup SJ, McKenzie JE, Hemming K, *et al.* Understanding the cluster randomised crossover design: A graphical illustration of the components of variation and a sample size tutorial. *Trials* 2017;**18**:381. doi:10.1186/s13063-017-2113-2
53 Kohrt BA, Asher L, Bhardwaj A, *et al.* The role of communities in mental health care in low-and middle-income countries: A meta-review of components and competencies. *Int J Environ Res Public Health* 2018;**15**:1279. doi:10.3390/ijerph15061279

For peer review only - http://bmjopen.bmj.com/site/about/guidelines.xhtml

54 Senturk V, Hanlon C, Medhin G, *et al.* Impact of perinatal somatic and common mental disorder symptoms on functioning in Ethiopian women: The P-MaMiE population-based cohort study. *J Affect Disord* 2012;**136**:340–9. doi:10.1016/j.jad.2011.11.028

55 Chan D. So why ask me? Are self-report data really that bad? In: Lance CE, Vandenberg RJ, eds. *Statistical and Methodological Myths and Urban Legends: Doctrine, Verity and Fable in the Organizational and Social Sciences*. Routledge/Taylor & Francis Group 2008. 309–36. doi:10.4324/9780203867266

For peer review only

For peer review only - http://bmjopen.bmj.com/site/about/guidelines.xhtml

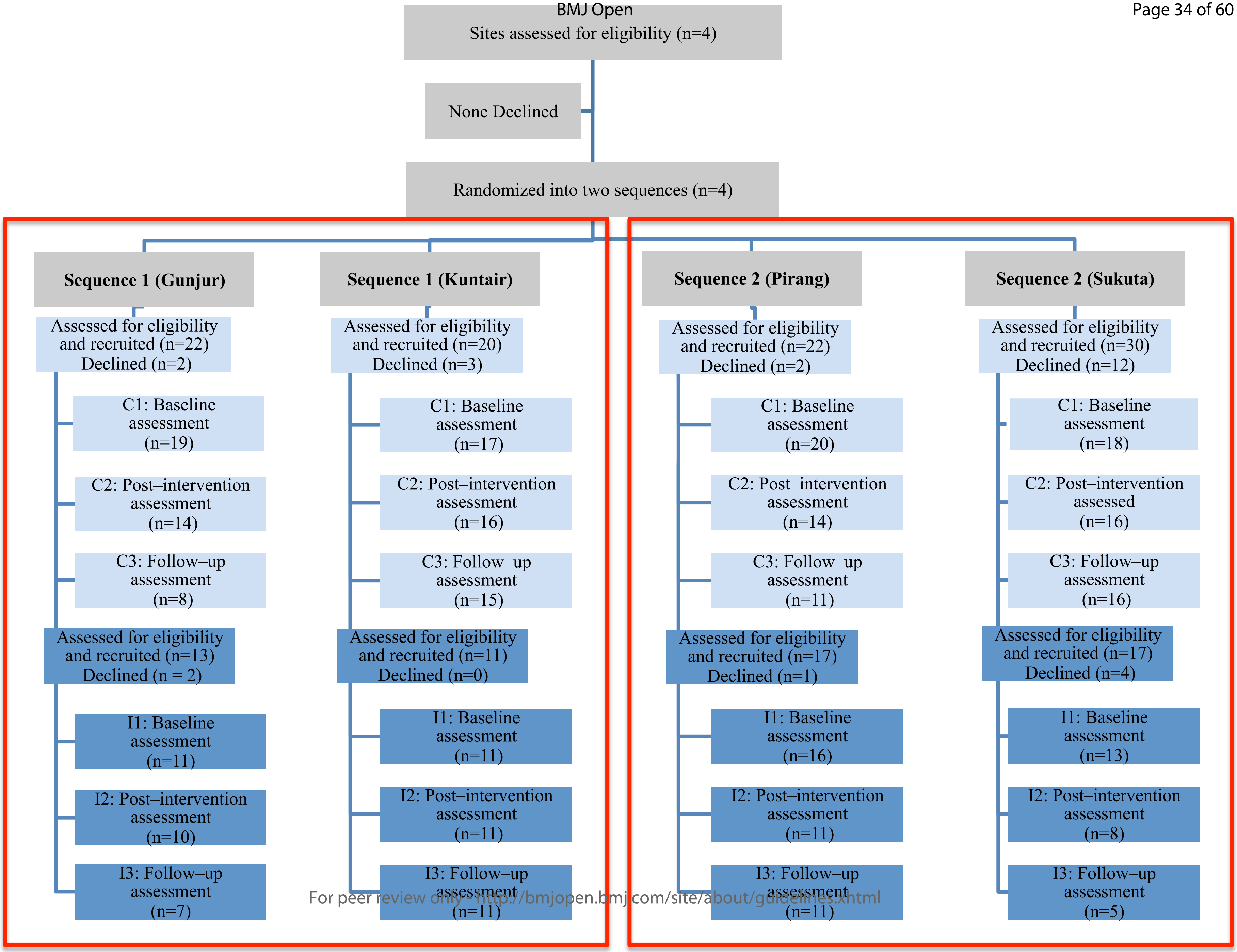

For peer review only - http://bmjopen.bmj.com/site/about/guidelines.xhtml

Planned Timeline

Time point:		1	2	3	4	5	6	7	8	9	10	11	12	13	14	15	16	17	18	19	20	21	22	23	24	25	26	27	28	29	30	31	32	33	34	35	36
Sequence 1	Cluster 1	C 1	X	X	X	X	X	X	C 2				C 3																								
														I 1	O	O	O	O	O	O	I 2				I 3												
	Cluster 2	C 1	X	X	X	X	X	X	C 2				C 3																								
														I 1	O	O	O	O	O	O	I 2				I 3												
Sequence 2	Cluster 3													C 1	X	X	X	X	X	X	C 2				C 3												
																										I 1	O	O	O	O	O	O	I 2				I 3
	Cluster 4													C 1	X	X	X	X	X	X	C 2				C 3												
																										I 1	O	O	O	O	O	O	I 2				I 3

Actual Timeline

Time point:		1	2	3	4	5	6	7	8	9	10	11	12	13	14	15	16	17	18	19	20	21	22	23	24	25	26
Sequence 1	Cluster 1	C 1	X	X	X	X	X	X	C 2				C 3														
											I 1	O	O	O	O	O	O	I 2				I 3					
	Cluster 2	C 1	X	X	X	X	X	X	C 2				C 3														
											I 1	O	O	O	O	O	O	I 2				I 3					
Sequence 2	Cluster 3						C 1	X	X	X	X	X	X	C 2				C 3									
																I 1	O	O	O	O	O	O	I 2				I 3
	Cluster 4						C 1	X	X	X	X	X	X	C 2				C 3									
																I 1	O	O	O	O	O	O	I 2				I 3

Time point = (1 week)

C = Control

I = Intervention

C1 = Control group baseline assessment

C2 = Control group post–intervention assessment

C3 = Control group follow–up assessment

I1 = Intervention group baseline assessment

I2 = Intervention group post–intervention assessment

I3 = Intervention group follow–up assessment

Supplementary Material 1

Intervention Development Workshop Discussion points

These six sessions are intended to support women emotionally and psychologically during their pregnancy.

Session Goals
During the session, we hope that women will participate by singing songs, clapping hands, and moving to the music. Also, we would like you to share some brief information about how to cope with common physical and psychological symptoms of pregnancy. We hope that the women will finish the session

- in a *positive mood*
- feeling part of a socially-supportive group
- with some new strategies of how to cope with some of the common physical and psychological challenges of pregnancy
- equipped with skills to seek wider social support even outside the sessions

Key Messages
Our suggestion is that sessions concentrate on various different themes as follows [discuss with the Kanyeleng group whether these topics seem suitable, allow them to share examples of songs they could use and additional ideas they may have]:

1) Common physical and psychological symptoms of pregnancy and ways to cope such as:

- Importance of the singing group in supporting each other
- Importance of other positive relationships in their lives
- Resilience to challenges and empowerment
- Importance of being open, removing stigma to discuss challenges

2) Learning lullabies for after birth
3) How to care for the baby after birth

Session Structure
A typical session might involve:

- A welcome song at the start
- A middle section that would include one song that would focus on the topic of the week (see above), and others to promote a positive mood and encourage the women to move together.
- A closing song to draw the session to a close

For peer review only

How to run a successful session

Throughout the sessions we would like you to act as 'facilitators' rather than 'teachers'. We would like you to:

- Draw on your existing skills and ways of working to help us fulfill the goals described above.

- Encourage the women to be involved in whatever way they would like (please be aware the some women may not be comfortable to move very vigorously, and should feel encouraged to move in whatever way is comfortable). If someone is not joining in, check if they are okay, and try to engage them in a way that doesn't make them self-conscious.

- Notice how the women are responding to the activities that you initiate – is the pace too slow, too fast, too loud etc.? Try to adapt what you are doing according to the responses you see.

- Although it is fine to mention that pregnancy can involve challenges, it is important not to encourage the women to dwell on these, but instead to think about coping strategies and supporting each other, to ensure that the mood is a positive one.

For peer review only

Supplementary Material 2

SRQ-20
Mandinka

The following questions are related to certain pains and problems that may have bothered you in the last 30 days. If you think the question applies to you and you have had the described problem in the last 30 days, answer YES.
On the other hand, if the question does not apply to you and you did not have the problem in the last 30 days, answer NO.
If you are unsure about how to answer a question, please give the best answer you can.

Nying nyininkari dantangolu i be dending dimoo doolu la aning mantooro doolu la meng y'a long a si ke a ye batandi nying tili tansabo meng tambita.
N'ii y'a mira ko nyininkaroo nying ye maa le, aning i ye nying mantooro sifa soto nying tili tansabo kono, i s'a fo haa.
N'ii y'a tara nyininkaroo nying m'ee maa, aning i mang nying mantooro sifa soto nying tili tansabo kono, i s'a fo haani.

1. Do you often have headaches?<br><br>1. Fo i ka kundimoo soto le waati ning waati?	☐1. Yes	☐0. No
2. Is you appetite poor?<br><br>2. Fo domoro buka diyaa i daa kono?	☐1. Yes	☐0. No
3. Do you sleep badly?<br><br>3. Fo ila siinoo buka beteyaa le bang?	☐1. Yes	☐0. No

For peer review only

4. Are you easily frightened?<br><br>4. Fo i ka tariyaa ka silla le bang?	☐1. Yes	☐0. No
5. Do your hands shake?<br><br>5. Fo i buloolu ka jarajara?	☐1. Yes	☐0. No
6. Do you feel nervous, tense or worried?<br><br>6. I ka fitii le bang wara sondomoo tenkung baliyaa k'e batandi le bang?	☐1. Yes	☐0. No
7. Is your digestion poor?<br><br>7. Fo n'ii ye domoro ke a buka tariyaa ka jii le bang?	☐1. Yes	☐0. No
8. Do you have trouble thinking clearly?<br><br>8. Fo miralotenkung baliyaa k'e batandi le bang?	☐1. Yes	☐0. No
9. Do you feel unhappy?<br><br>9. Fo i ka niitoroo soto le bang?	☐1. Yes	☐0. No

For peer review only

10. Do you cry more than usual?<br><br>10. Fo i ka kumboo danna tambiringo ke le bang?	☐1. Yes	☐0. No
11. Do you find it difficult to enjoy your daily activities?<br><br>11. Fo i buka kontan ka ila lung o lung haajoolu ke le bang?	☐1. Yes	☐0. No
12. Do you find it difficult to make decisions?<br><br>12. Fo I ka bataa kuwo soto le ka hajoolu t'ee i fango ye?	☐1. Yes	☐0. No
13. Is your daily work suffering?<br><br>13. I ka naasoo soto ila lung o lung dookuwo to bang?	☐1. Yes	☐0. No
14. Are you unable to play a useful part in life?<br><br>14. Fo i ka koleya kuwo soto ka nafaa kuwolu tamandi dunia baluwo kono?	☐1. Yes	☐0. No
15. Have you lost interest in things?<br><br>15. Fo ila lafoo talaata fengolu to i ka lafi meng na nung?	☐1. Yes	☐0. No

For peer review only

16. Do you feel that you are a worthless person?<br><br>16. Fo i k'e fango je ko i nafaa soto bang?	☐1. Yes	☐0. No
17. Has the thought of ending your life been on your mind?<br><br>17. Fo waati do ka soto i k'e mira pur ka i fango faa?	☐1. Yes	☐0. No
18. Do you feel tired all the time?<br><br>18. Fo i ka tara bataring waati o waati le bang?	☐1. Yes	☐0. No
19. Do you have uncomfortable feelings in your stomach?<br><br>19. Fo i kono k'e batandi le bang?	☐1. Yes	☐0. No
20. Are you easily tired?<br><br>20. Fo i ka tariyaa ka bataa?	☐1. Yes	☐0. No

For peer review only

SRQ-20
Wolof

The following questions are related to certain pains and problems that may have bothered you in the last 30 days. If you think the question applies to you and you have had the described problem in the last 30 days, answer YES.
On the other hand, if the question does not apply to you and you did not have the problem in the last 30 days, answer NO.
If you are unsure about how to answer a question, please give the best answer you can.

Lache yii daffa khetog meetit walla jeffeh-jeffeh yii nga khamneh manala sohnal si birr fenwerry-fen yu passeh. So fongehneh lache bi andana ak sa halat teh am nga meetit yii ak jeffeh-jeffeh yii si fenwerry-fen yu passeh, tontuma WAAW
Si benen borrbi, su lache bi andutak lee nga halat si yaw teh amulo jeffeh-jeffeh yii si birr fenwerry-fen yu passeh, tontma DADET
Su feekeh danga ham nyarry khel si negga warra tontor lache bi, ngela wyyy jokhma tontu bi nga fogneh mo ngane.

1. Do you often have headaches?<br><br>1. Ndakh dang de ham bopa bu meeti lego lake?	☐1. Yes	☐0. No
2. Is you appetite poor?<br><br>2. Ndakh sa leeka bi daffa wanyekou?	☐1. Yes	☐0. No
3. Do you sleep badly?<br><br>3. Ndakh dang de ham nelew bu bon?	☐1. Yes	☐0. No

For peer review only

4. Are you easily frightened?<br><br>4. Ndakh danga de gawa tit?	□1. Yes	□0. No
5. Do your hands shake?<br><br>5. Ndakh sa lohoyee daff de lokh?	□1. Yes	□0. No
6. Do you feel nervous, tense or worried?<br><br>6. Ndakh dang de yeek titangeh wala d jahleh?	□1. Yes	□0. No
7. Is your digestion poor?<br><br>7. Ndakh dang de ham leeka budut gawa ress?	□1. Yes	□0. No
8. Do you have trouble thinking clearly?<br><br>8. Ndakh dang de ham jeffeh-jeffeh si halat bu bakh?	□1. Yes	□0. No
9. Do you feel unhappy?<br><br>9. Ndakh dang de nyaka kontan?	□1. Yes	□0. No

For peer review only

10. Do you cry more than usual?<br><br>10. Ndakh dang de bug d joi lu barre?	☐1. Yes	☐0. No
11. Do you find it difficult to enjoy your daily activities?<br><br>11. Ndakh danga de ham jeffeh-jeffeh purr ham kontateh si leenga warra deff si bess bi?	☐1. Yes	☐0. No
12. Do you find it difficult to make decisions?<br><br>12. Ndakh dang de ham jeffeh-jeffeh purr deff saye halati boppa?	☐1. Yes	☐0. No
13. Is your daily work suffering?<br><br>13. Ndakh dor de ham khat-khat si sa leugue bess bi?	☐1. Yes	☐0. No
14. Are you unable to play a useful part in life?<br><br>14. Ndakh dang de ham jeffeh-jeffeh si lo hmneh dinala njering sisa ngerre dundu?	☐1. Yes	☐0. No
15. Have you lost interest in things?<br><br>15. Ndakh dor de nyaka eteh si lega warra deff	☐1. Yes	☐0. No

For peer review only

16. Do you feel that you are a worthless person?<br><br>16. Ndakh dang de ham yeek-yeek ne yaw dor darra?	☐1. Yes	☐0. No
17. Has the thought of ending your life been on your mind?<br><br>17. Ndakh danga de tog di halat neh dehh mo la ngenal?	☐1. Yes	☐0. No
18. Do you feel tired all the time?<br><br>18. Ndakh dang de soona wakhtu bu neeka?	☐1. Yes	☐0. No
19. Do you have uncomfortable feelings in your stomach?<br><br>19. Ndakh dang de ham yeek-yeek ngi jeffeh-jeffeh birr?	☐1. Yes	☐0. No
20. Are you easily tired?<br><br>20. Ndakh dang de gawa soona?	☐1. Yes	☐0. No

For peer review only

EPDS
Mandinka

Please tick the answer which comes closest to how you have felt *in the past week, not just how you feel today*. There are no right or wrong answers and please feel free to answer honestly as these questionnaires are confidential.

Bao saaying kono bee la, waranta i wuluuta a mang mee, m be lafi la ka a long i be nyaadi le. M be nyininkaroo ke la ka juubee i be nyaadii le tili worowulo nying kono, a manke i ye i fango je nyaameng bii damma.

1. Have you been able to laugh and see the funny side of things?<br><br>1. Fo i ka jele aning fo i ka jelekuwolu fanang kalamuta?	☐0.<br><br>As much as I always could<br><br>Haa, baake fango n lafita ala nyaameng	☐1.<br><br>Not quite so much now<br><br>A buka siyaa baake ko a be nung nyaameng	☐2.<br><br>Definitely not so much now<br><br>A buka siyaa fereng	☐3.<br><br>Not at all<br><br>M buka jele fereng
2. Have you looked forward with enjoyment to things?<br><br>2. Fo i ka niidiyaa kuwolu jamaa le nyaatosii?	☐0.<br><br>As much as I ever did<br><br>Haa, baake fango n lafita ala nyaameng	☐1.<br><br>Rather less than I used to<br><br>A buka siyaa baake ko a be nung nyaameng	☐2.<br><br>Definitely less than I used to<br><br>A buka siyaa fereng	☐3.<br><br>Hardly at all<br><br>Haani, m buka feng nyaatosii

For peer review only

3. Have you blamed yourself unnecessarily when things went wrong?<br><br>3. Fo i k'e fango jalai kensengke le ning kuwolu mang taa a nyaama?	☐3.<br><br>Yes, most of the time<br><br>Haa, waati jamaa	☐2.<br><br>Yes, some of the time<br><br>Haa, waati doolu	☐1.<br><br>Not very often<br><br>Haani, a mang siyaa	☐0.<br><br>No, never<br><br>Haani, mbuka wo ke fereng
4. Have you been anxious or worried for no good reason?<br><br>4. Fo i ka fiti le waranta i ka tara detering ne i fango feyi a ye a tara a mang jara wo la?	☐0.<br><br>No, not at all<br><br>Haani, m buka wo ke fereng	☐1.<br><br>Hardly ever<br><br>Haani, a mang siyaa	☐2.<br><br>Yes, sometimes<br><br>Haa, waati doolu	☐3.<br><br>Yes, very often<br><br>Haa, waati jamaa
5. Have you felt scared or panicky for no good reason?<br><br>5. Fo i ka silla wara i kijo k'e fara a y'a tara a mang jara wo la?	☐3.<br><br>Yes, quite a lot<br><br>Haa, waati jamaa	☐2.<br><br>Yes, sometimes<br><br>Haa, waati doolu	☐1.<br><br>No, not much<br><br>Haani, a buka siyaa	☐0.<br><br>No, not at all<br><br>Haani, mbuka wo ke

For peer review only

6. Have things been getting on top of you?<br><br>6. Hajoolu k'e detendi le fo a ka koleyaa i bulu ka ila kuwolu tamandi?	☐3.<br><br>Yes, most of the time I haven't been able to cope at all<br><br>Haa, jamaa jamaa a ka koleyaa m bulu	☐2.<br><br>Yes, sometimes I haven't been coping as well as usual<br><br>Haa, waati doolu a ka koleyaa	☐1.<br><br>No, most of the time I have coped quite well<br><br>Haani, jamaa jamaa a buka koleya m bulu	☐0.<br><br>No, I have been coping as well as ever<br><br>Haani, n ka nna kuwolu tamandi noo n ka ke nung nyaameng
7. Have you been so unhappy that you have had difficulty sleeping?<br><br>7. Fo kontaani baliyaa le ka tinna i buka siinoo noo a nyaama?	☐3.<br><br>Yes, most of the time<br><br>Haa, waati o waati	☐2.<br><br>Yes, sometimes<br><br>Haa, waati doolu	☐1.<br><br>Not very often<br><br>Haani, a buka siyaa	☐0.<br><br>No, not at all<br><br>Haani, wo mang soto
8. Have you felt sad or miserable?<br><br>8. Fo niikuyaa waranta niitooroo k'e batandi le bang?	☐3.<br><br>Yes, most of the time<br><br>Haa, waati o waati	☐2.<br><br>Yes, quite often<br><br>Haa, waati doolu	☐1.<br><br>Not very often<br><br>Haani, a buka siyaa	☐0.<br><br>No, not at all<br><br>Haani, wo buka m batandi

For peer review only

9. Have you been so unhappy that you have been crying?<br><br>9. Fo i ka tara kontaani baliyaa le kono fo i ka kumboo?	☐3.<br><br>Yes, most of the time<br><br>Haa, waati jamaa	☐2.<br><br>Yes, quite often<br><br>Haa, waati ning waati	☐1.<br><br>Only occasionally<br><br>Haa, bari a mang siyaa	☐0.<br><br>No, never<br><br>Haani, a mang soto
10. Has the thought of harming yourself occurred to you?<br><br>10. Fo waati doo ka soto i k'e mira pur ka kuu jawo ke i fang na?	☐3.<br><br>Yes, quite often<br><br>Haa, sinyaa jamaa	☐2.<br><br>Sometimes<br><br>Haa, waati doolu	☐1.<br><br>Hardly ever<br><br>Haani, a mang siyaa fereng	☐0.<br><br>Never<br><br>Haani, a mang soto

For peer review only

EPDS
Wolof

Please tick the answer which comes closest to how you have felt *in the past week,* not just how you feel today. There are no right or wrong answers and please feel free to answer honestly as these questionnaires are confidential.

Kom nu nga neeke geugeni birr ne wala musa ham doom, dange bugga ham sa yeek-yeek. Ndimbaleh nyu nga hol tontu bu ngena jageh si sa yeek-yeek si birr bess bu ai bu passeh. Kom neekut le ngai yeek tai.

1. Have you been able to laugh and see the funny side of things?<br><br>1. Ndakh yaw dang nga deh muna rreh sor gisseh lula rretanlo?	☐0.<br><br>As much as I always could<br><br>Waaw, lu si baari kom nu ma ko muneh	☐1.<br><br>Not quite so much now<br><br>Waaw, neekut lu bareh kom nonu leegue	☐2.<br><br>Definitely not so much now<br><br>Degga-degga neekut lu bareh sah leegue	☐3.<br><br>Not at all<br><br>Amut sah
2. Have you looked forward with enjoyment to things?<br><br>2. Ndakh yaw dang deh muna saintu yo hamneh munnala kontanlo?	☐0.<br><br>As much as I ever did<br><br>Waaw, kom numa ko musa deffeh	☐1.<br><br>Rather less than I used to<br><br>Waaw, lu meelut kom nu ma ko dan deffeh	☐2.<br><br>Definitely less than I used to<br><br>Degga-degga neekut kom numa kodan deffeh	☐3.<br><br>Hardly at all<br><br>Waaw, su ameh sah barewut

For peer review only

3. Have you blamed yourself unnecessarily when things went wrong?<br><br>3. Ndakh yaw dang deh am sikka nge si sa boppa su mbirr dameh nom waruta demeh?	☐3.<br><br>Yes, most of the time<br><br>Waaw, si wakhtu yu bareh	☐2.<br><br>Yes, some of the time<br><br>Waaw, si yeena sayee	☐1.<br><br>Not very often<br><br>Neekut lu bareh	☐0.<br><br>No, never<br><br>Dadet, musuta ham
4. Have you been anxious or worried for no good reason?<br><br>4. Ndakh dang de ham jahleh walla hel bu dalut si li ko jarut?	☐0.<br><br>No, not at all<br><br>Dadet musuta ham	☐1.<br><br>Hardly ever<br><br>Su ameh yet bareh wut	☐2.<br><br>Yes, sometimes<br><br>Waaw, yeena sayee	☐3.<br><br>Yes, very often<br><br>Waaw, si wahktu yu bareh
5. Have you felt scared or panicky for no good reason?<br><br>5. Ndakh danga de ham ragal walla titangeh si lu ko jarut?	☐3.<br><br>Yes, quite a lot<br><br>Waaw, si wakhtu yu bareh	☐2.<br><br>Yes, sometimes<br><br>Waaw, yeena sayee	☐1.<br><br>No, not much<br><br>Dadet, barehwut	☐0.<br><br>No, not at all<br><br>Dadet, musuta ham sah

For peer review only

6. Have things been getting on top of you?<br><br>6. Ndakh Jeffeh-jeffeh daffa bareh si sa boopa bi be-nga hamneh anta nulo ko?	☐3.<br><br>Yes, most of the time I haven't been able to cope at all<br><br>Waaw, yeena sayee duma henu jeffeh-jeffeh yoyu	☐2.<br><br>Yes, sometimes I haven't been coping as well as usual<br><br>Waaw, yeena sayee duma deh muna henu jeffeh-jeffeh yoyu kom num wara demeh	☐1.<br><br>No, most of the time I have coped quite well<br><br>Dadet, yeena sayee dama deh muna henu jeffeh- jeffeh yoyu	☐0.<br><br>No, I have been coping as well as ever<br><br>Dadet, dama deh henu jeffeh-jeffeh yoyu kom nom musa mel
7. Have you been so unhappy that you have had difficulty sleeping?<br><br>7. Ndakh danga de ham nyaka kontante (walla nakhar) be takh dor deh muna nelew?	☐3.<br><br>Yes, most of the time<br><br>Waaw, wakhtu bu neeka	☐2.<br><br>Yes, sometimes<br><br>Waaw, yeena sayee	☐1.<br><br>Not very often<br><br>Neekut lu bareh	☐0.<br><br>No, not at all<br><br>Dadet, musuta ham sah
8. Have you felt sad or miserable?<br><br>8. Ndakh danga de yeek nakhar walla chono bo hamneh daffa doi war?	☐3.<br><br>Yes, most of the time<br><br>Waaw, wakhtu yu bareh	☐2.<br><br>Yes, quite often<br><br>Waaw, yeena sayee	☐1.<br><br>Not very often<br><br>Neekut lu bareh	☐0.<br><br>No, not at all<br><br>Dadet, musuta ham sah

For peer review only

9. Have you been so unhappy that you have been crying?<br><br>9. Ndakh dang de ham nekhar bula deh joi lo?	☐3.<br><br>Yes, most of the time<br><br>Waaw, wakhtu yu bareh	☐2.<br><br>Yes, quite often<br><br>Waaw, yeena sayee	☐1.<br><br>Only occasionally<br><br>Lu tuti si ai hew-hew	☐0.<br><br>No, never<br><br>Dadet, musuta ham
10. Has the thought of harming yourself occurred to you?<br><br>10. Ndakh am halat purr lurr sa boopa daff deh nyaw sisa hell bi?	☐3.<br><br>Yes, quite often<br><br>Waaw, si wahktu yu bareh	☐2.<br><br>Sometimes<br><br>Yeena sayee	☐1.<br><br>Hardly ever<br><br>Suma amet het barewut	☐0.<br><br>Never<br><br>Musuta ham

For peer review only

Supplementary Material 3

CHIME session Check-List

1. Did any of the songs address any of the following topics? Y/N (Circle which ones below)
   a. Importance of the singing group in supporting each other
   b. Importance of other positive relationships in their lives
   c. Resilience to challenges and empowerment
   d. Importance of being open, removing stigma to discuss challenges

2. Did the sessions dwell on the challenges of pregnancy or more the coping mechanisms and resilience? Y/N

3. Did the kanyeleng group incorporate a lullaby? If so, which? Y/N
   a. Lullaby name:
4. Did the kanyeleng group explain the benefits of singing lullabies? Y/N

5. Did the kanyeleng group use a welcoming song? If so, which? Y/N
   a. Song name:

6. Did the kanyeleng group use a closing song at the end? If so, which song? Y/N
   a. Song name:

7. Overall, did the participants actively participate in the session by joining in with the singing, dancing or clapping? Y/N

8. Were there any examples where the kanyeleng group acted to encourage women to participate? Y/N
   a. Give example and time point in the video:

For peer review only

Supplementary Material 4

Retention and Attrition Rates Overall and by Group

	Retention			Attrition		
	**Pre** *n* **(%)**	**Post** *n* **(%)**	**Follow-up** *n* **(%)**	**Pre-Post (%)**	**Post-Follow-up (%)**	**Pre-Follow-up (%)**
**All**	124 (100%)	99 (80%)	83 (67%)	20%	16%	33%
**Intervention**	50 (100%)	39 (78%)	33 (66%)	22%	15%	34%
**Control**	74 (100%)	60 (81%)	50 (68%)	19%	17%	32%

For peer review only

Supplementary Material 5

CHIME Session Duration

	Session Duration						
**Site**	**Session 1**	**Session 2**	**Session 3**	**Session 4**	**Session 5**	**Session 6**	**Average**
Gunjur	31.00	45.00	48.00	60.00	57.00	50.00	48.50
Kuntair	42.00	75.00	75.00	70.00	66.00	71.00	66.50
Pirang	75.00	50.00	65.00	55.00	50.00	65.00	60.00
Sukuta	55.00	78.00	64.00	61.00	60.00	60.00	63.00
**Total**	**50.75**	**62.00**	**63.00**	**61.50**	**58.25**	**61.50**	**59.50**

Note. Time is in minutes.

Number of participants Present at Each Session

	Session Attendance						
**Site**	**Total Possible**	**Session 1**	**Session 2**	**Session 3**	**Session 4**	**Session 5**	**Session 6**
Gunjur	11	9	6	7	8	7	8
Kuntair	11	11	10	8	11	11	11
Pirang	16	13	12	11	10	12	11
Sukuta	13	10	5	7	4	4	6

Note. Total Possible is the total amount of women recruited to be in the intervention.

For peer review only

Supplementary Material 6

EPDS and SRQ-20 Scores at Three Time-Points

EPDS	Baseline		Post		Follow-up	
	n	*M(SD)*	*n*	*M(SD)*	*n*	*M(SD)*
All	124	4.25 (4.10)	99	2.56 (2.74)	83	1.80 (2.23)
Intervention	50	2.90 (3.07)	39	1.18 (1.50)	33	0.94 (1.73)
Control	74	5.16 (4.46)	60	3.45 (2.99)	50	2.36 (2.36)
**SRQ-20**	**Baseline**		**Post**		**Follow-up**	
	n	*M(SD)*	*n*	*M(SD)*	*n*	*M(SD)*
All	124	7.27 (4.00)	99	5.31 (4.02)	83	4.25 (3.32)
Intervention	50	6.22 (3.83)	39	3.62 (3.32)	33	2.70 (3.03)
Control	74	7.97 (3.99)	60	6.42 (4.08)	50	5.28 (3.12)

For peer review only

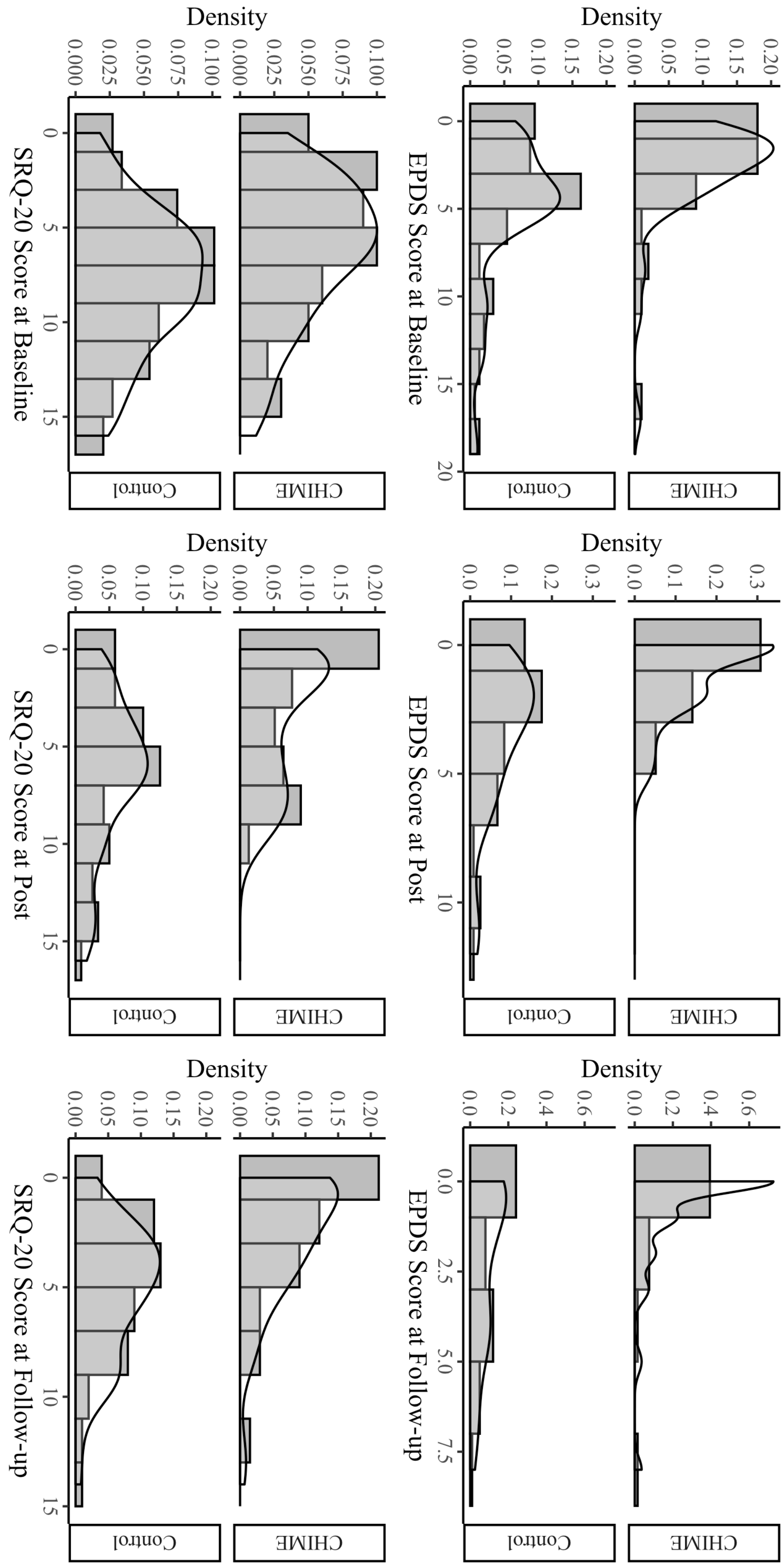
Density
SRQ-20 Score at Baseline
EPDS Score at Baseline
SRQ-20 Score at Post
EPDS Score at Post
SRQ-20 Score at Follow-up
EPDS Score at Follow-up
Control
CHIME

Supplementary Material 7

Mixed Model Results

	Post intervention		Post intervention + 4 weeks follow up	
	**adjusted mean difference (95%CI)**	**p-value**	**adjusted mean difference (95%CI)**	**p-value**
**SRQ-20**	2.13 (0.89, 3.38)	<0.01	2.09 (0.76, 3.42)	<0.01
**EPDS**	1.98 (1.06, 2.90)	<0.01	0.98 (-0.01, 1.97)	0.05

Note. Mixed models adjusted for baseline score and clinic; MD>0 favour intervention arm

For peer review only

CONSORT 2010 checklist of information to include when reporting a pilot or feasibility trial*

Section/Topic	Item No	Checklist item	Reported on page No
**Title and abstract**			
	1a	Identification as a pilot or feasibility randomised trial in the title	Pg. 1
	1b	Structured summary of pilot trial design, methods, results, and conclusions (for specific guidance see CONSORT abstract extension for pilot trials)	Pg. 2
**Introduction**			
Background and objectives	2a	Scientific background and explanation of rationale for future definitive trial, and reasons for randomised pilot trial	Pgs. 4-6
	2b	Specific objectives or research questions for pilot trial	Pg. 7
**Methods**			
Trial design	3a	Description of pilot trial design (such as parallel, factorial) including allocation ratio	Pg. 7
	3b	Important changes to methods after pilot trial commencement (such as eligibility criteria), with reasons	NA
Participants	4a	Eligibility criteria for participants	Pg. 8
	4b	Settings and locations where the data were collected	Pg. 9
	4c	How participants were identified and consented	Pg. 8
Interventions	5	The interventions for each group with sufficient details to allow replication, including how and when they were actually administered	Pgs. 9-10
Outcomes	6a	Completely defined prespecified assessments or measurements to address each pilot trial objective specified in 2b, including how and when they were assessed	Pgs. 11-12
	6b	Any changes to pilot trial assessments or measurements after the pilot trial commenced, with reasons	NA
	6c	If applicable, prespecified criteria used to judge whether, or how, to proceed with future definitive trial	NA
Sample size	7a	Rationale for numbers in the pilot trial	Pg. 9.
	7b	When applicable, explanation of any interim analyses and stopping guidelines	NA
Randomisation:			Pgs. 7-8
Sequence generation	8a	Method used to generate the random allocation sequence	Pg. 7
	8b	Type of randomisation(s); details of any restriction (such as blocking and block size)	Pg. 7
Allocation concealment mechanism	9	Mechanism used to implement the random allocation sequence (such as sequentially numbered containers), describing any steps taken to conceal the sequence until interventions were assigned	Pg. 7

For peer review only

Implementation	10	Who generated the random allocation sequence, who enrolled participants, and who assigned participants to interventions	Pg. 7
Blinding	11a	If done, who was blinded after assignment to interventions (for example, participants, care providers, those assessing outcomes) and how	NA. Not blinded study.
	11b	If relevant, description of the similarity of interventions	NA
Statistical methods	12	Methods used to address each pilot trial objective whether qualitative or quantitative	Pgs. 13-14
**Results**			
Participant flow (a diagram is strongly recommended)	13a	For each group, the numbers of participants who were approached and/or assessed for eligibility, randomly assigned, received intended treatment, and were assessed for each objective	Pg. 16
	13b	For each group, losses and exclusions after randomisation, together with reasons	Pg. 16
Recruitment	14a	Dates defining the periods of recruitment and follow-up	
	14b	Why the pilot trial ended or was stopped	NA
Baseline data	15	A table showing baseline demographic and clinical characteristics for each group	Pg. 12
Numbers analysed	16	For each objective, number of participants (denominator) included in each analysis. If relevant, these numbers should be by randomised group	Pgs. 21-22
Outcomes and estimation	17	For each objective, results including expressions of uncertainty (such as 95% confidence interval) for any estimates. If relevant, these results should be by randomised group	Pgs. 21-22
Ancillary analyses	18	Results of any other analyses performed that could be used to inform the future definitive trial	Pgs. 14-22
Harms	19	All important harms or unintended effects in each group (for specific guidance see CONSORT for harms)	Pg. 21
	19a	If relevant, other important unintended consequences	NA
**Discussion**			
Limitations	20	Pilot trial limitations, addressing sources of potential bias and remaining uncertainty about feasibility	Pgs. 25-26
Generalisability	21	Generalisability (applicability) of pilot trial methods and findings to future definitive trial and other studies	Pgs. 22-25
Interpretation	22	Interpretation consistent with pilot trial objectives and findings, balancing potential benefits and harms, and considering other relevant evidence	Pgs. 22-25
	22a	Implications for progression from pilot to future definitive trial, including any proposed amendments	Pgs. 22-25
**Other information**			
Registration	23	Registration number for pilot trial and name of trial registry	Pg. 2
Protocol	24	Where the pilot trial protocol can be accessed, if available	Pg. 7
Funding	25	Sources of funding and other support (such as supply of drugs), role of funders	Pg. 28
	26	Ethical approval or approval by research review committee, confirmed with reference number	Pg. 28

For peer review only
